# Supplementary material for: Dantrolene corrects cellular disease features of Darier disease and may be a novel treatment
Source: EMBO Mol Med. 2024 Jul 26;16(9):1986–2001. doi: 10.1038/s44321-024-00104-3 (PMC11392931; doi:10.1038/s44321-024-00104-3)
Supplement: Supplementary file 1 — Appendix [file 44321_2024_104_MOESM1_ESM.pdf]

Appendix

Table of Content

Appendix Figure S1 – DD patient RNA quality assessment – 2-19

Appendix Figure S2 – Cell type deconvolution – 20-22

Appendix Figure S3 – List of primers – 23

Assay Class: Eukaryote Total RNA Nano
Data Path: C:\...Eukaryote Total RNA Nano_DE13701055_2021-08-27_13-27-31.xad

Created: 8/27/2021 1:27:30 PM
Modified: 8/27/2021 1:51:20 PM

Electrophoresis File Run Summary

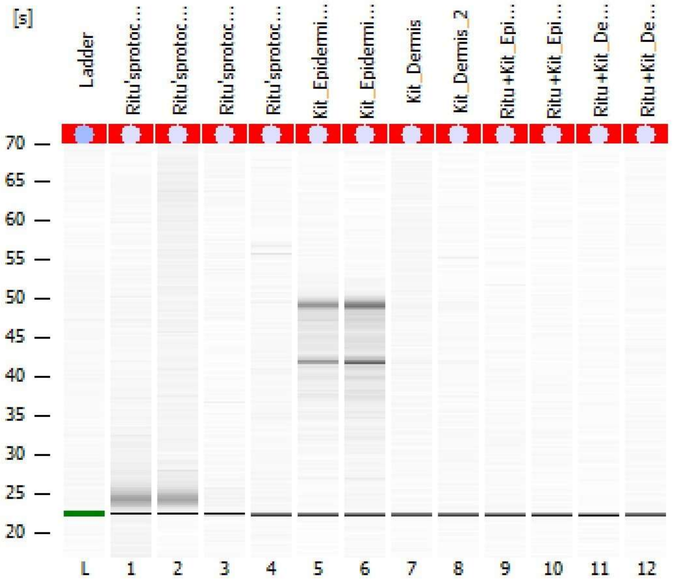

Instrument Information:

Instrument Name: com2
Firmware: C.01.069
Serial#: DE13701055
Type: G2938B

Assay Information:

Assay Origin Path: C:\Program Files (x86)\Agilent\2100 bioanalyzer\2100 expert\assays\RNA\Eukaryote Total RNA Nano Series II.xsy

Assay Class: Eukaryote Total RNA Nano

Version: 2.6

Assay Comments: Total RNA Analysis ng sensitivity (Eukaryote)

© Copyright 2003 - 2009 Agilent Technologies, Inc.

Chip Information:

Chip Lot #:

Reagent Kit Lot #:

Chip Comments:

Ritu'sprotocol_Epidermis+dermis

RIN: 2.50

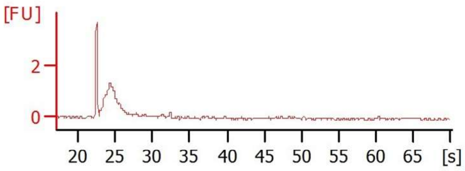

Ritu'sprotocol_Epidermis+dermis_2

RIN: 2.40

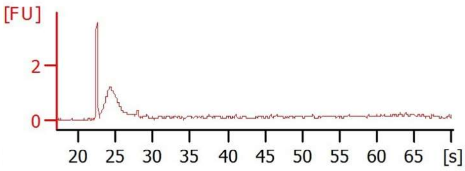

Ritu'sprotocol_dermis

RIN:1

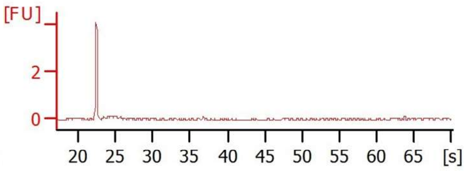

Ritu'sprotocol_dermis_2

RIN:1

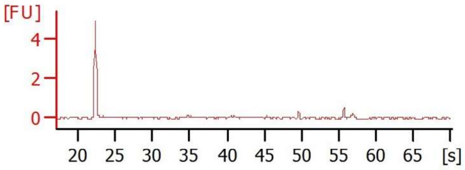

Kit_Epidermis+dermis

RIN: 7.70

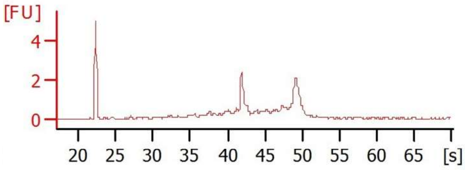

Kit_Epidermis+dermis_2

RIN: 7.40

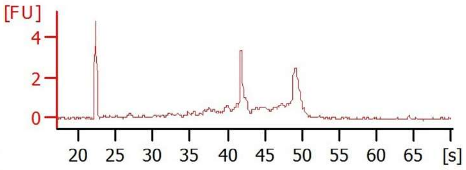

Kit_Dermis

RIN:1

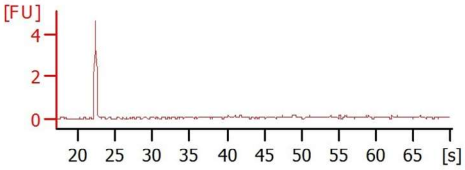

Kit_Dermis_2

RIN:1

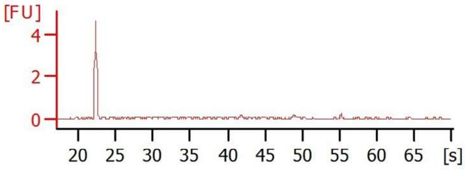

Ritu+Kit_Epidermis+Dermis

RIN:1

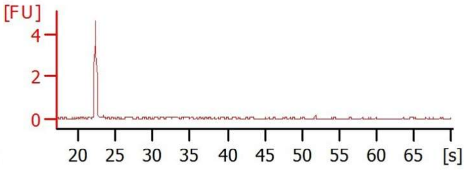

Ritu+Kit_Epidermis+Dermis_2

RIN:1

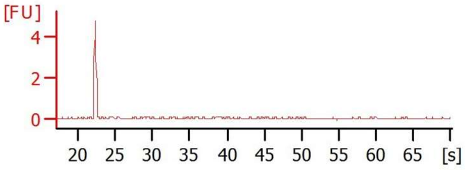

Ritu+Kit_Dermis

RIN:1

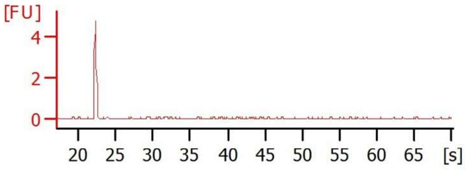

Ritu+Kit_Dermis_2

RIN:1

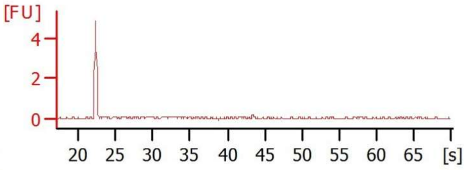

Assay Class:	Eukaryote Total RNA Nano	Created:	8/27/2021 1:27:30 PM
Data Path:	C:\...Eukaryote Total RNA Nano_DE13701055_2021-08-27_13-27-31.xad	Modified:	8/27/2021 1:51:20 PM

Electrophoresis File Run Summary (Chip Summary)

Sample Name	Sample Comment	Status	Result Label	Result Color
Ritu'sprotocol_Epidermis+dermis		✔	RIN: 2.50	
Ritu'sprotocol_Epidermis+dermis_2		✔	RIN: 2.40	
Ritu'sprotocol_dermis		✔	RIN:1	
Ritu'sprotocol_dermis_2		✔	RIN:1	
Kit_Epidermis+dermis		✔	RIN: 7.70	
Kit_Epidermis+dermis_2		✔	RIN: 7.40	
Kit_Dermis		✔	RIN:1	
Kit_Dermis_2		✔	RIN:1	
Ritu+Kit_Epidermis+Dermis		✔	RIN:1	
Ritu+Kit_Epidermis+Dermis_2		✔	RIN:1	
Ritu+Kit_Dermis		✔	RIN:1	
Ritu+Kit_Dermis_2		✔	RIN:1	
Ladder		✔	All Other Samples	

Chip Lot # **Reagent Kit Lot #**

Chip Comments :

Assay Class:	Eukaryote Total RNA Nano	Created:	8/27/2021 1:27:30 PM
Data Path:	C:\...Eukaryote Total RNA Nano_DE13701055_2021-08-27_13-27-31.xad	Modified:	8/27/2021 1:51:20 PM

Electrophoresis Assay Details

General Analysis Settings

Number of Available Sample and Ladder Wells (Max.) : 13
Minimum Visible Range [s] : 17
Maximum Visible Range [s] : 70
Start Analysis Time Range [s] : 19
End Analysis Time Range [s] : 69
Ladder Concentration [ng/µl] : 150
Lower Marker Concentration [ng/µl] : 0
Upper Marker Concentration [ng/µl] : 0
Used Lower Marker for Quantitation
Standard Curve Fit is Logarithmic
Show Data Aligned to Lower Marker

Integrator Settings

Integration Start Time [s] : 19
Integration End Time [s] : 69
Slope Threshold : 0.6
Height Threshold [FU] : 0.5
Area Threshold : 0.2
Width Threshold [s] : 0.5
Baseline Plateau [s] : 6

Filter Settings

Filter Width [s] : 0.5
Polynomial Order : 4

Ladder

Ladder Peak	Size
1	25
2	200
3	500
4	1000
5	2000
6	4000

Assay Class:	Eukaryote Total RNA Nano	Created:	8/27/2021 1:27:30 PM
Data Path:	C:\...Eukaryote Total RNA Nano_DE13701055_2021-08-27_13-27-31.xad	Modified:	8/27/2021 1:51:20 PM

Electropherogram Summary

Ladder

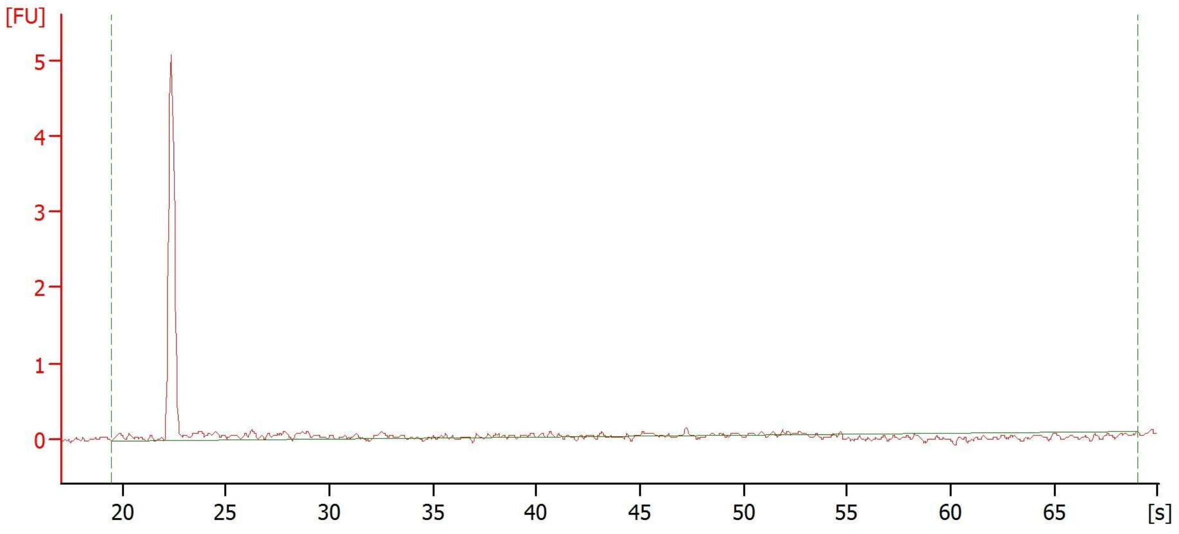

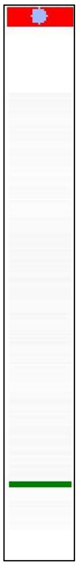

Overall Results for Ladder

RNA Area:	2.8	Result Flagging Color:	
RNA Concentration:	150 ng/µl	Result Flagging Label:	All Other Samples

Assay Class:	Eukaryote Total RNA Nano	Created:	8/27/2021 1:27:30 PM
Data Path:	C:\...Eukaryote Total RNA Nano_DE13701055_2021-08-27_13-27-31.xad	Modified:	8/27/2021 1:51:20 PM

Electropherogram Summary Continued ...

Ritu'sprotocol_Epidermis+dermis

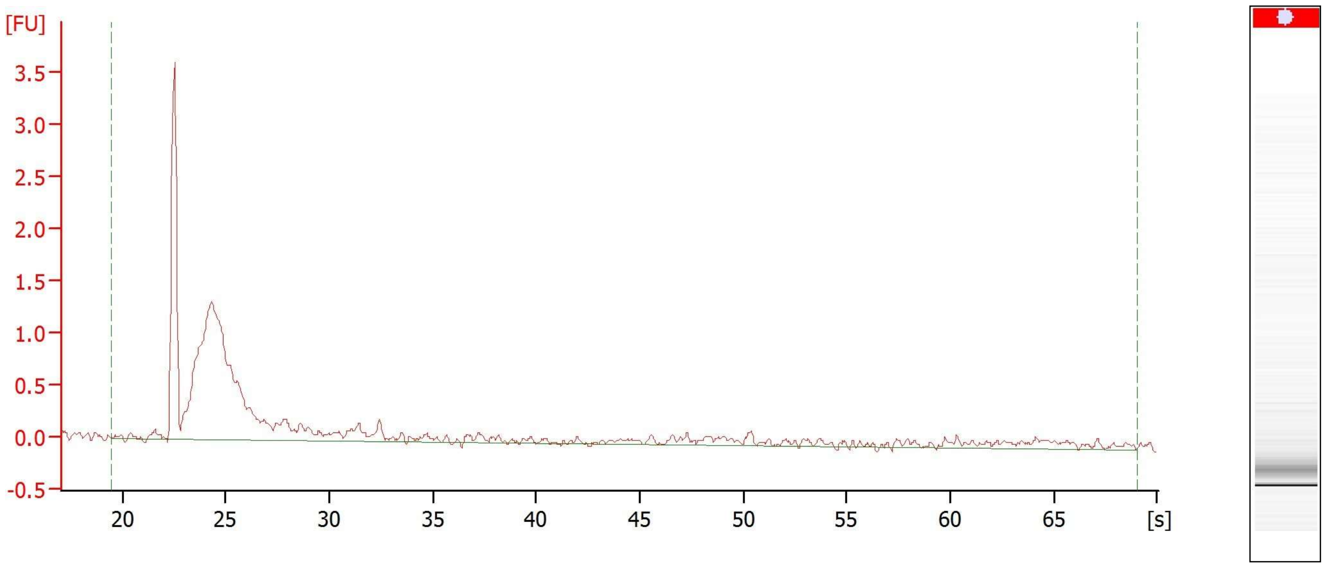

Overall Results for sample 1 : **Ritu'sprotocol_Epidermis+dermis**

RNA Area:	20.4	RNA Integrity Number (RIN):	2.5 (B.02.09)
RNA Concentration:	1,083 ng/µl	Result Flagging Color:	
rRNA Ratio [28s / 18s]:	0.0	Result Flagging Label:	RIN: 2.50

Electropherogram Summary Continued ...

Ritu'sprotocol_Epidermis+dermis_2

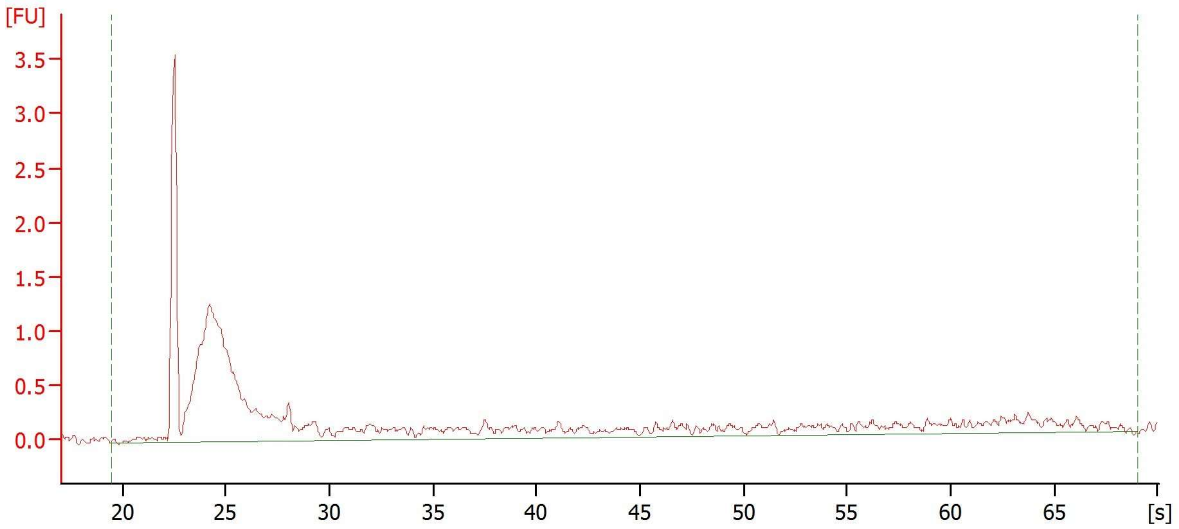

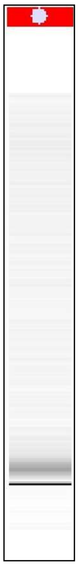

Overall Results for sample 2 : **Ritu'sprotocol_Epidermis+dermis_2**

RNA Area:	24.0	RNA Integrity Number (RIN):	2.4 (B.02.09)
RNA Concentration:	1,274 ng/µl	Result Flagging Color:	
rRNA Ratio [28s / 18s]:	0.0	Result Flagging Label:	RIN: 2.40

Electropherogram Summary Continued ...

Ritu'sprotocol_dermis

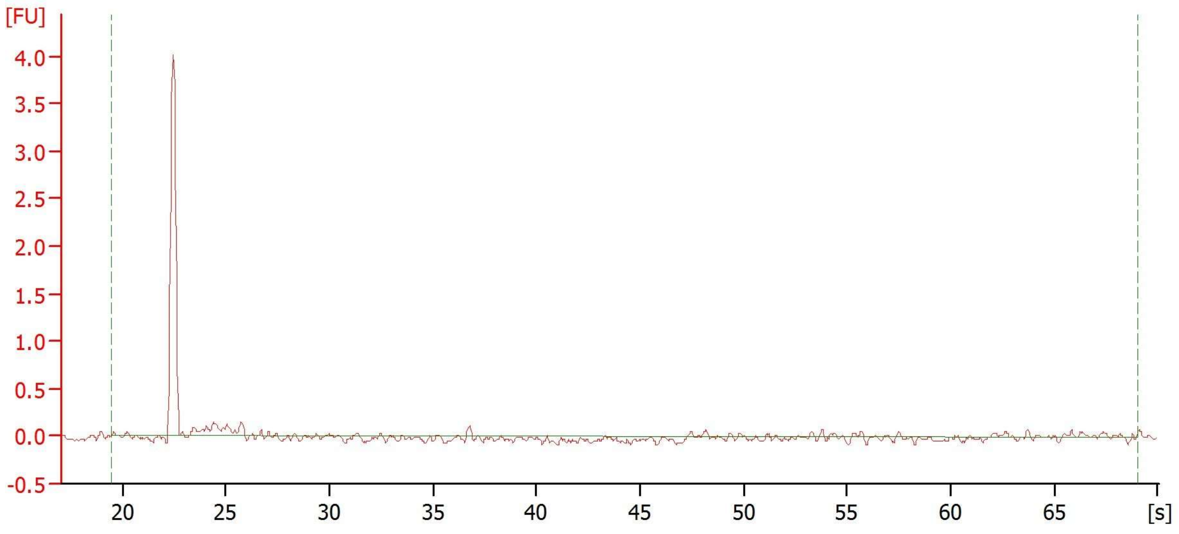

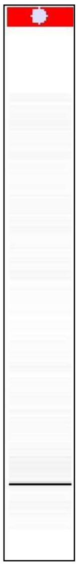

Overall Results for sample 3 : **<u>Ritu'sprotocol_dermis</u>**

RNA Area:	6.2	RNA Integrity Number (RIN):	1 (B.02.09)
RNA Concentration:	330 ng/µl	Result Flagging Color:	
rRNA Ratio [28s / 18s]:	0.0	Result Flagging Label:	RIN:1

Electropherogram Summary Continued ...

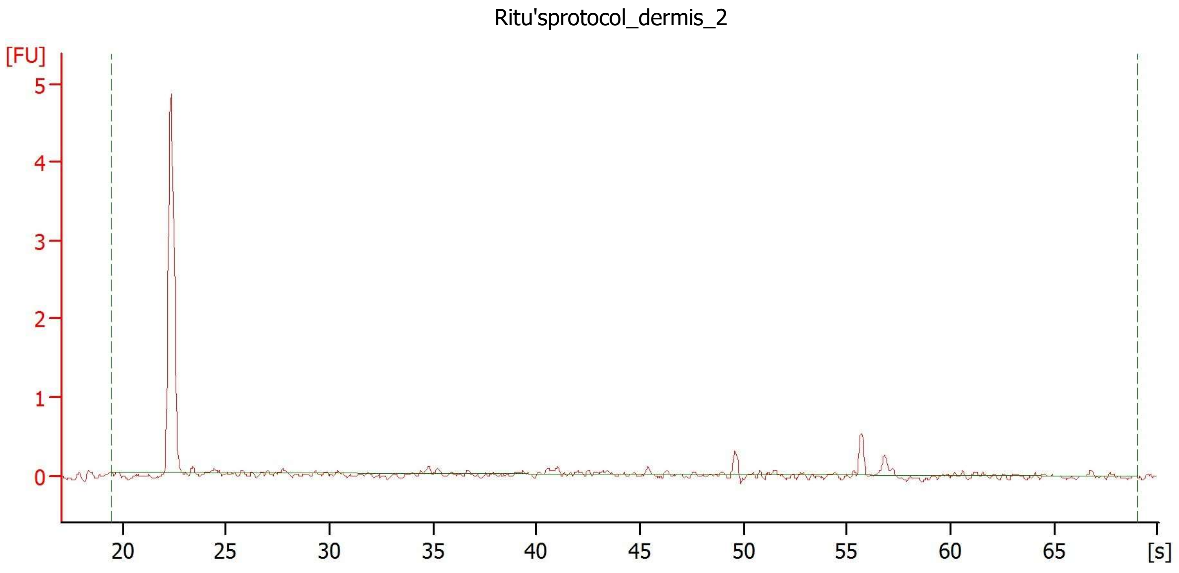

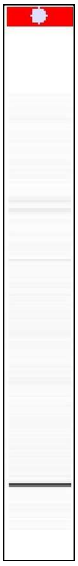

Overall Results for sample 4 : **Ritu'sprotocol_dermis_2**

RNA Area:	8.1	RNA Integrity Number (RIN):	1 (B.02.09)
RNA Concentration:	429 ng/µl	Result Flagging Color:	
rRNA Ratio [28s / 18s]:	0.0	Result Flagging Label:	RIN:1

Electropherogram Summary Continued ...

Kit_Epidermis+dermis

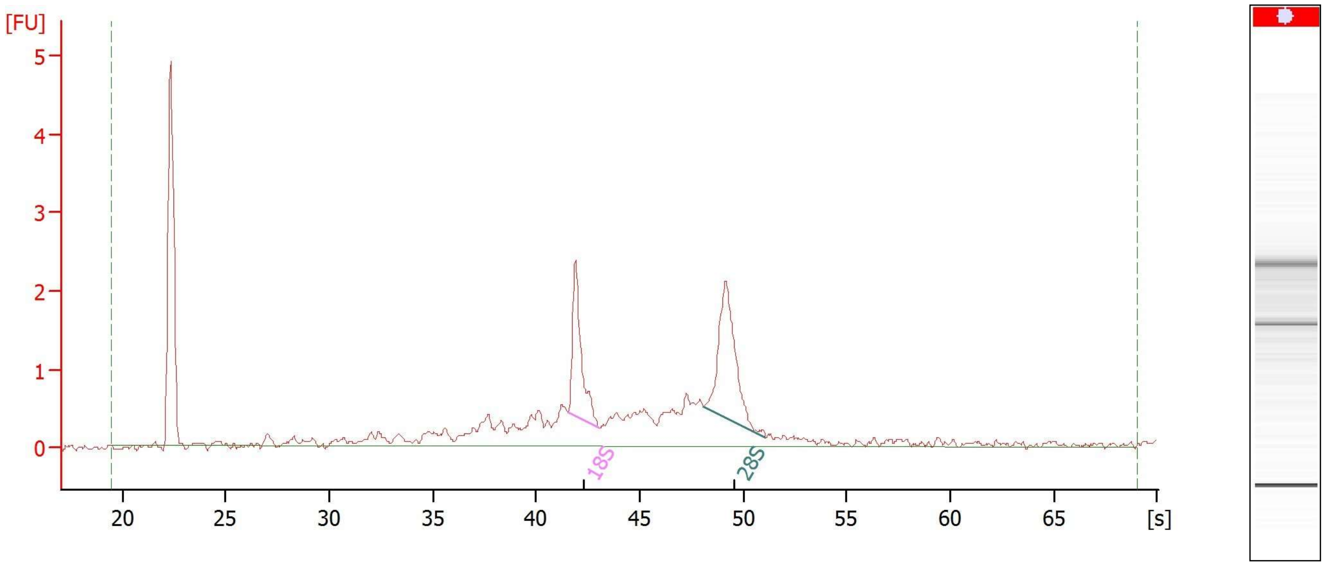

Overall Results for sample 5 : **Kit_Epidermis+dermis**

RNA Area:	28.2	RNA Integrity Number (RIN):	7.7 (B.02.09)
RNA Concentration:	1,501 ng/µl	Result Flagging Color:	
rRNA Ratio [28s / 18s]:	1.4	Result Flagging Label:	RIN: 7.70

Fragment table for sample 5 : **Kit_Epidermis+dermis**

Name	Start Size [nt]	End Size [nt]	Area	% of total Area
18S	0	0	2.4	8.4
28S	0	0	3.2	11.5

Assay Class: Eukaryote Total RNA Nano
Data Path: C:\...Eukaryote Total RNA Nano_DE13701055_2021-08-27_13-27-31.xad

Created: 8/27/2021 1:27:30 PM
Modified: 8/27/2021 1:51:20 PM

Electropherogram Summary Continued ...

Kit_Epidermis+dermis_2

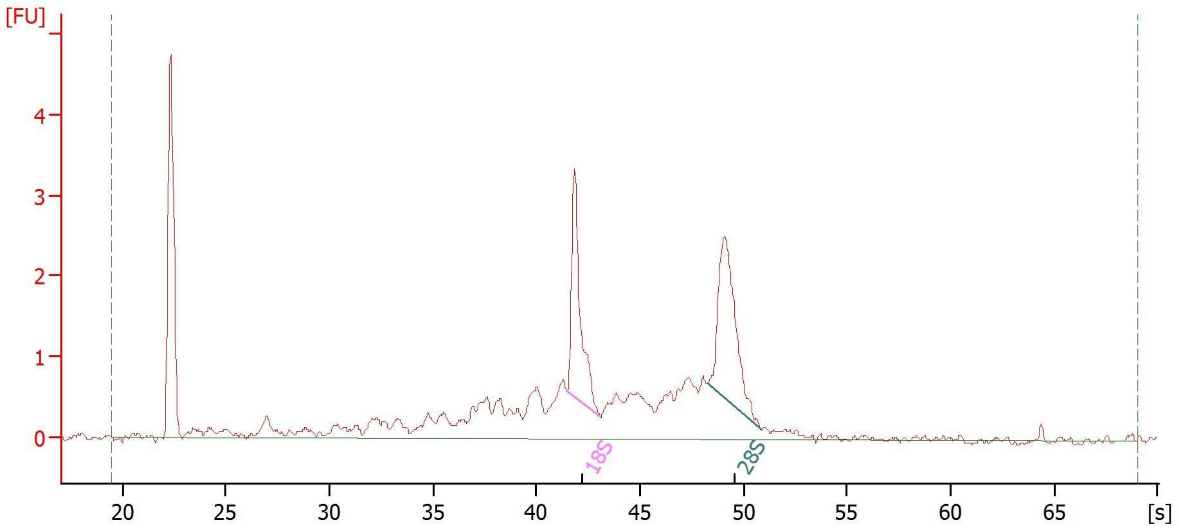

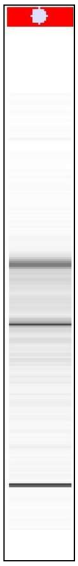

Overall Results for sample 6 : **Kit_Epidermis+dermis_2**

RNA Area:	35.2	RNA Integrity Number (RIN):	7.4 (B.02.09)
RNA Concentration:	1,870 ng/µl	Result Flagging Color:	
rRNA Ratio [28s / 18s]:	1.2	Result Flagging Label:	RIN: 7.40

Fragment table for sample 6 : **Kit_Epidermis+dermis_2**

Name	Start Size [nt]	End Size [nt]	Area	% of total Area
18S	0	0	3.4	9.6
28S	0	0	4.0	11.4

Assay Class:	Eukaryote Total RNA Nano	Created:	8/27/2021 1:27:30 PM
Data Path:	C:\...Eukaryote Total RNA Nano_DE13701055_2021-08-27_13-27-31.xad	Modified:	8/27/2021 1:51:20 PM

Electropherogram Summary Continued ...

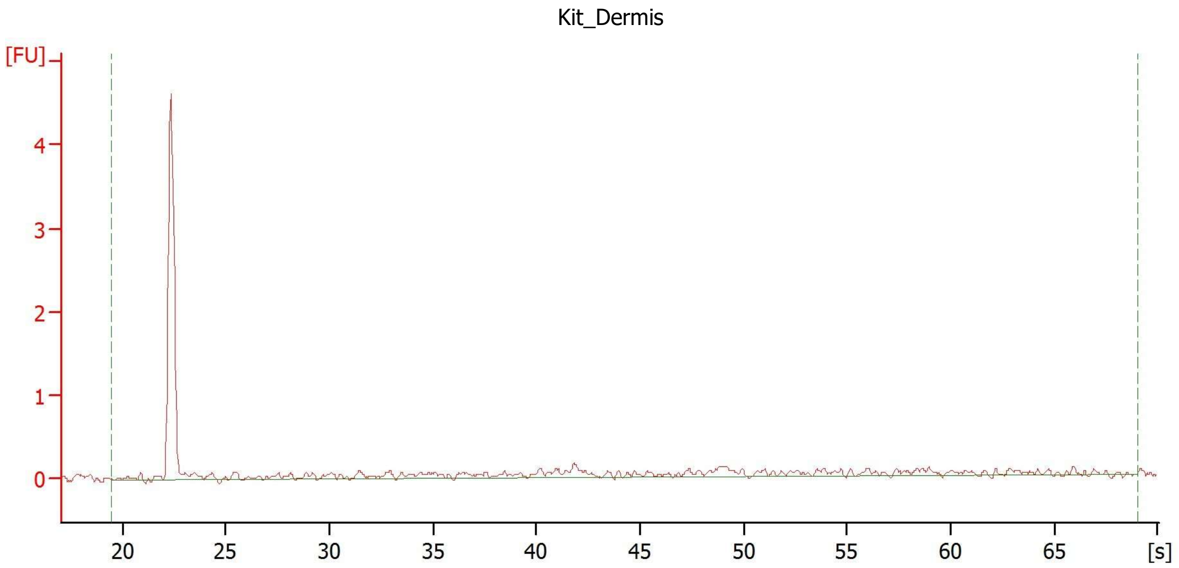

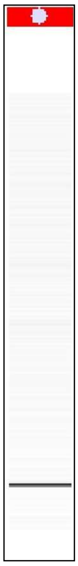

Overall Results for sample 7 : **Kit_Dermis**

RNA Area:	10.2	RNA Integrity Number (RIN):	1 (B.02.09)
RNA Concentration:	542 ng/µl	Result Flagging Color:	
rRNA Ratio [28s / 18s]:	0.0	Result Flagging Label:	RIN:1

Assay Class:	Eukaryote Total RNA Nano	Created:	8/27/2021 1:27:30 PM
Data Path:	C:\...Eukaryote Total RNA Nano_DE13701055_2021-08-27_13-27-31.xad	Modified:	8/27/2021 1:51:20 PM

Electropherogram Summary Continued ...

Kit_Dermis_2

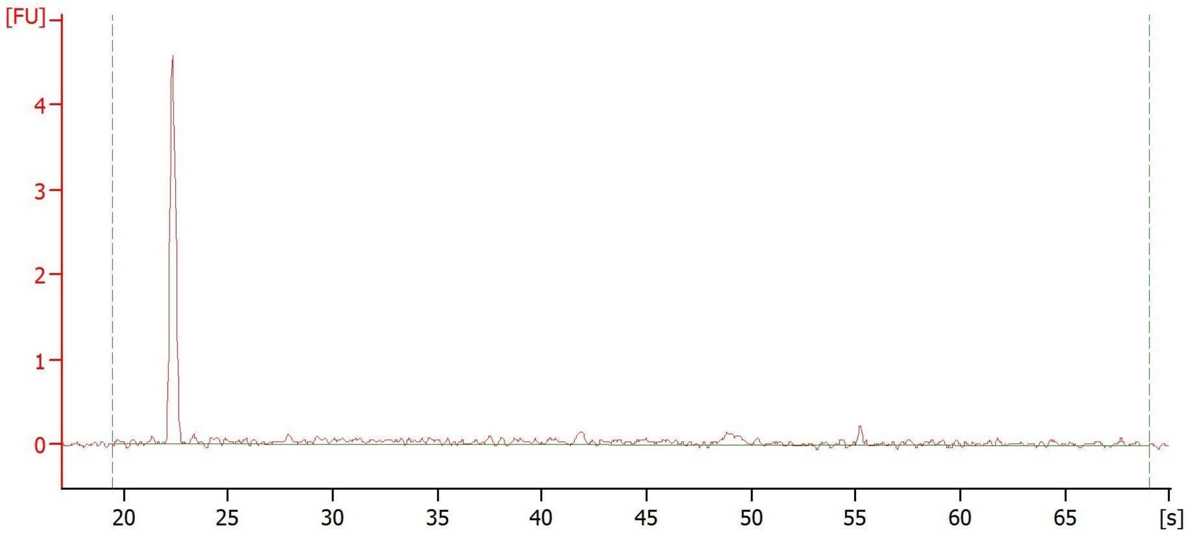

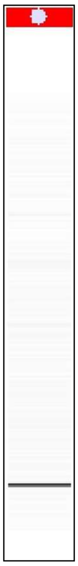

Overall Results for sample 8 : **Kit_Dermis_2**

RNA Area:	10.5	RNA Integrity Number (RIN):	1 (B.02.09)
RNA Concentration:	557 ng/µl	Result Flagging Color:	
rRNA Ratio [28s / 18s]:	0.0	Result Flagging Label:	RIN:1

Assay Class: Eukaryote Total RNA Nano
Data Path: C:\...Eukaryote Total RNA Nano_DE13701055_2021-08-27_13-27-31.xad

Created: 8/27/2021 1:27:30 PM
Modified: 8/27/2021 1:51:20 PM

Electropherogram Summary Continued ...

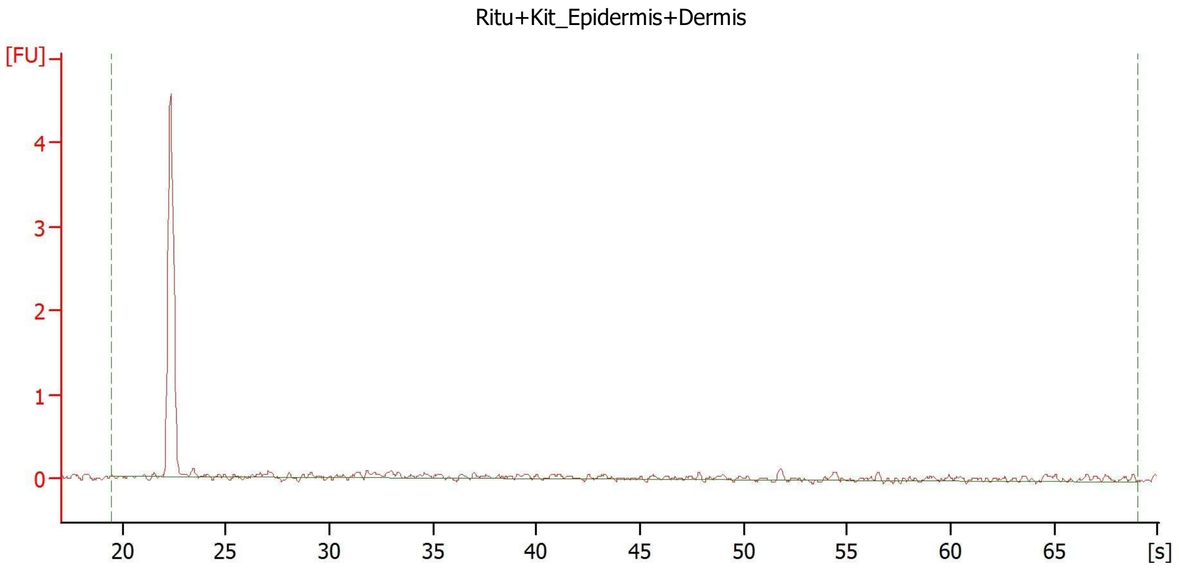

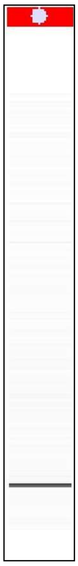

Overall Results for sample 9 : **Ritu+Kit_Epidermis+Dermis**

RNA Area:	8.2	RNA Integrity Number (RIN):	1 (B.02.09)
RNA Concentration:	437 ng/µl	Result Flagging Color:	
rRNA Ratio [28s / 18s]:	0.0	Result Flagging Label:	RIN:1

Electropherogram Summary Continued ...

Ritu+Kit_Epidermis+Dermis_2

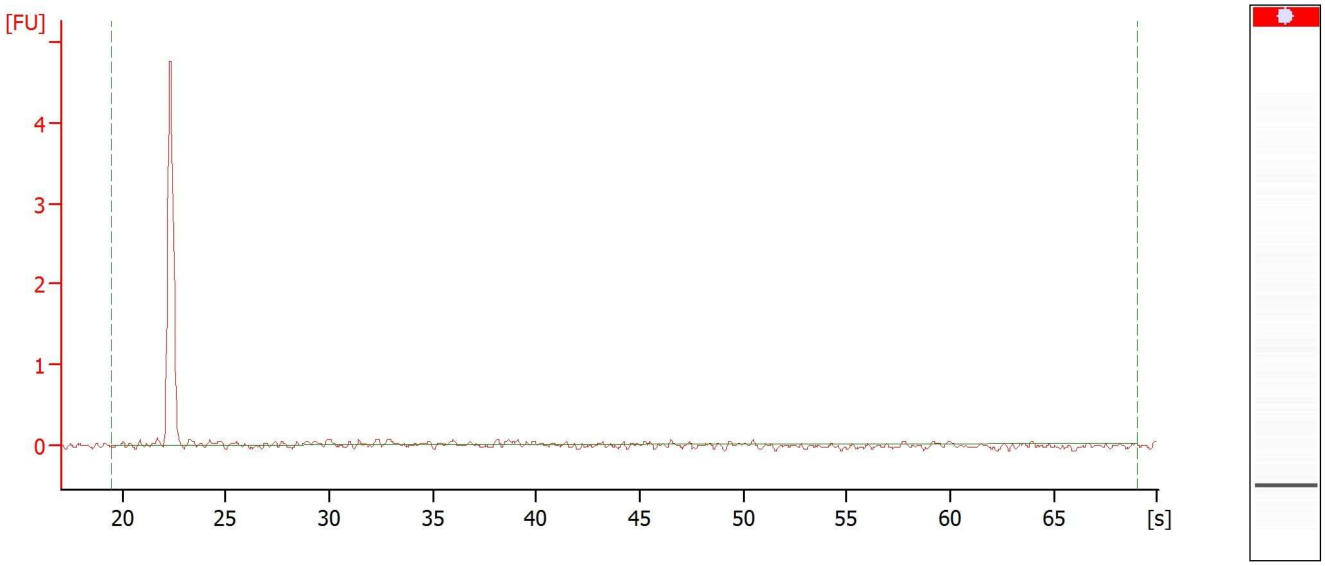

Overall Results for sample 10 : **Ritu+Kit_Epidermis+Dermis_2**

RNA Area:	8.3	RNA Integrity Number (RIN):	1 (B.02.09)
RNA Concentration:	440 ng/µl	Result Flagging Color:	
rRNA Ratio [28s / 18s]:	0.0	Result Flagging Label:	RIN:1

Assay Class:	Eukaryote Total RNA Nano	Created:	8/27/2021 1:27:30 PM
Data Path:	C:\...Eukaryote Total RNA Nano_DE13701055_2021-08-27_13-27-31.xad	Modified:	8/27/2021 1:51:20 PM

Electropherogram Summary Continued ...

Ritu+Kit_Dermis

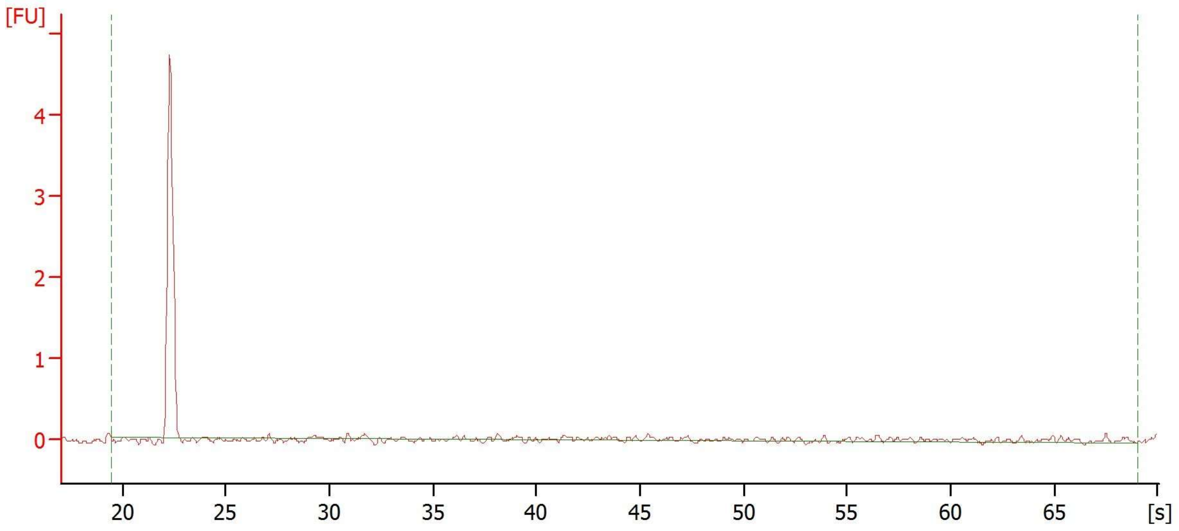

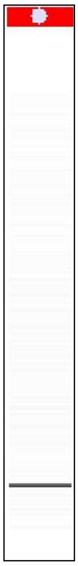

Overall Results for sample 11 : **Ritu+Kit_Dermis**

RNA Area:	7.7	RNA Integrity Number (RIN):	1 (B.02.09)
RNA Concentration:	411 ng/µl	Result Flagging Color:	
rRNA Ratio [28s / 18s]:	0.0	Result Flagging Label:	RIN:1

Assay Class: Eukaryote Total RNA Nano
Data Path: C:\...Eukaryote Total RNA Nano_DE13701055_2021-08-27_13-27-31.xad

Created: 8/27/2021 1:27:30 PM
Modified: 8/27/2021 1:51:20 PM

Electropherogram Summary Continued ...

Ritu+Kit_Dermis_2

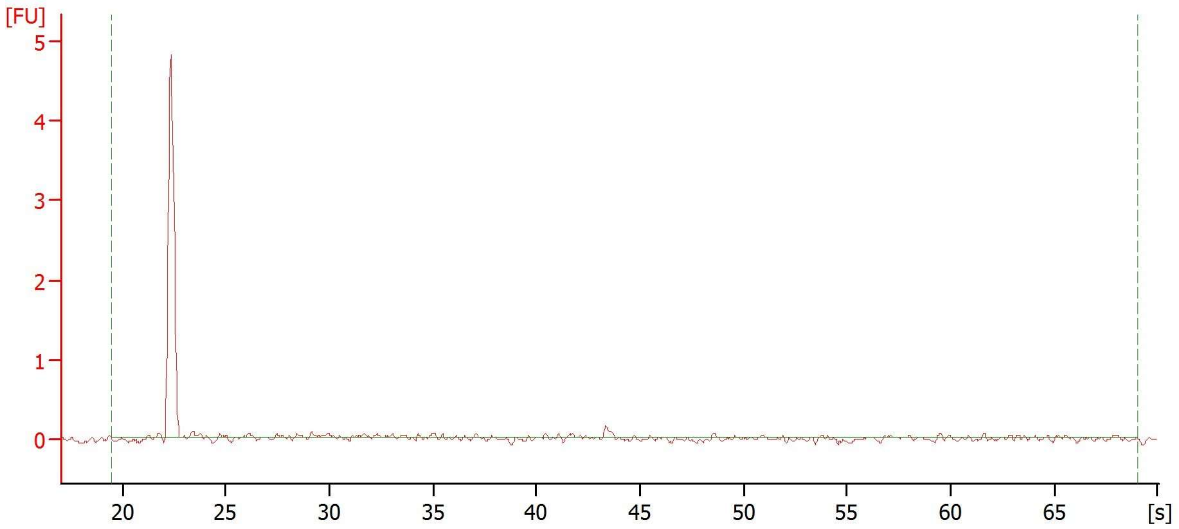

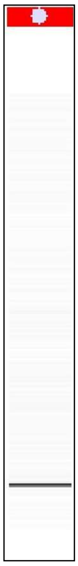

Overall Results for sample 12 : **Ritu+Kit_Dermis_2**

RNA Area:	7.7	RNA Integrity Number (RIN):	1 (B.02.09)
RNA Concentration:	410 ng/µl	Result Flagging Color:	
rRNA Ratio [28s / 18s]:	0.0	Result Flagging Label:	RIN:1

Assay Class:	Eukaryote Total RNA Nano	Created:	8/27/2021 1:27:30 PM
Data Path:	C:\...Eukaryote Total RNA Nano_DE13701055_2021-08-27_13-27-31.xad	Modified:	8/27/2021 1:51:20 PM

Gel Image

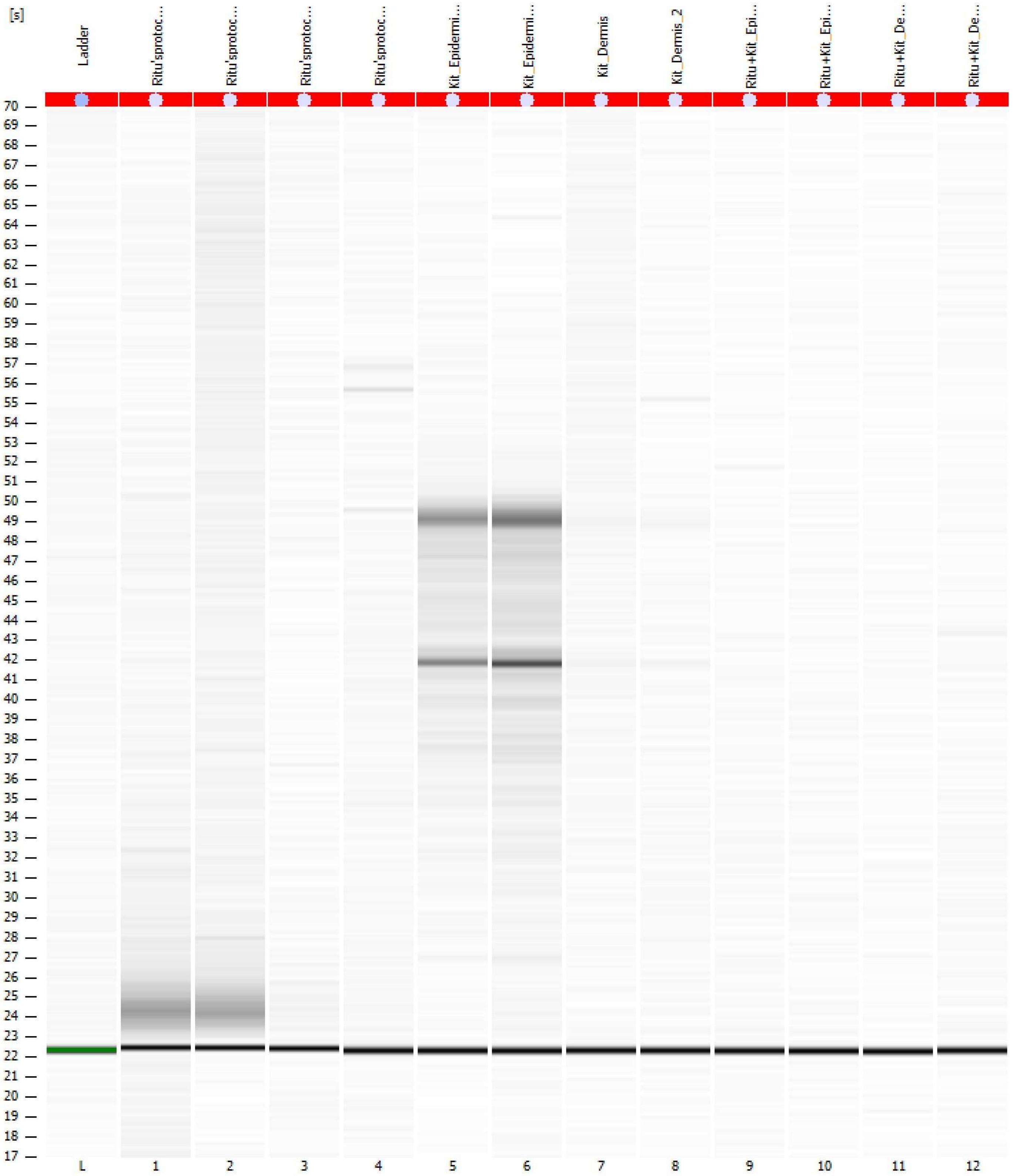

Assay Class:	Eukaryote Total RNA Nano	Created:	8/27/2021 1:27:30 PM
Data Path:	C:\...Eukaryote Total RNA Nano_DE13701055_2021-08-27_13-27-31.xad	Modified:	8/27/2021 1:51:20 PM

Run Logbook

Description	Number	Source	Category	Sub Category	Time	Time Zone	User	Host
Run ended on port 1 (Number of wells acquired: 13)		Instrument	Run		8/27/2021 1:51:18 PM	(GMT +02:00) W. Europe Standard Time	kigene	K1-NP02-031L
Run started on port 1 (File: C:\Program Files (x86)\Agilent\2100 bioanalyzer\2100 expert\Data\2021-08-27\2100 expert_Eukaryote Total RNA Nano_DE13701055_2021-08-27_13-27-31.xad)		Instrument	Run		8/27/2021 1:27:36 PM	(GMT +02:00) W. Europe Standard Time	kigene	K1-NP02-031L
Product Number : G2938B		Instrument	Run		8/27/2021 1:27:36 PM	(GMT +02:00) W. Europe Standard Time	kigene	K1-NP02-031L
Name : com2		Instrument	Run		8/27/2021 1:27:36 PM	(GMT +02:00) W. Europe Standard Time	kigene	K1-NP02-031L
Vendor : Agilent Technologies		Instrument	Run		8/27/2021 1:27:36 PM	(GMT +02:00) W. Europe Standard Time	kigene	K1-NP02-031L
Serial# : DE13701055		Instrument	Run		8/27/2021 1:27:36 PM	(GMT +02:00) W. Europe Standard Time	kigene	K1-NP02-031L
Firmware : C.01.069		Instrument	Run		8/27/2021 1:27:36 PM	(GMT +02:00) W. Europe Standard Time	kigene	K1-NP02-031L
Cartridge : Electrode		Instrument	Run		8/27/2021 1:27:36 PM	(GMT +02:00) W. Europe Standard Time	kigene	K1-NP02-031L

Appendix Figure S1 – DD patient RNA quality assessment. Bioanalyzer assessment of RNA derived from DD patient samples, including the demonstration that negligible RNA is isolated from dermis samples.

	Cell	obs.x	obs.y	obs.tot	mean.x	mean.y	mean.diff	var.x	var.y	stderr	df	statistic	pvalue	conf.low	conf.high	alternative	mean.null	conf.level
1	Monocytes	8	12	20	6.40E-05	0.00029	-0.00023	1.41E-08	1.01E-06	0.000293	11.45705	-0.77108	0.456269	-0.00087	0.000416	two.sided	0	0.95
2	CD8+ T-cells	8	12	20	0.002357	0.00243	-7.32E-05	5.52E-06	5.25E-06	0.001062	14.88703	-0.06891	0.945978	-0.00234	0.002191	two.sided	0	0.95
3	NK cells	8	12	20	0.000248	0.000179	6.98E-05	2.13E-07	3.09E-07	0.000229	16.97674	0.304924	0.76413	-0.00041	0.000553	two.sided	0	0.95
4	Macrophages	8	12	20	0.000231	0.00054	-0.00031	2.22E-07	4.57E-07	0.000257	17.92085	-1.2027	0.244742	-0.00085	0.000231	two.sided	0	0.95
5	Endothelial cells	8	12	20	0.041182	0.019399	0.021783	0.000646	0.000272	0.010166	10.93293	2.142662	0.055497	-0.00061	0.044175	two.sided	0	0.95
6	DC	8	12	20	0.000664	0.002663	-0.002	8.14E-07	3.62E-05	0.001767	11.73268	-1.13157	0.280416	-0.00586	0.00186	two.sided	0	0.95
7	Neutrophils	8	12	20	0.000636	0.001371	-0.00074	1.21E-06	1.69E-06	0.000541	16.8263	-1.35984	0.191825	-0.00188	0.000407	two.sided	0	0.95
8	CD4+ naive T-cells	8	12	20	0.000608	0.000818	-0.00021	3.70E-07	6.97E-07	0.000323	17.77666	-0.65221	0.522608	-0.00089	0.000469	two.sided	0	0.95
9	Smooth muscle	8	12	20	0.006698	0.010397	-0.0037	6.74E-05	5.18E-05	0.003569	13.71623	-1.0367	0.31781	-0.01137	0.003969	two.sided	0	0.95
10	Fibroblasts	8	12	20	1.36E-19	2.20E-19	-8.41E-20	3.09E-38	7.96E-38	1.02E-19	17.96677	-0.82082	0.422505	-2.99E-19	1.31E-19	two.sided	0	0.95
11	Keratinocytes	8	12	20	0.000334	0.000819	-0.00048	2.14E-07	3.39E-07	0.000234	17.31082	-2.06808	0.053912	-0.00098	9.11E-06	two.sided	0	0.95
12	B-cells	8	12	20	0.001092	0.000608	0.000483	1.11E-06	3.12E-07	0.000407	9.646051	1.188779	0.262968	-0.00043	0.001394	two.sided	0	0.95
13	CD8+ Tem	8	12	20	0.000155	0.000665	-0.00051	1.05E-07	8.19E-07	0.000285	14.7781	-1.78928	0.094081	-0.00112	9.84E-05	two.sided	0	0.95
14	Tregs	8	12	20	0.003054	0.000927	0.002127	9.14E-06	1.94E-06	0.001142	9.00991	1.862459	0.095403	-0.00046	0.004709	two.sided	0	0.95
15	Plasma cells	8	12	20	0.00075	0.00024	0.00051	5.74E-	1.79E-	0.00029	9.93761	1.75601	0.10979	-0.00	0.00117	two.side	0	0.95

					9	2	7	07	07	4	7	9	8	014	4	d		
16	CD4+ Tcm	8	12	20	0.000337	0.000263	7.41E-05	4.45E-07	3.77E-07	0.000295	14.25186	0.251031	0.805373	-0.00056	0.000706	two.sided	0	0.95
17	mv Endothelial cells	8	12	20	0.003382	0.002397	0.000985	4.70E-06	4.67E-06	0.000988	15.1245	0.99734	0.334289	-0.00112	0.003089	two.sided	0	0.95
18	CD4+ Tem	8	12	20	0.000232	0.002672	-0.00244	4.31E-07	9.84E-06	0.000935	12.40891	-2.60982	0.022288	-0.00447	-0.00041	two.sided	0	0.95
19	Memory B-cells	8	12	20	0.000105	0.000396	-0.00029	8.82E-08	1.69E-07	0.000158	17.80511	-1.83928	0.08261	-0.00062	4.17E-05	two.sided	0	0.95
20	CD8+ Tcm	8	12	20	0.000416	1.21E-05	0.000404	6.11E-07	1.77E-09	0.000277	7.026992	1.459425	0.187657	-0.00025	0.001057	two.sided	0	0.95
21	naive B-cells	8	12	20	0.000828	0.000163	0.000666	3.15E-07	4.19E-08	0.000207	8.257348	3.216467	0.0118	0.000191	0.001141	two.sided	0	0.95
22	Eosinophils	8	12	20	0.001366	0.001642	-0.00028	2.59E-06	4.51E-06	0.000837	17.59188	-0.32934	0.74579	-0.00204	0.001485	two.sided	0	0.95
23	ly Endothelial cells	8	12	20	0.018379	0.007392	0.010987	0.000179	6.92E-05	0.005303	10.62856	2.07177	0.063457	-0.00074	0.022708	two.sided	0	0.95
24	MSC	8	12	20	0.002122	0.002256	-0.00013	8.89E-06	1.77E-05	0.001609	17.87835	-0.08303	0.934751	-0.00352	0.003248	two.sided	0	0.95
25	Macrophages M2	8	12	20	0.000467	0.000851	-0.00038	3.93E-07	6.52E-07	0.000322	17.45496	-1.19283	0.248898	-0.00106	0.000294	two.sided	0	0.95
26	aDC	8	12	20	0.005486	0.002759	0.002727	1.58E-05	2.12E-05	0.001935	16.64181	1.409436	0.17712	-0.00136	0.006815	two.sided	0	0.95
27	Preadipocytes	8	12	20	0.000276	6.69E-19	0.000276	6.07E-07	3.72E-37	0.000276	7	1	0.350617	-0.00038	0.000927	two.sided	0	0.95
28	Melanocytes	8	12	20	0.008625	0.009766	-0.00114	3.53E-05	6.82E-05	0.003179	17.82492	-0.35891	0.72388	-0.00782	0.005542	two.sided	0	0.95
29	CD4+ memory T-cells	8	12	20	0.0026	0.00153	0.00107	2.89E-06	2.06E-06	0.00073	13.31646	1.465493	0.165997	-0.0005	0.002643	two.sided	0	0.95
30	Basophils	8	12	20	0.010076	0.006702	0.003373	0.000167	7.03E-05	0.005174	10.92492	0.652007	0.527876	-0.00802	0.01477	two.sided	0	0.95
3	cDC	8	12	20	0.00	0.00	-	4.32	3.77	0.00	14.4	-	0.28	-	0.00	two.	0	0.95

1					1319	3439	0.00212	E-06	E-05	1918	3904	1.10534	7089	0.00622	1982	sided		
32	Pericytes	8	12	20	0.01228	0.010938	0.001342	5.92E-05	0.0001055	0.004021	17.64423	0.333668	0.742563	-0.00712	0.009801	two.sided	0	0.95
33	Class-switched memory B-cells	8	12	20	0.001628	0.002661	-0.00103	3.49E-06	1.73E-05	0.001372	16.31089	-0.75288	0.462253	-0.00394	0.001871	two.sided	0	0.95
34	Mast cells	8	12	20	0.000454	0.000493	-3.87E-05	1.56E-07	1.54E-07	0.00018	15.10418	-0.21554	0.832232	-0.00042	0.000344	two.sided	0	0.95
35	Th2 cells	8	12	20	0.001557	0.002117	-0.00056	1.29E-05	5.74E-06	0.001446	11.14855	-0.38753	0.705663	-0.00374	0.002617	two.sided	0	0.95
36	Th1 cells	8	12	20	0.000572	0.004977	-0.00441	1.22E-06	2.09E-05	0.001377	12.85456	-3.19975	0.00706	-0.00738	-0.00143	two.sided	0	0.95
37	CD8+ naive T-cells	8	12	20	0.001125	0.000131	0.000994	1.35E-06	8.29E-08	0.000419	7.577493	2.373029	0.046723	1.86E-05	0.001969	two.sided	0	0.95
38	NKT	8	12	20	0.000899	0.002131	-0.00123	1.36E-06	2.08E-05	0.00138	13.0715	-0.89237	0.388329	-0.00421	0.001748	two.sided	0	0.95

Appendix Figure S2 – Cell type deconvolution. Deconvolution analysis of cell type differences within DD patient-derived RNAseq analysis

Gene	Primer	Sequence
CHOP	Forward	AAGGCACTGAGCGTATCATGT
	Reverse	TGAAGATACACTTCCTTCTTGAACA
ATF6B	Forward	GAGTCATCGCGTCTCTCCAC
	Reverse	GGCCTCAGAGTTGACGGAAG
ERO1	Forward	GCCCGTTTTATGCTTGATGT
	Reverse	AACTGGGTATGGTGGCAGAC
ERN1	Forward	AGAGAAGCAGCAGACTTTGTC
	Reverse	GTTTTGGTGTCGTACATGGTGA
GRP78	Forward	GGATCATCAACGAGCCTACG
	Reverse	CACCCAGGTCAAACACCAG
sXBP1	Forward	CTGAGTCCGAATCAGGTGCAG
	Reverse	ATCCATGGGGAGATGTTCTGG
usXBP1	Forward	CAGCACTCAGACTACGTGCA
	Reverse	ATCCATGGGGAGATGTTCTGG
DSG1	Forward	TCCATAGTTGATCGAGAGGTCAC
	Reverse	CTGCGTCAGTAGCATTGAGTATC
DSG2	Forward	AAAACCTTTAATTTTGCCATTGA
	Reverse	GGGAGCCTTCAAGATCCCTA
DSG3	Forward	TGATCTGTCCCATTTCCAGTGT
	Reverse	TCATATTCGACGGGAGCAAGGA
E-CAD	Forward	ACAGCCCCGCCTTATGATT
	Reverse	TCGGAACCGCTTCCTTCA
DSP	Forward	CCAACTTGTCCTCAATCAGCATCCAG
	Reverse	GCTTCAGTAGACTGCGCCTCTTCAAA
DSC3	Forward	CACTTACTCGGAGTGGCACAGT
	Reverse	AGCTGGAGATCCTCTTCCCTCA
CLDN4	Forward	CTCCATGGGGCTACAGGTAA
	Reverse	AGCAGCGAGTCGTACACCTT
OCLN	Forward	TGCATGTTCGACCAATGC

Appendix Figure S3 – List of primers. List of primers used for qPCR analysis.
