# Supplementary figures and images for: Dantrolene corrects cellular disease features of Darier disease and may be a novel treatment (part 1 of 2)
Source: EMBO Mol Med. 2024 Jul 26;16(9):1986–2001. doi: 10.1038/s44321-024-00104-3 (PMC11392931; doi:10.1038/s44321-024-00104-3)

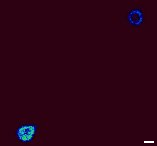

Supplement: Supplementary file 4 — Source data (part 2) Fig. 1 [file 44321_2024_104_MOESM4_ESM.zip › 1H/Baseline DDK.tif]

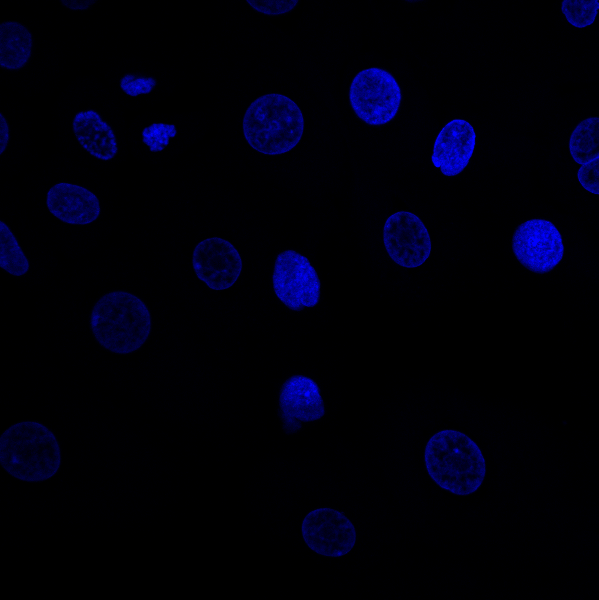

Supplement: Supplementary file 5 — Source data Fig. 2 [file 44321_2024_104_MOESM5_ESM.zip › Figure 2/2B/B-catenin Dl/DDK Dl B-cat DAPI.tif]

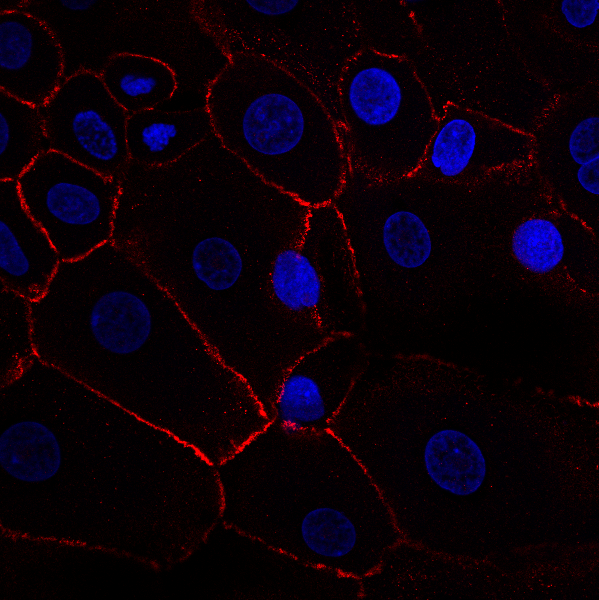

Supplement: Supplementary file 5 — Source data Fig. 2 [file 44321_2024_104_MOESM5_ESM.zip › Figure 2/2B/B-catenin Dl/DDK Dl B-cat Merge.tif]

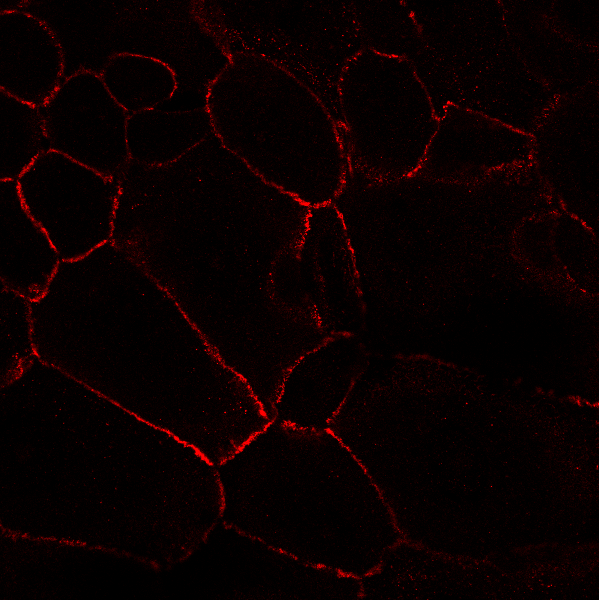

Supplement: Supplementary file 5 — Source data Fig. 2 [file 44321_2024_104_MOESM5_ESM.zip › Figure 2/2B/B-catenin Dl/DDK Dl B-cat.tif]

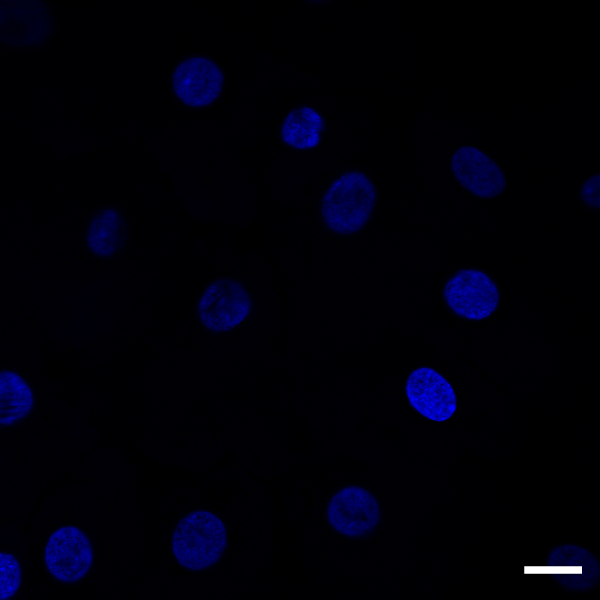

Supplement: Supplementary file 5 — Source data Fig. 2 [file 44321_2024_104_MOESM5_ESM.zip › Figure 2/2B/B-catenin Veh/DDK Veh B-cat DAPI.tif]

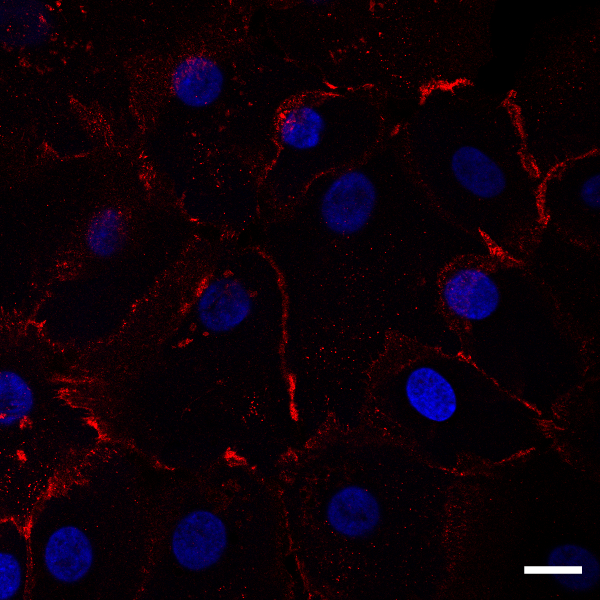

Supplement: Supplementary file 5 — Source data Fig. 2 [file 44321_2024_104_MOESM5_ESM.zip › Figure 2/2B/B-catenin Veh/DDK Veh B-cat Merge.tif]

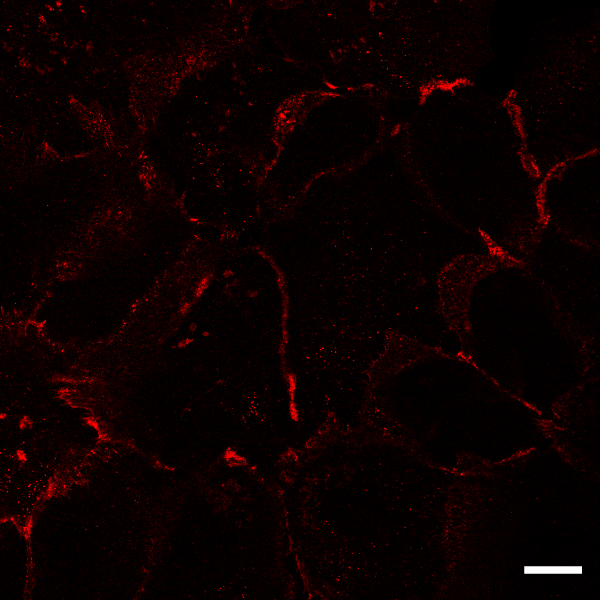

Supplement: Supplementary file 5 — Source data Fig. 2 [file 44321_2024_104_MOESM5_ESM.zip › Figure 2/2B/B-catenin Veh/DDK Veh B-cat.tif]

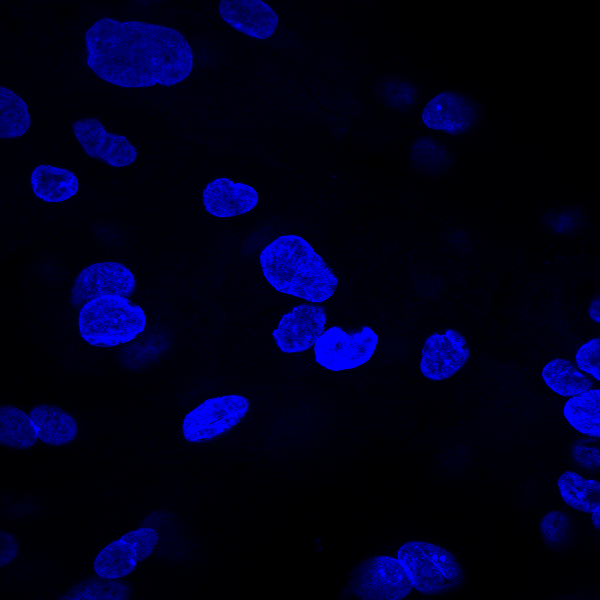

Supplement: Supplementary file 5 — Source data Fig. 2 [file 44321_2024_104_MOESM5_ESM.zip › Figure 2/2B/DSG1 Dl/DDK Dl DSG1 DAPI.tif]

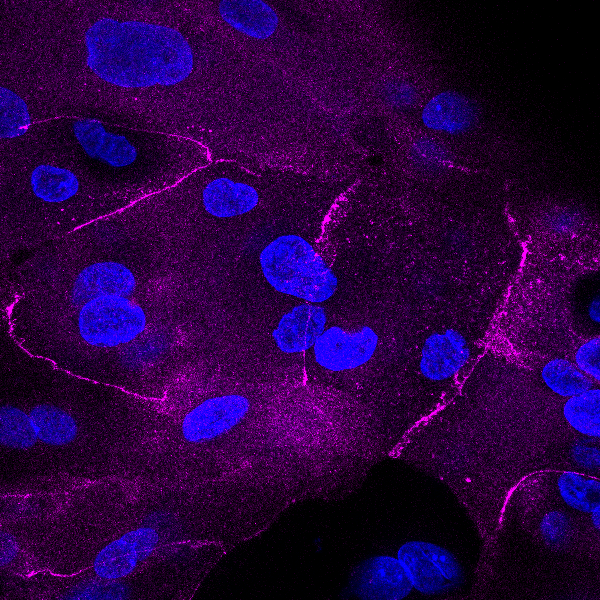

Supplement: Supplementary file 5 — Source data Fig. 2 [file 44321_2024_104_MOESM5_ESM.zip › Figure 2/2B/DSG1 Dl/DDK Dl DSG1 Merge.tif]

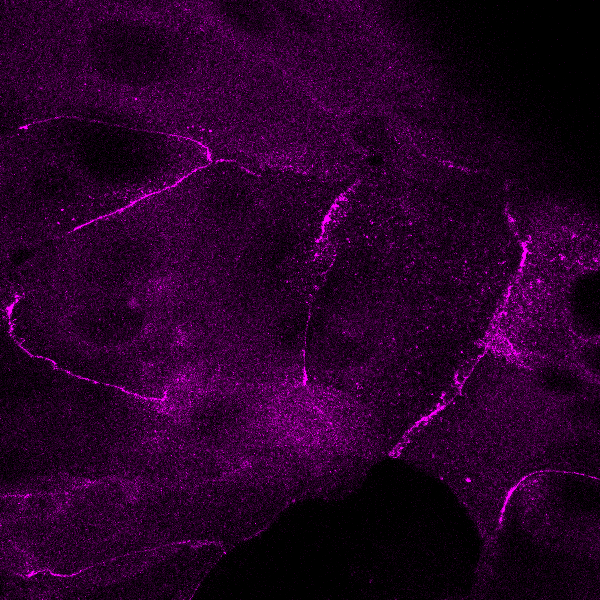

Supplement: Supplementary file 5 — Source data Fig. 2 [file 44321_2024_104_MOESM5_ESM.zip › Figure 2/2B/DSG1 Dl/DDK Dl DSG1.tif]

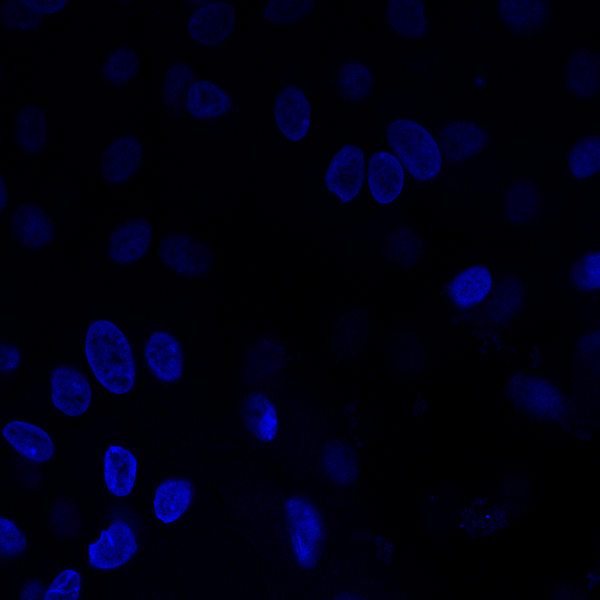

Supplement: Supplementary file 5 — Source data Fig. 2 [file 44321_2024_104_MOESM5_ESM.zip › Figure 2/2B/DSG1 Veh/DDK Veh DSG1 DAPI.tif]

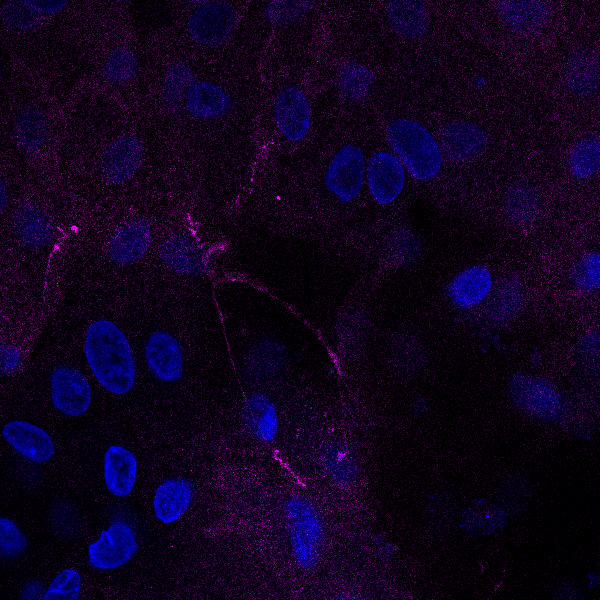

Supplement: Supplementary file 5 — Source data Fig. 2 [file 44321_2024_104_MOESM5_ESM.zip › Figure 2/2B/DSG1 Veh/DDK Veh DSG1 Merge.tif]

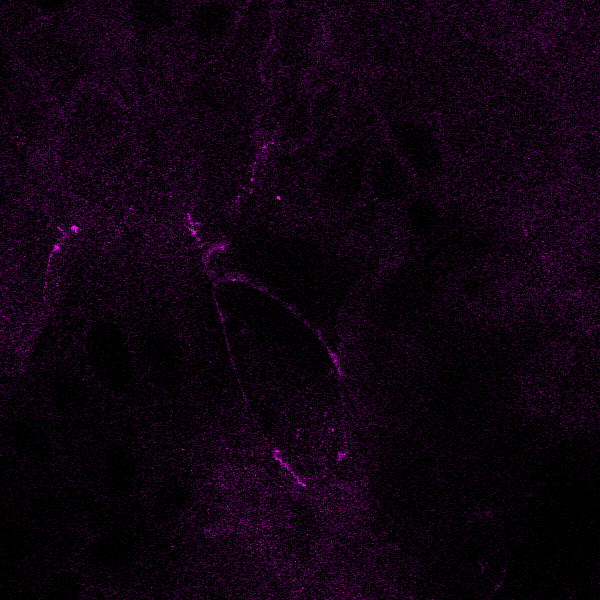

Supplement: Supplementary file 5 — Source data Fig. 2 [file 44321_2024_104_MOESM5_ESM.zip › Figure 2/2B/DSG1 Veh/DDK Veh DSG1.tif]

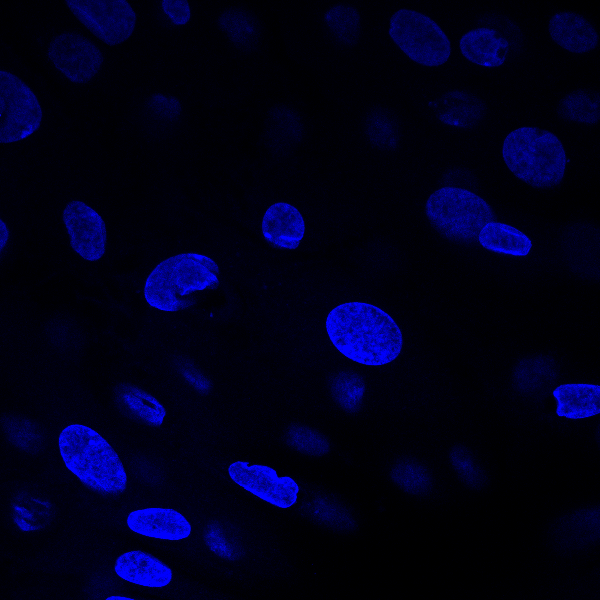

Supplement: Supplementary file 5 — Source data Fig. 2 [file 44321_2024_104_MOESM5_ESM.zip › Figure 2/2B/OCLN Dl/DDK Dl OCLN DAPI.tif]

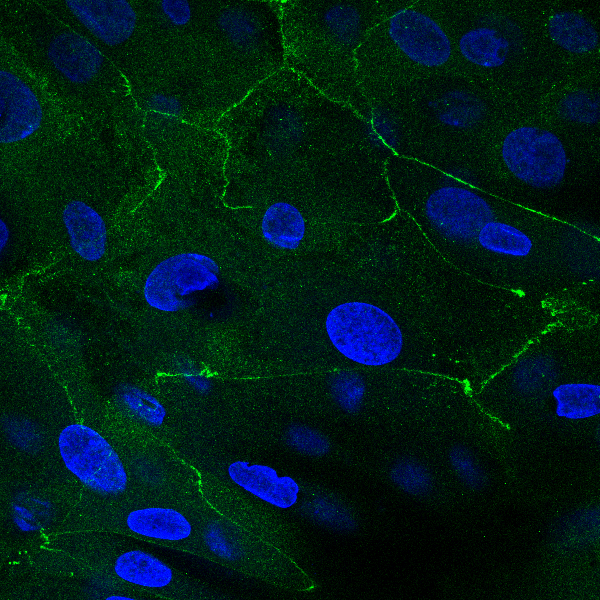

Supplement: Supplementary file 5 — Source data Fig. 2 [file 44321_2024_104_MOESM5_ESM.zip › Figure 2/2B/OCLN Dl/DDK Dl OCLN Merge.tif]

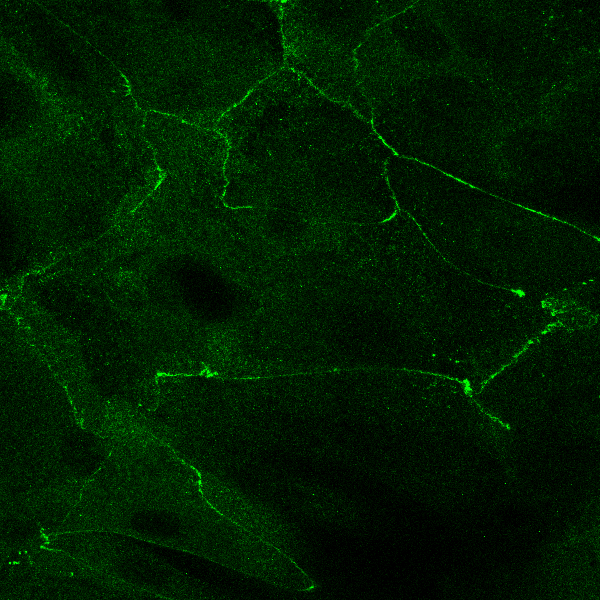

Supplement: Supplementary file 5 — Source data Fig. 2 [file 44321_2024_104_MOESM5_ESM.zip › Figure 2/2B/OCLN Dl/DDK Dl OCLN.tif]

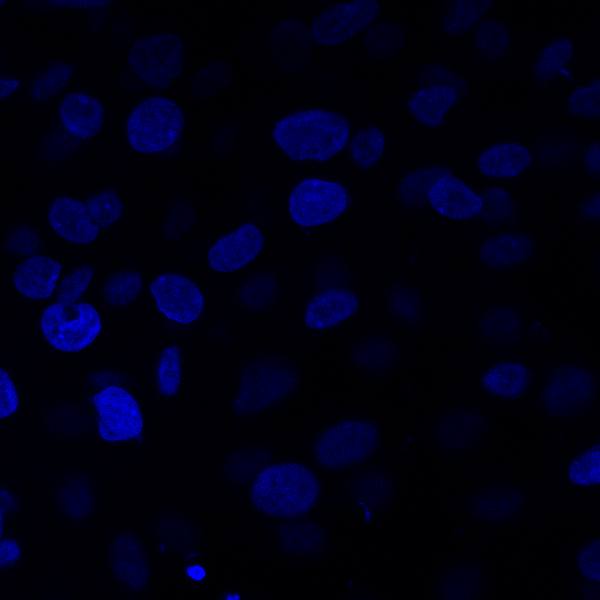

Supplement: Supplementary file 5 — Source data Fig. 2 [file 44321_2024_104_MOESM5_ESM.zip › Figure 2/2B/OCLN Veh/DDK Veh OCLN DAPI.tif]

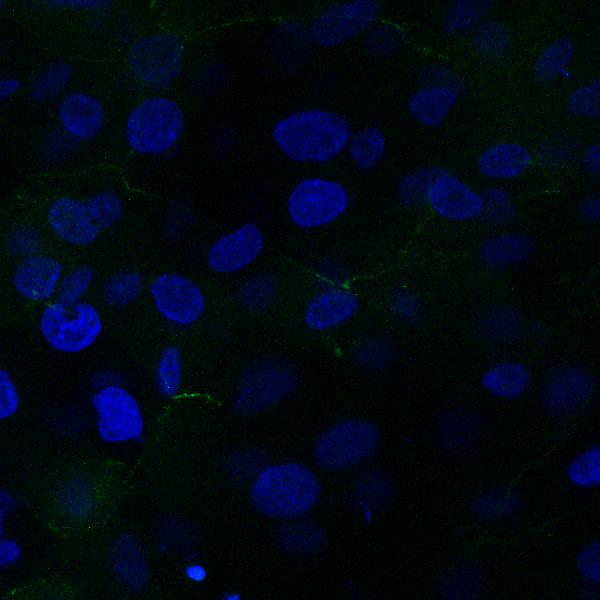

Supplement: Supplementary file 5 — Source data Fig. 2 [file 44321_2024_104_MOESM5_ESM.zip › Figure 2/2B/OCLN Veh/DDK Veh OCLN Merge.tif]

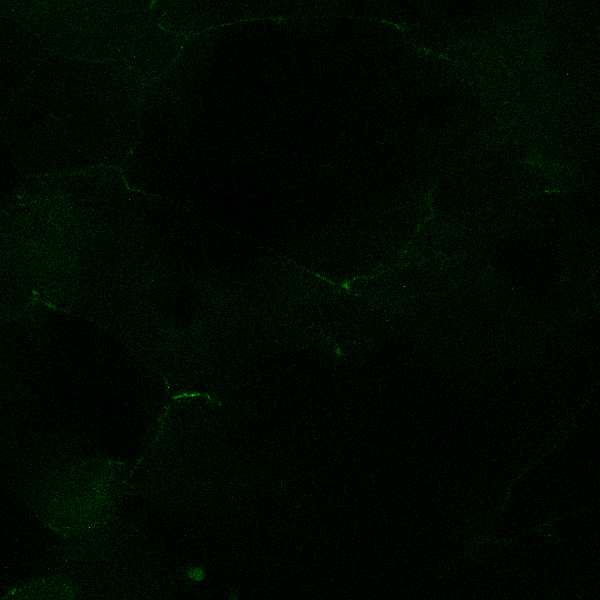

Supplement: Supplementary file 5 — Source data Fig. 2 [file 44321_2024_104_MOESM5_ESM.zip › Figure 2/2B/OCLN Veh/DDK Veh OCLN.tif]

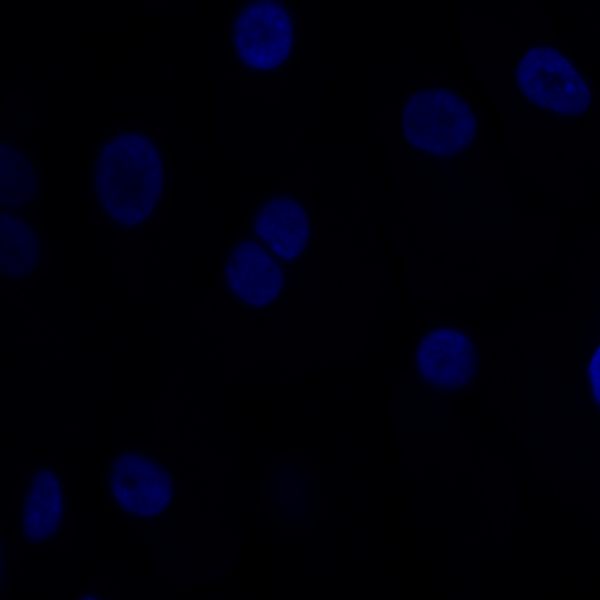

Supplement: Supplementary file 5 — Source data Fig. 2 [file 44321_2024_104_MOESM5_ESM.zip › Figure 2/2D/B-catenin Tg + Dl/Tg + Dl B-cat DAPI.tif]

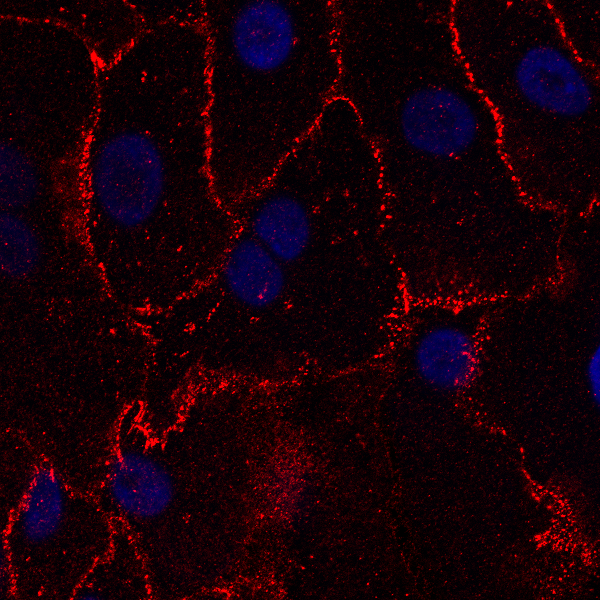

Supplement: Supplementary file 5 — Source data Fig. 2 [file 44321_2024_104_MOESM5_ESM.zip › Figure 2/2D/B-catenin Tg + Dl/Tg + Dl B-cat Merge.tif]

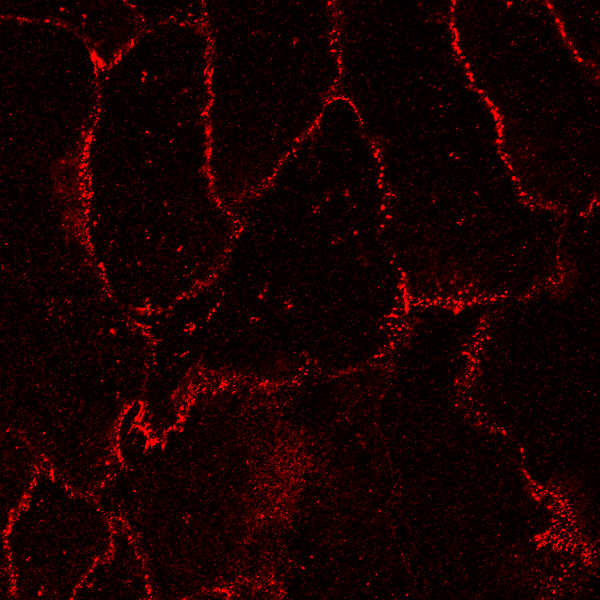

Supplement: Supplementary file 5 — Source data Fig. 2 [file 44321_2024_104_MOESM5_ESM.zip › Figure 2/2D/B-catenin Tg + Dl/Tg + Dl B-cat.tif]

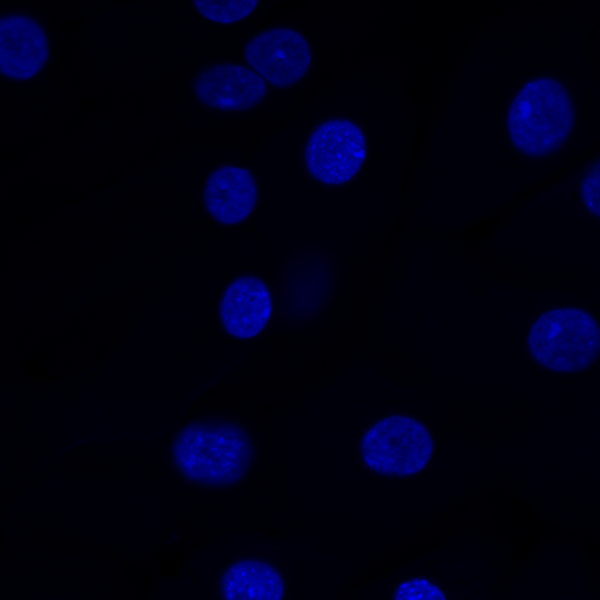

Supplement: Supplementary file 5 — Source data Fig. 2 [file 44321_2024_104_MOESM5_ESM.zip › Figure 2/2D/B-catenin Tg/Tg B-cat DAPI.tif]

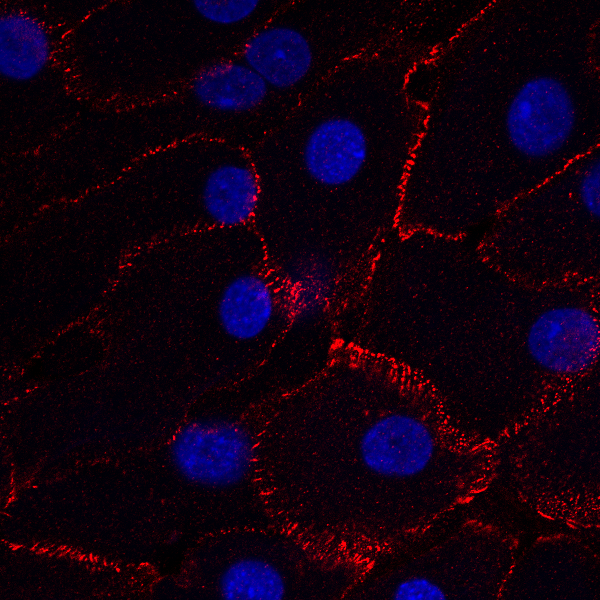

Supplement: Supplementary file 5 — Source data Fig. 2 [file 44321_2024_104_MOESM5_ESM.zip › Figure 2/2D/B-catenin Tg/Tg B-cat Merge.tif]

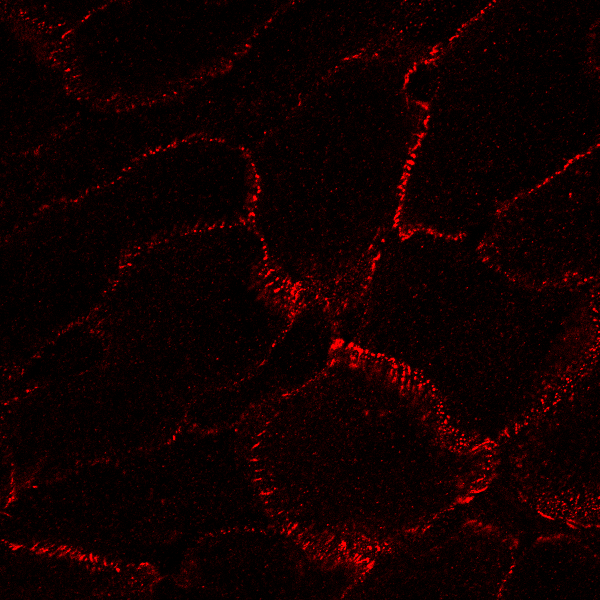

Supplement: Supplementary file 5 — Source data Fig. 2 [file 44321_2024_104_MOESM5_ESM.zip › Figure 2/2D/B-catenin Tg/Tg B-cat.tif]

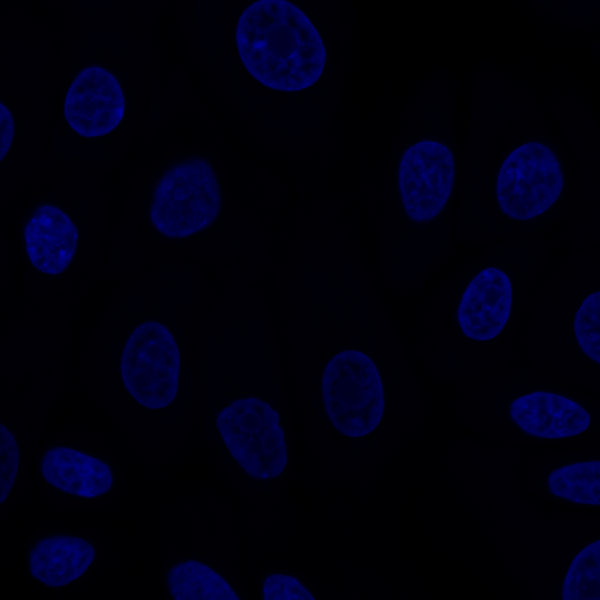

Supplement: Supplementary file 5 — Source data Fig. 2 [file 44321_2024_104_MOESM5_ESM.zip › Figure 2/2D/B-catenin Veh + Dl/Veh + Dl B-cat DAPI.tif]

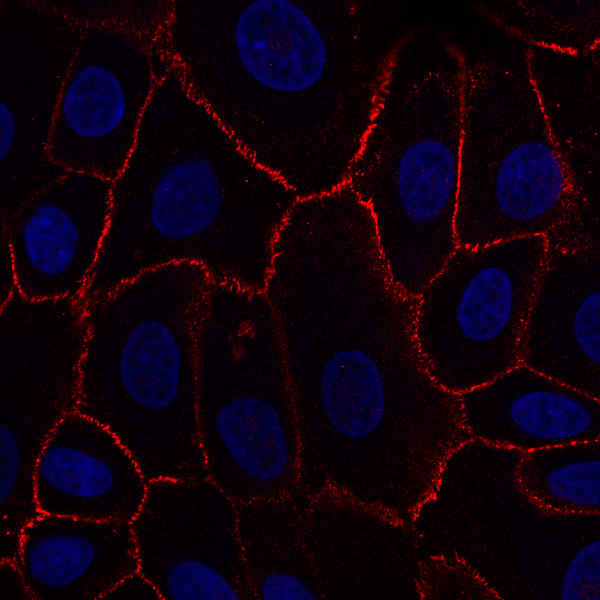

Supplement: Supplementary file 5 — Source data Fig. 2 [file 44321_2024_104_MOESM5_ESM.zip › Figure 2/2D/B-catenin Veh + Dl/Veh + Dl B-cat Merge.tif]

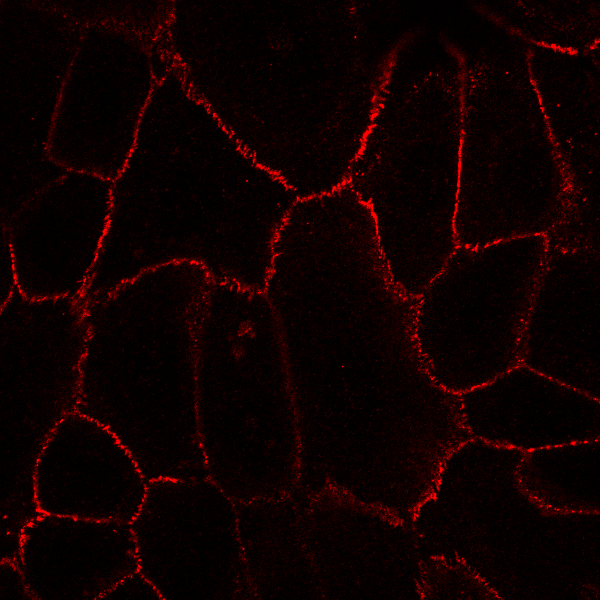

Supplement: Supplementary file 5 — Source data Fig. 2 [file 44321_2024_104_MOESM5_ESM.zip › Figure 2/2D/B-catenin Veh + Dl/Veh + Dl B-cat.tif]

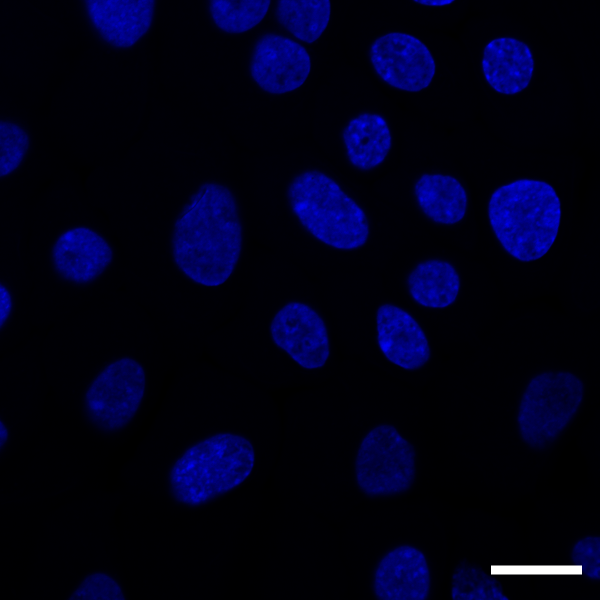

Supplement: Supplementary file 5 — Source data Fig. 2 [file 44321_2024_104_MOESM5_ESM.zip › Figure 2/2D/B-catenin Veh/Veh B-cat DAPI.tif]

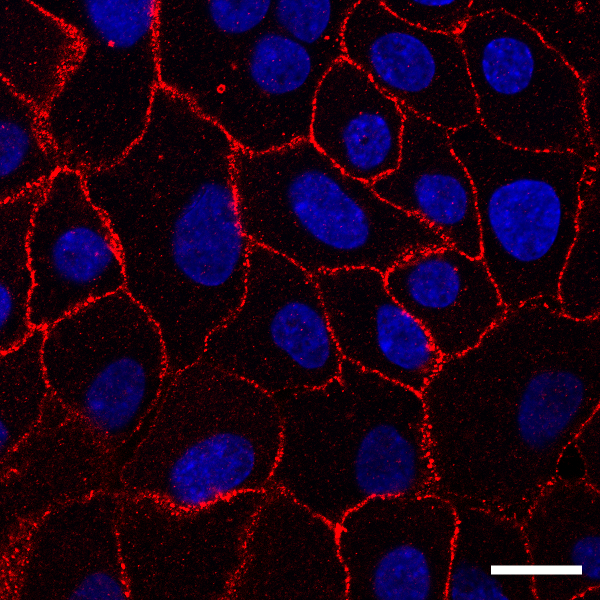

Supplement: Supplementary file 5 — Source data Fig. 2 [file 44321_2024_104_MOESM5_ESM.zip › Figure 2/2D/B-catenin Veh/Veh B-cat Merge.tif]

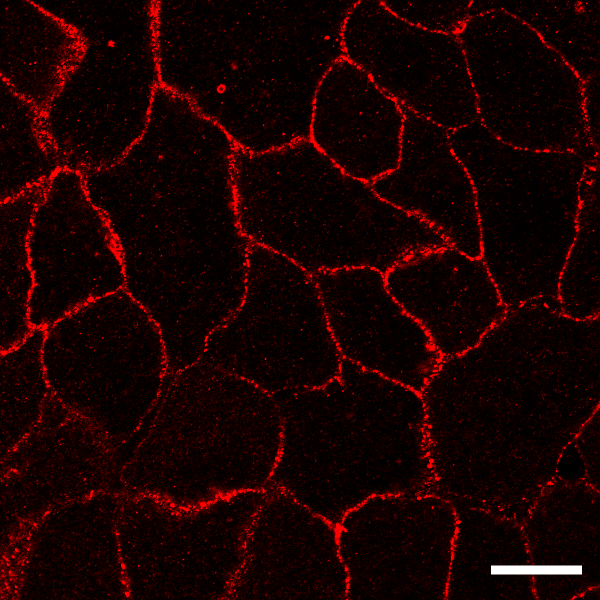

Supplement: Supplementary file 5 — Source data Fig. 2 [file 44321_2024_104_MOESM5_ESM.zip › Figure 2/2D/B-catenin Veh/Veh B-cat.tif]

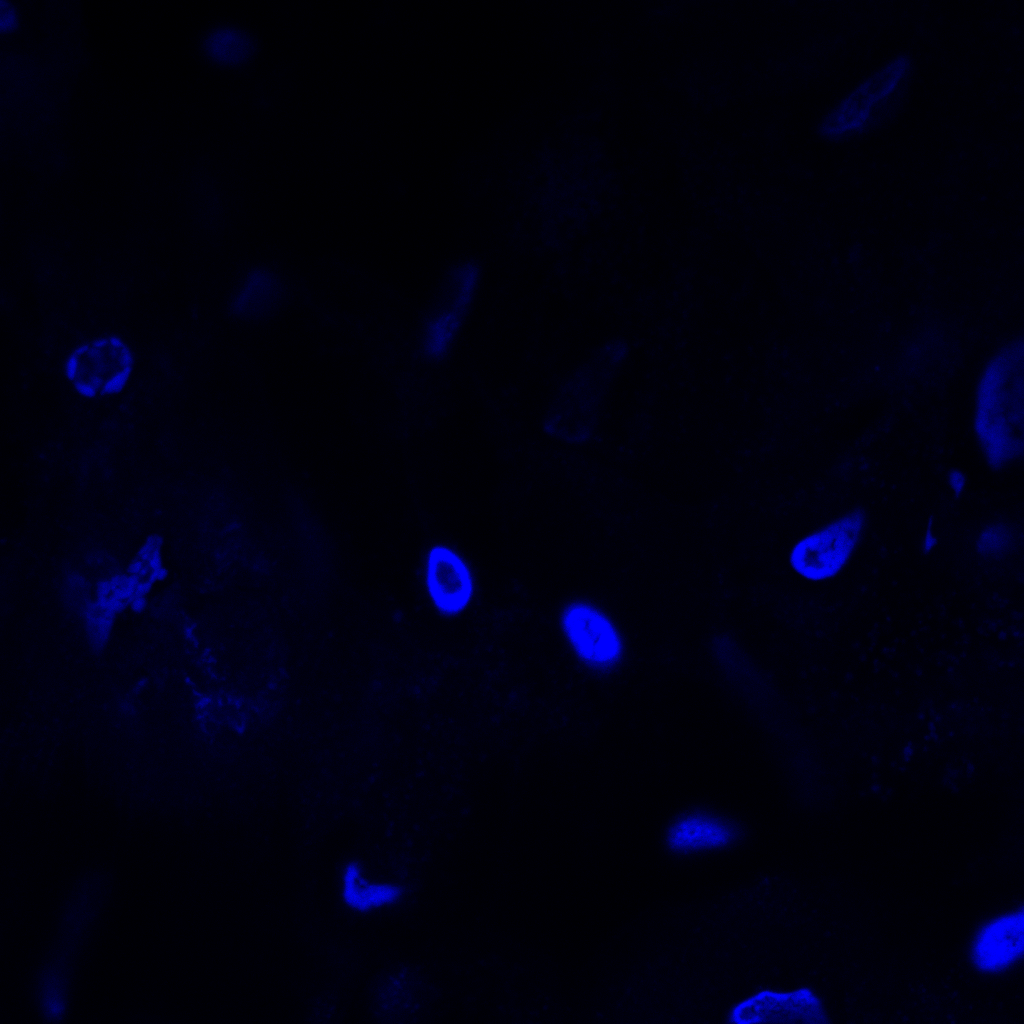

Supplement: Supplementary file 5 — Source data Fig. 2 [file 44321_2024_104_MOESM5_ESM.zip › Figure 2/2D/DSG1 Tg + Dl/Tg + Dl DSG1 DAPI.tif]

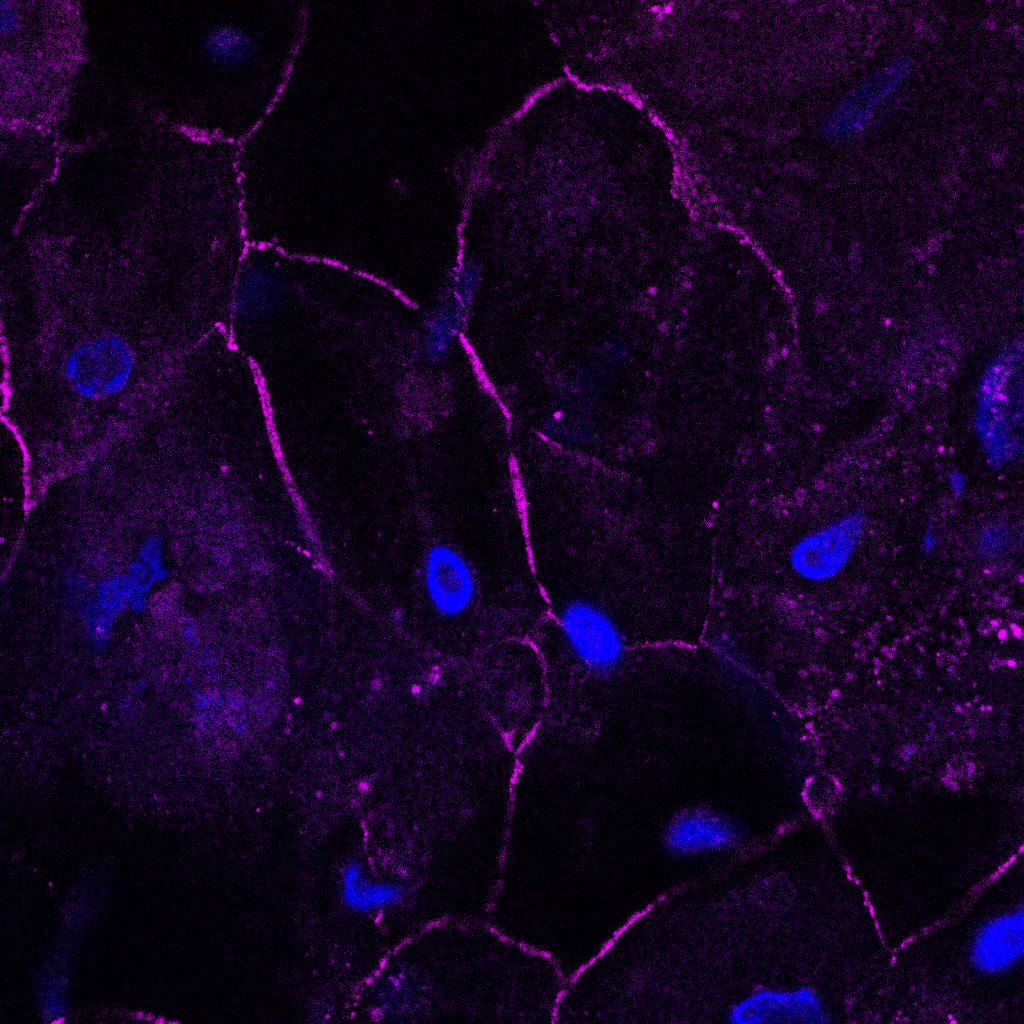

Supplement: Supplementary file 5 — Source data Fig. 2 [file 44321_2024_104_MOESM5_ESM.zip › Figure 2/2D/DSG1 Tg + Dl/Tg + Dl DSG1 Merge.tif]

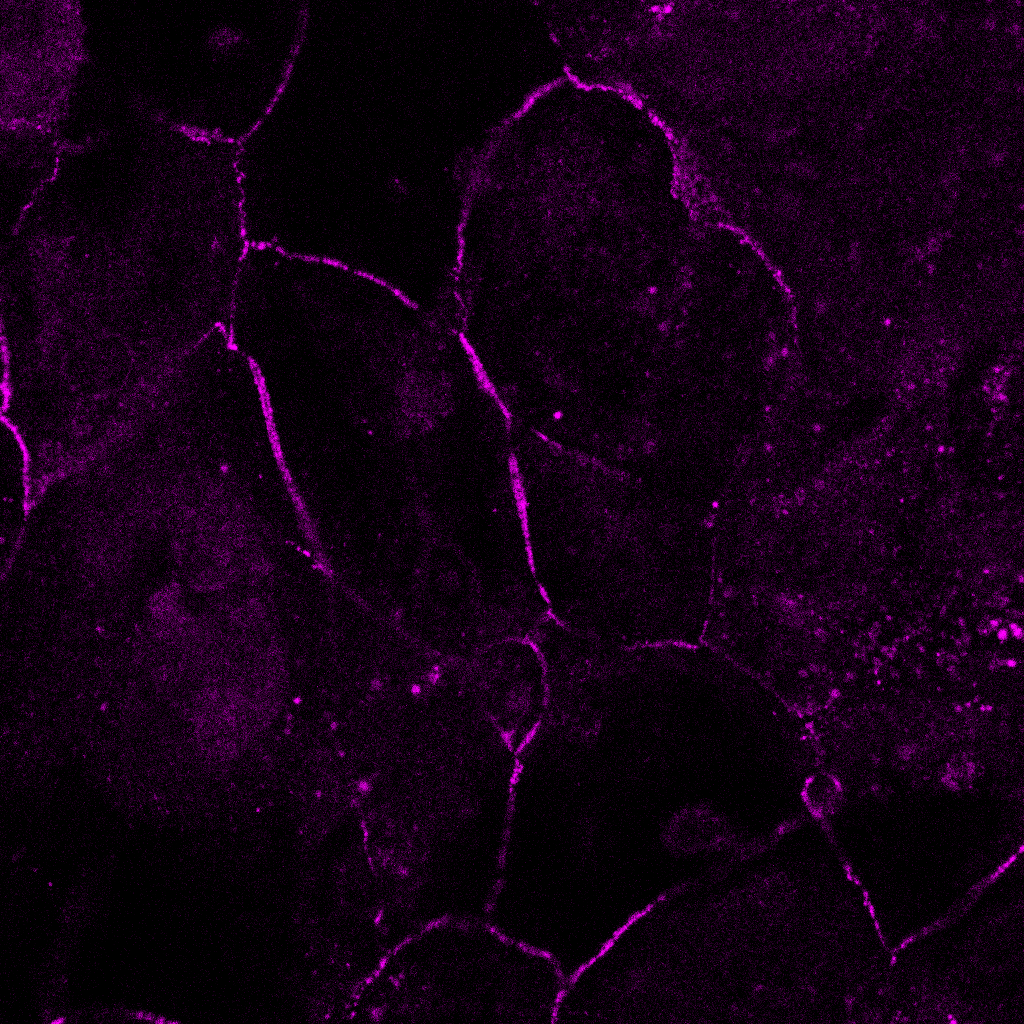

Supplement: Supplementary file 5 — Source data Fig. 2 [file 44321_2024_104_MOESM5_ESM.zip › Figure 2/2D/DSG1 Tg + Dl/Tg + Dl DSG1.tif]

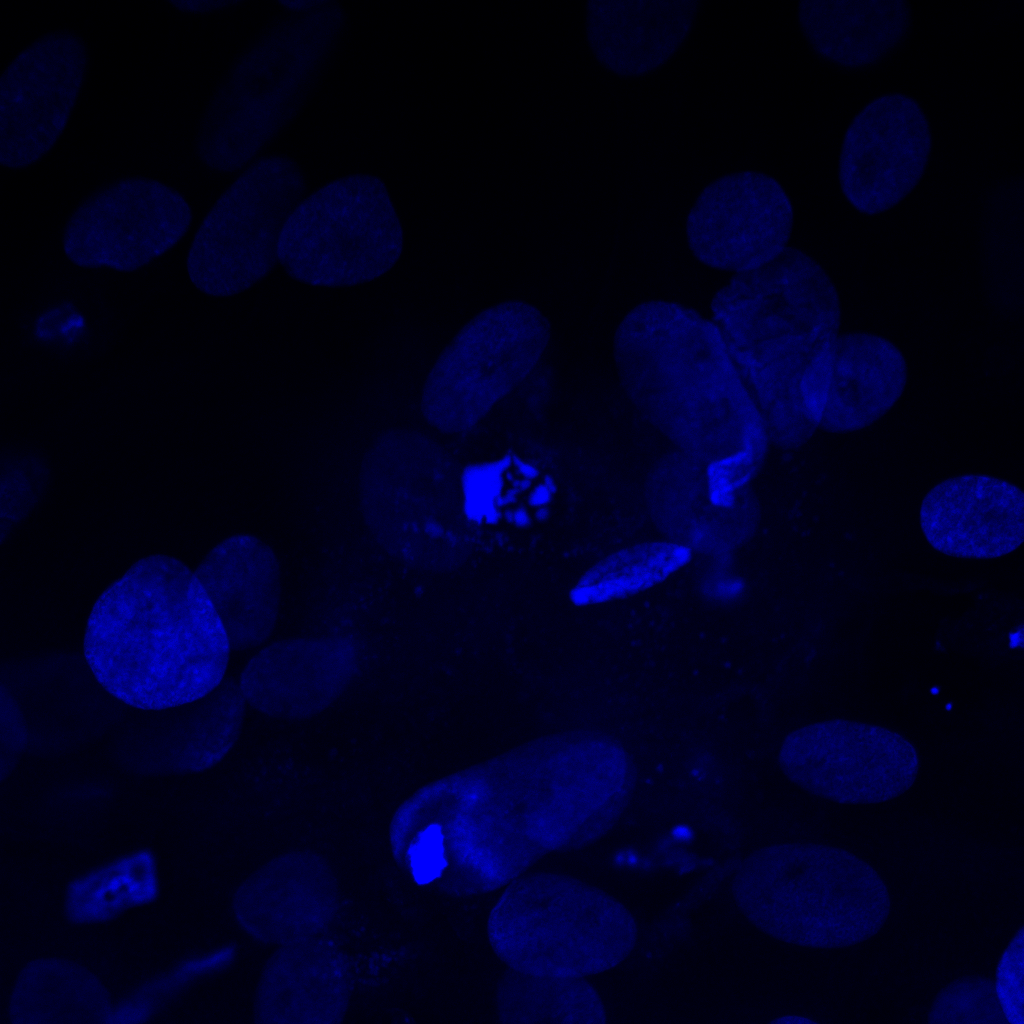

Supplement: Supplementary file 5 — Source data Fig. 2 [file 44321_2024_104_MOESM5_ESM.zip › Figure 2/2D/DSG1 Tg/Tg DSG1 DAPI.tif]

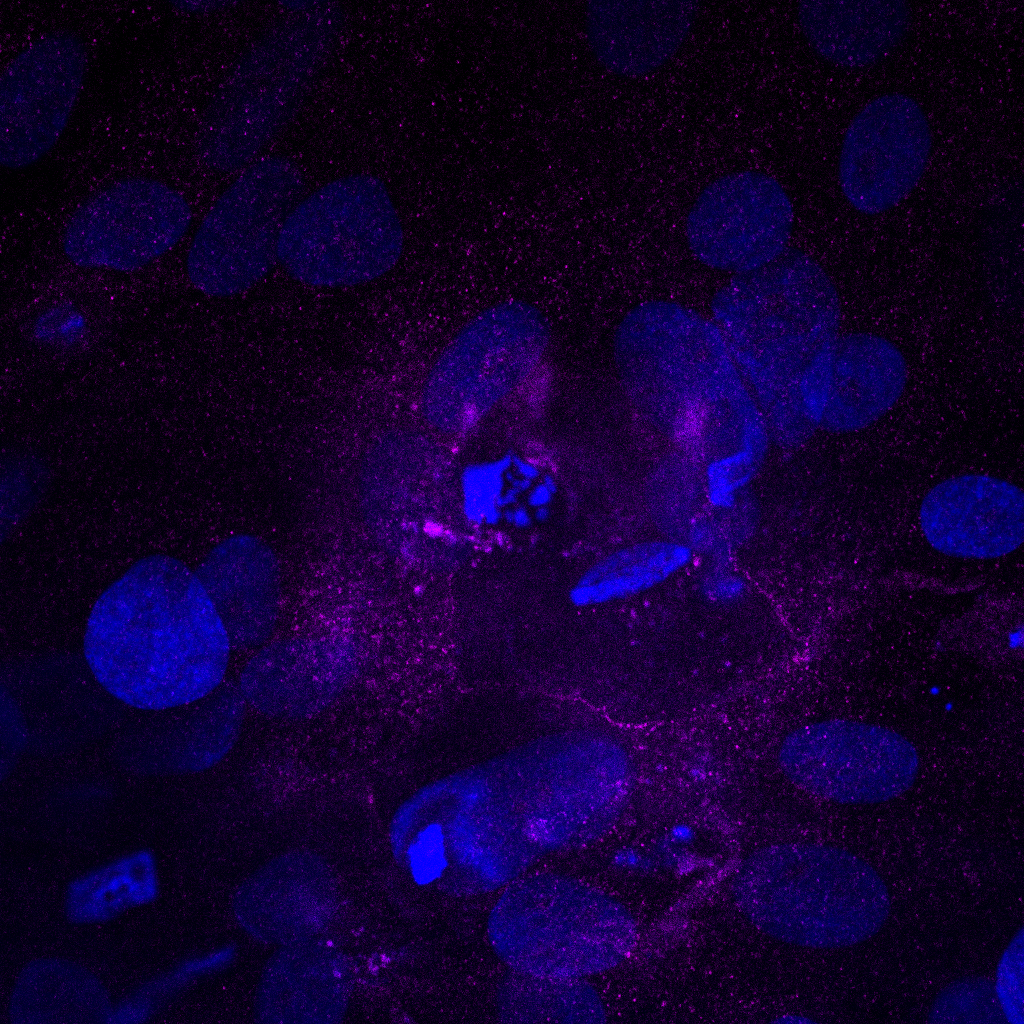

Supplement: Supplementary file 5 — Source data Fig. 2 [file 44321_2024_104_MOESM5_ESM.zip › Figure 2/2D/DSG1 Tg/Tg DSG1 Merge.tif]

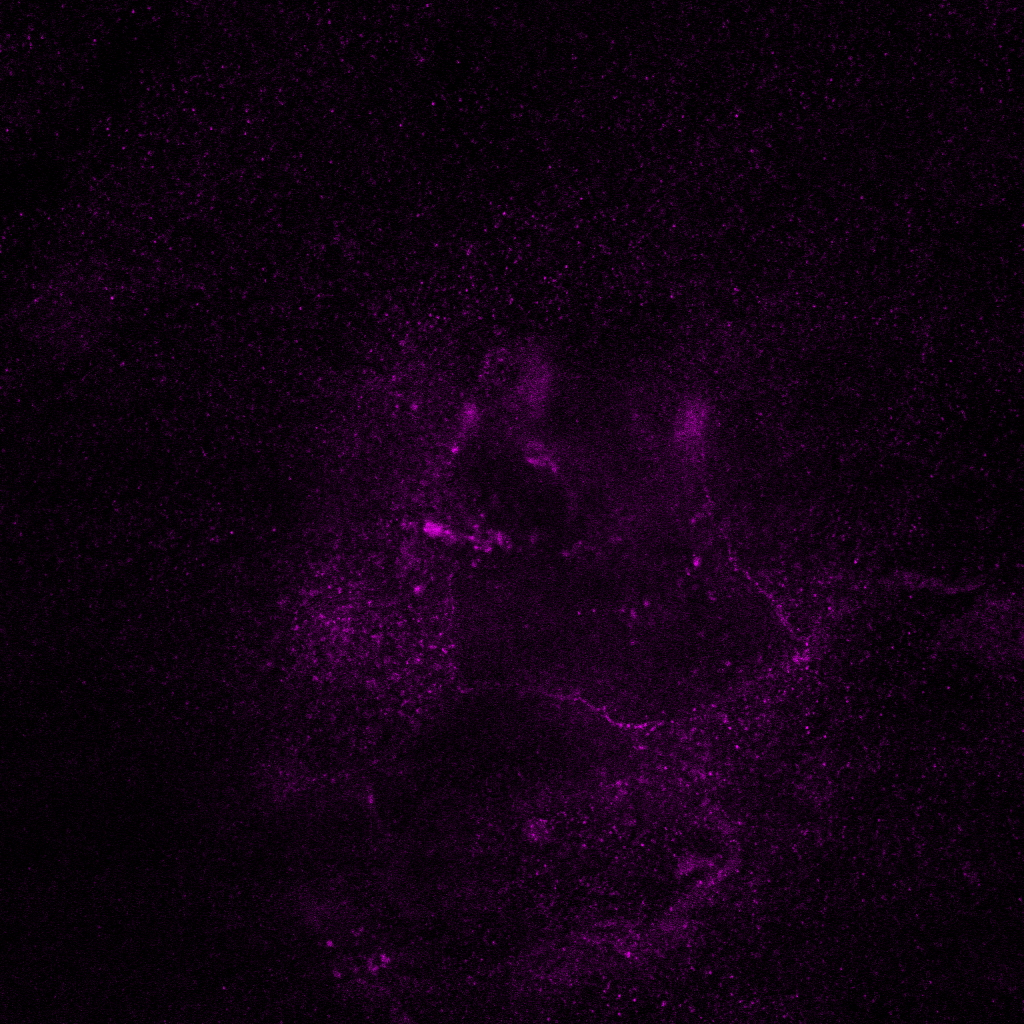

Supplement: Supplementary file 5 — Source data Fig. 2 [file 44321_2024_104_MOESM5_ESM.zip › Figure 2/2D/DSG1 Tg/Tg DSG1.tif]

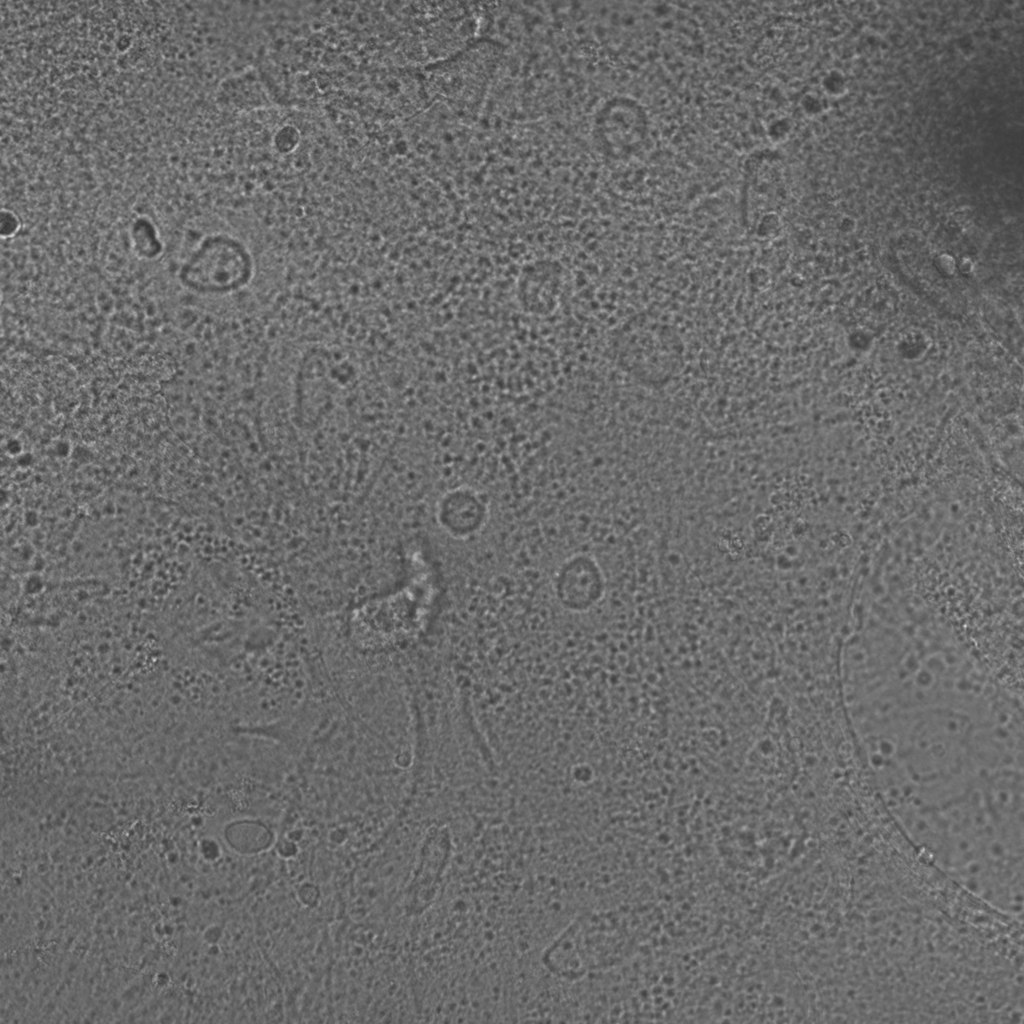

Supplement: Supplementary file 5 — Source data Fig. 2 [file 44321_2024_104_MOESM5_ESM.zip › Figure 2/2D/DSG1 Veh + Dl/Veh + Dl DSG1 brightfield.tif]

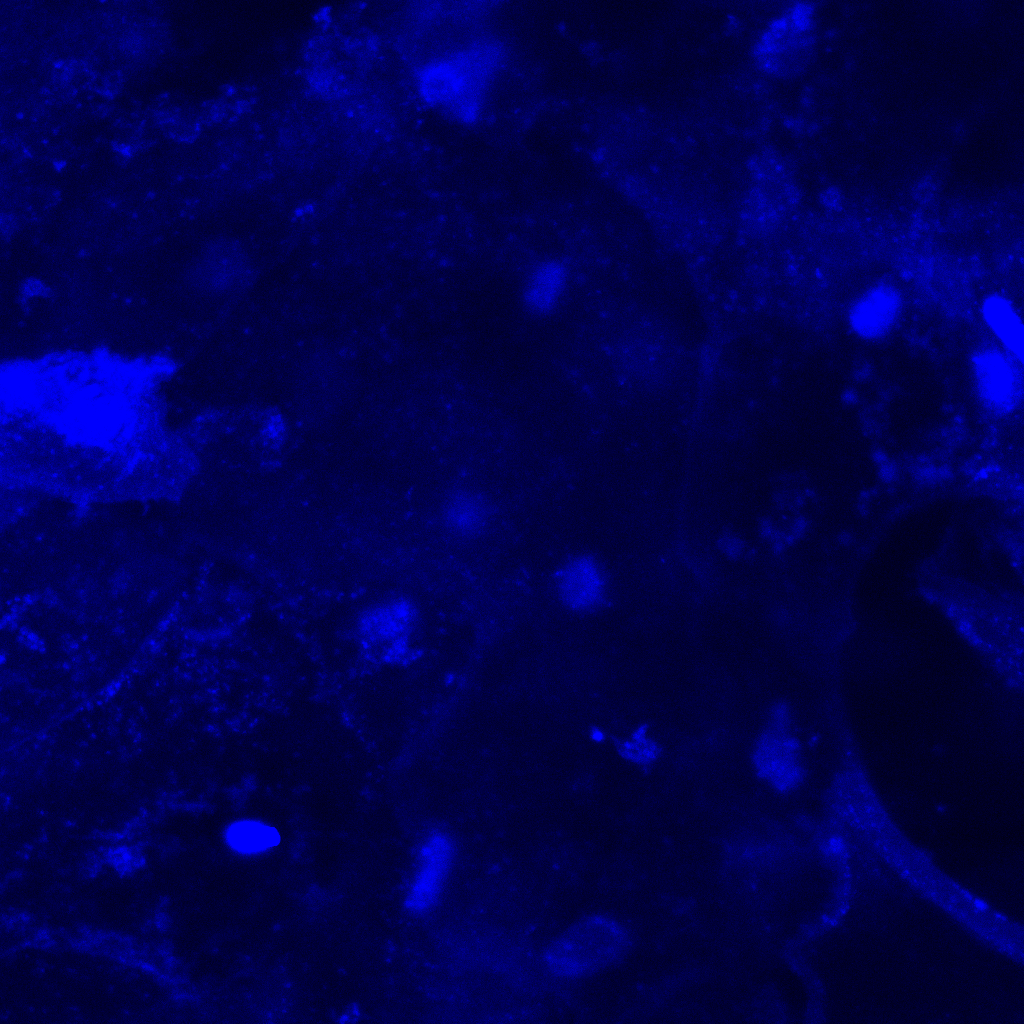

Supplement: Supplementary file 5 — Source data Fig. 2 [file 44321_2024_104_MOESM5_ESM.zip › Figure 2/2D/DSG1 Veh + Dl/Veh + Dl DSG1 DAPI.tif]

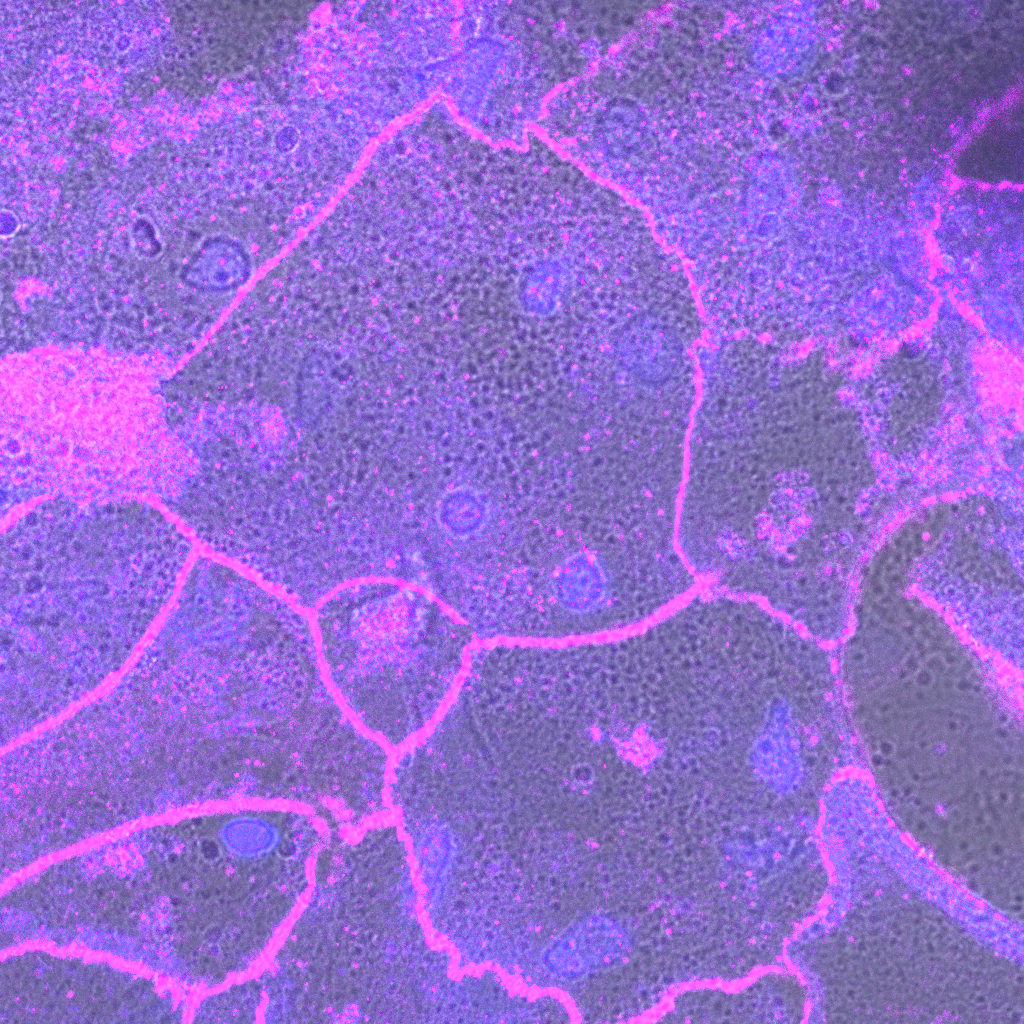

Supplement: Supplementary file 5 — Source data Fig. 2 [file 44321_2024_104_MOESM5_ESM.zip › Figure 2/2D/DSG1 Veh + Dl/Veh + Dl DSG1 Merge.tif]

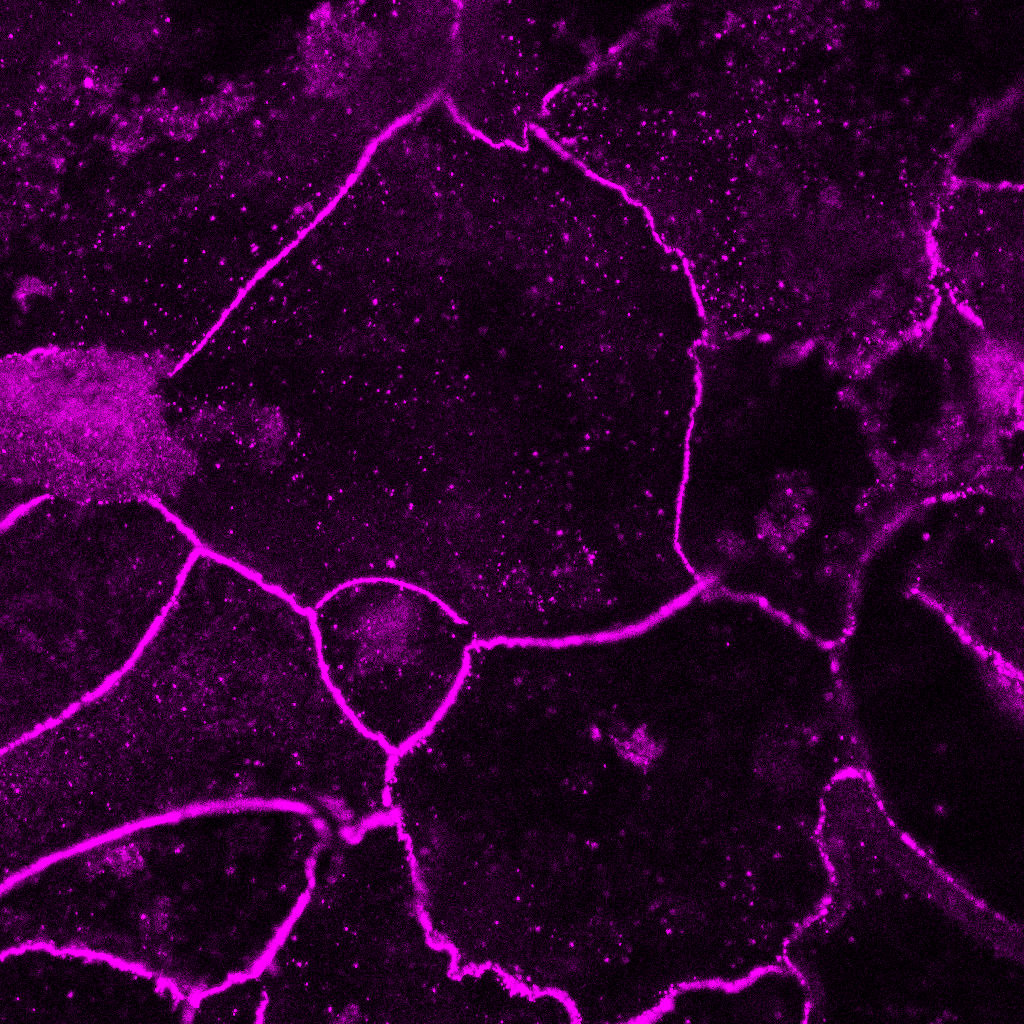

Supplement: Supplementary file 5 — Source data Fig. 2 [file 44321_2024_104_MOESM5_ESM.zip › Figure 2/2D/DSG1 Veh + Dl/Veh + Dl DSG1.tif]

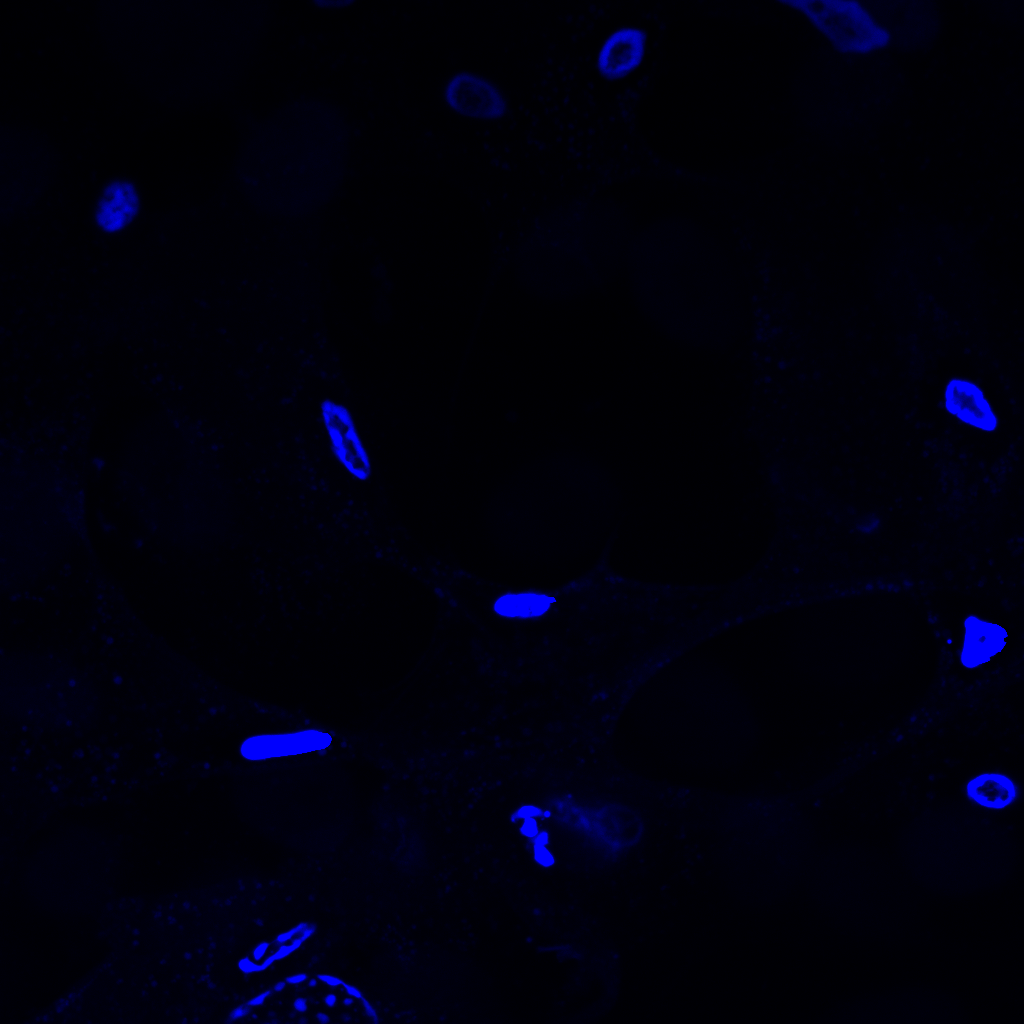

Supplement: Supplementary file 5 — Source data Fig. 2 [file 44321_2024_104_MOESM5_ESM.zip › Figure 2/2D/DSG1 Veh/Veh DSG1 DAPI.tif]

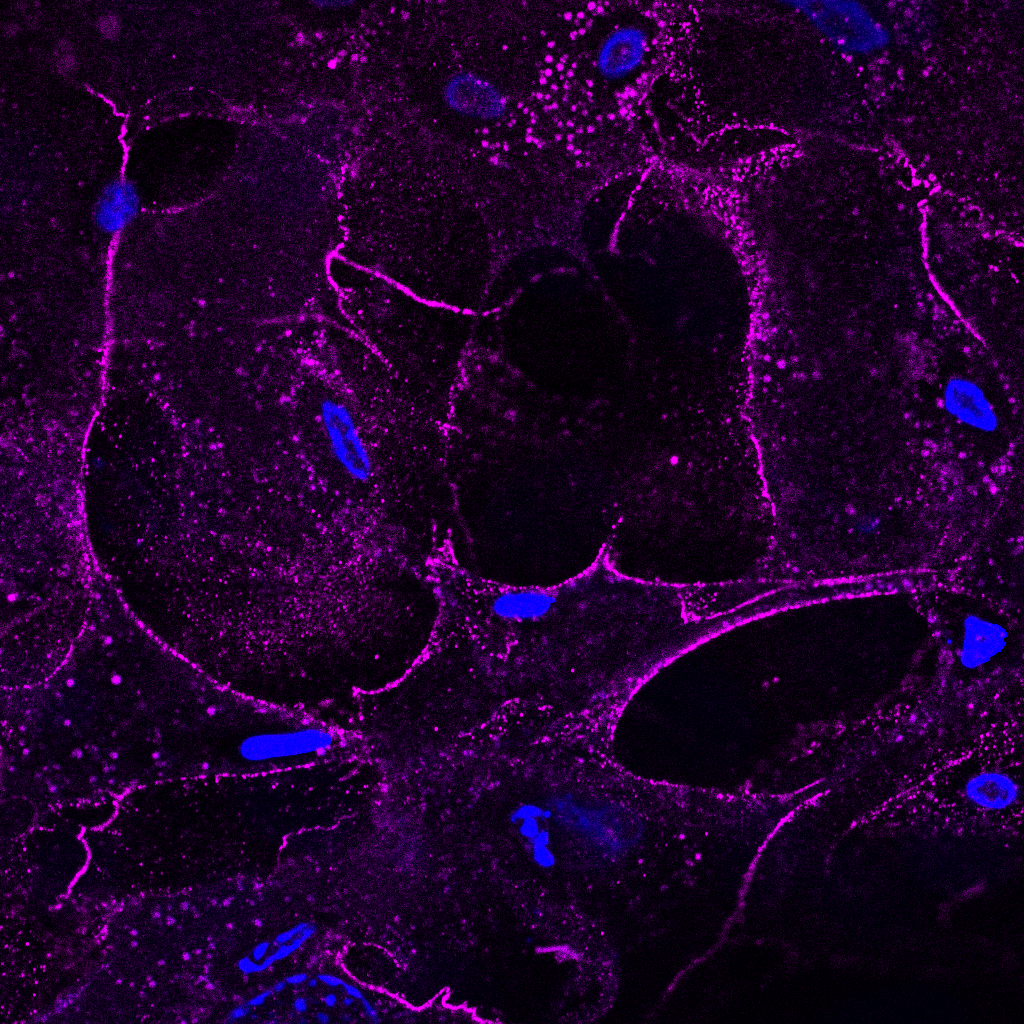

Supplement: Supplementary file 5 — Source data Fig. 2 [file 44321_2024_104_MOESM5_ESM.zip › Figure 2/2D/DSG1 Veh/Veh DSG1 Merge.tif]

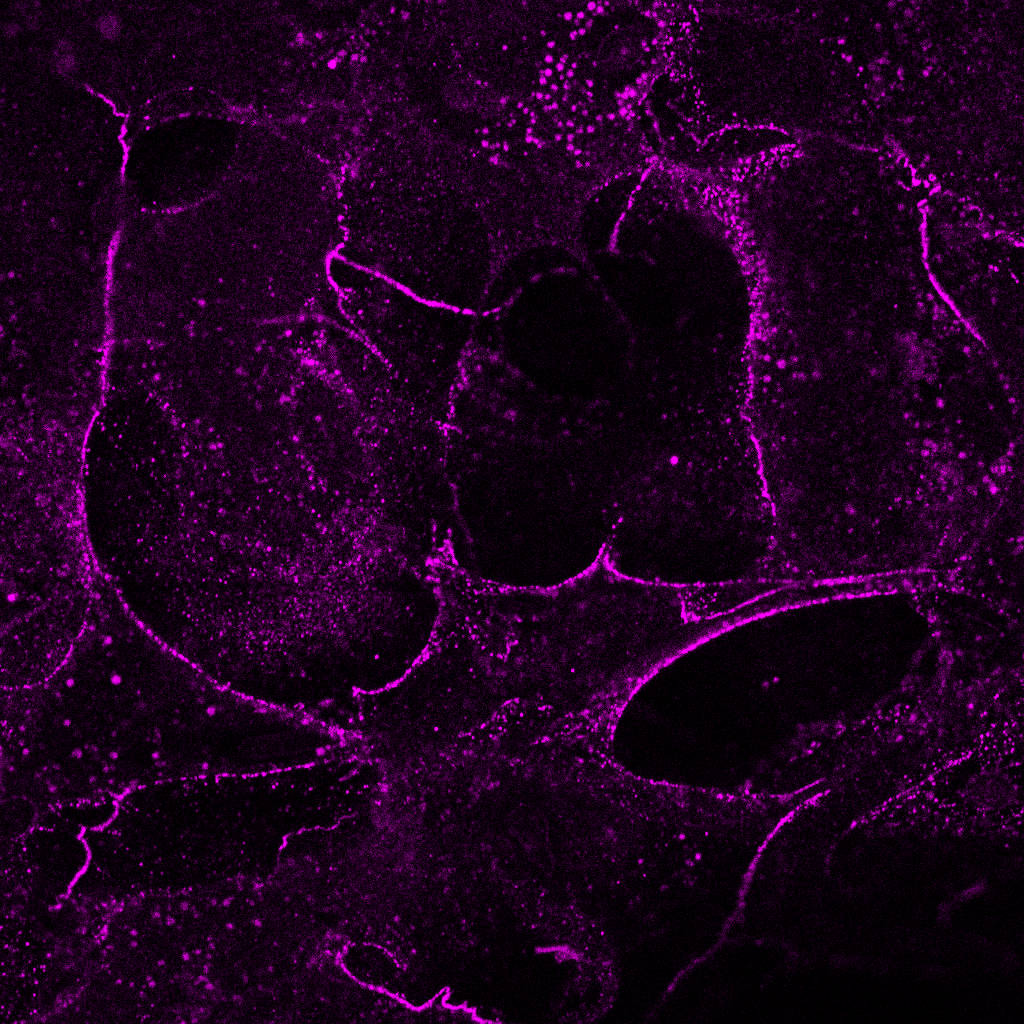

Supplement: Supplementary file 5 — Source data Fig. 2 [file 44321_2024_104_MOESM5_ESM.zip › Figure 2/2D/DSG1 Veh/Veh DSG1.tif]

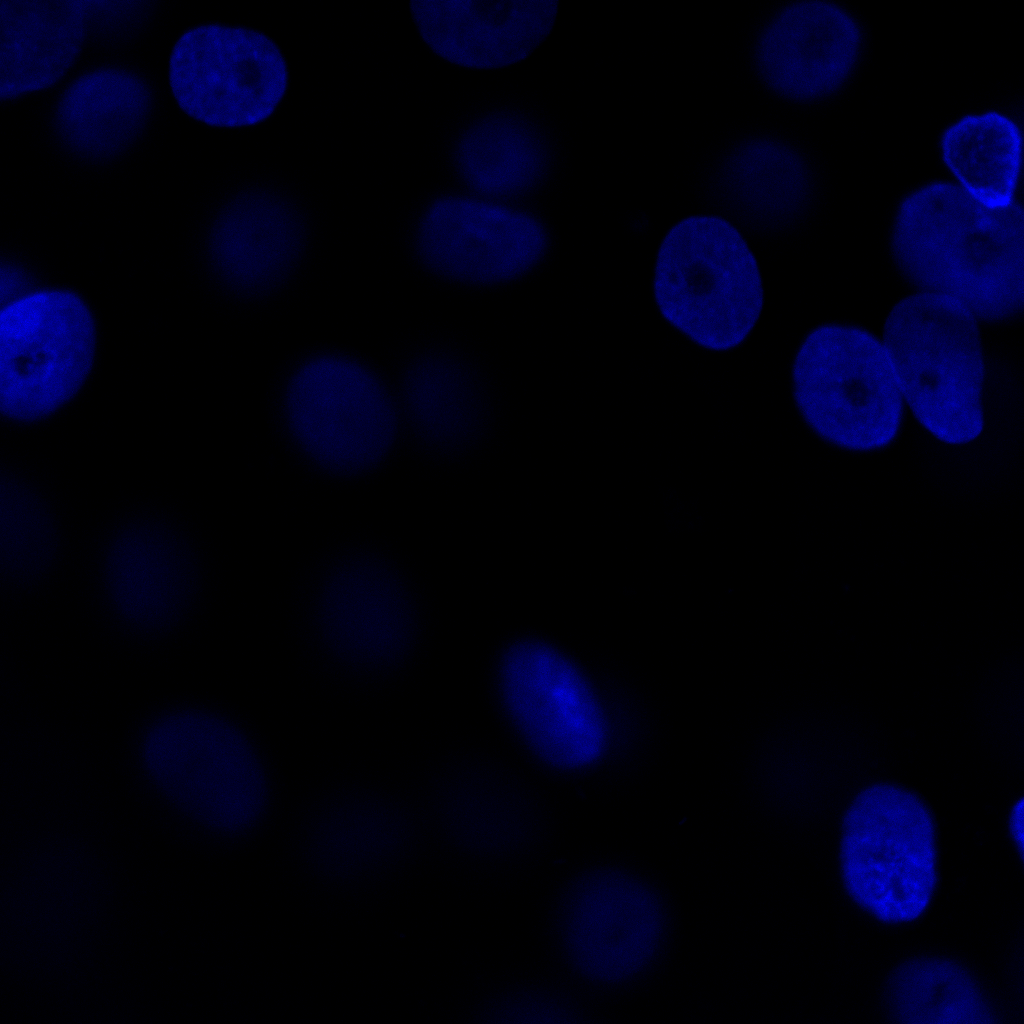

Supplement: Supplementary file 5 — Source data Fig. 2 [file 44321_2024_104_MOESM5_ESM.zip › Figure 2/2D/OCLN Tg + Dl/Tg + Dl OCLN DAPI.tif]

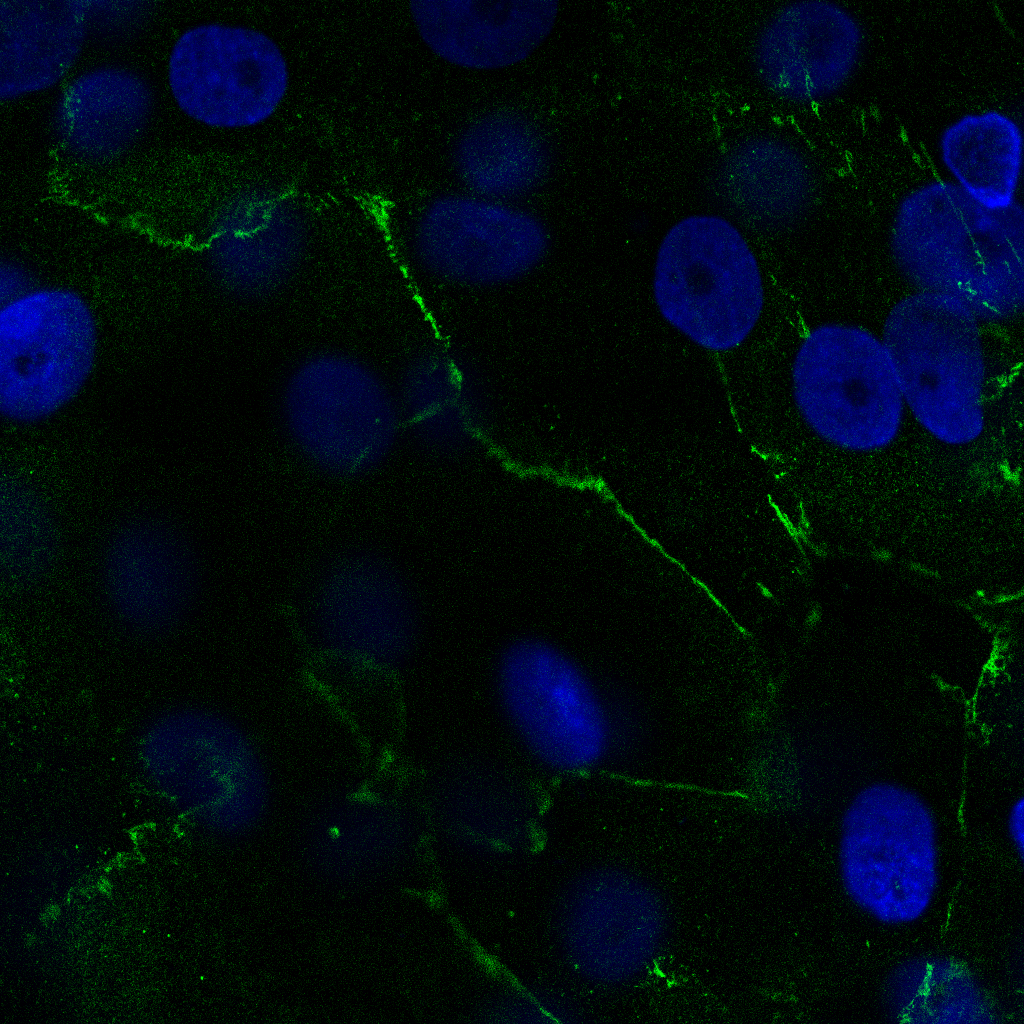

Supplement: Supplementary file 5 — Source data Fig. 2 [file 44321_2024_104_MOESM5_ESM.zip › Figure 2/2D/OCLN Tg + Dl/Tg + Dl OCLN Merge.tif]

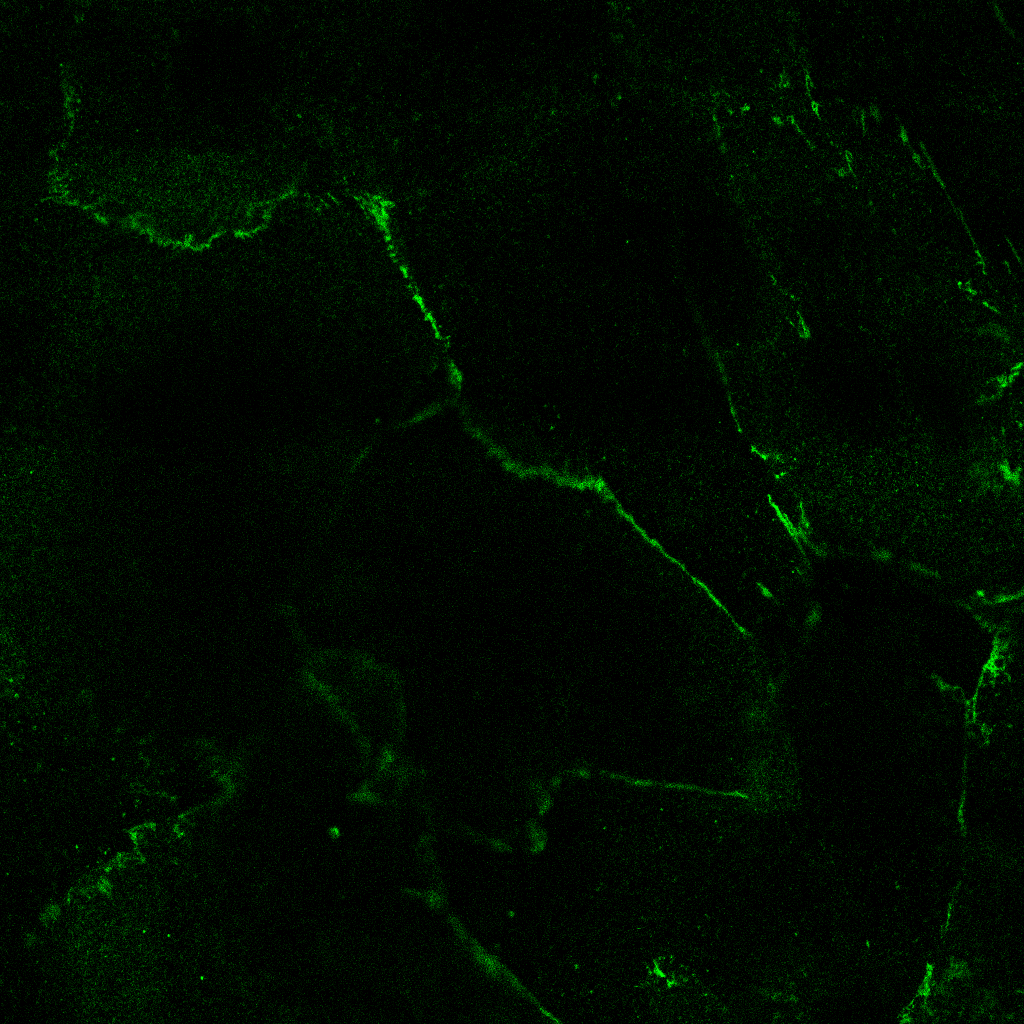

Supplement: Supplementary file 5 — Source data Fig. 2 [file 44321_2024_104_MOESM5_ESM.zip › Figure 2/2D/OCLN Tg + Dl/Tg + Dl OCLN.tif]

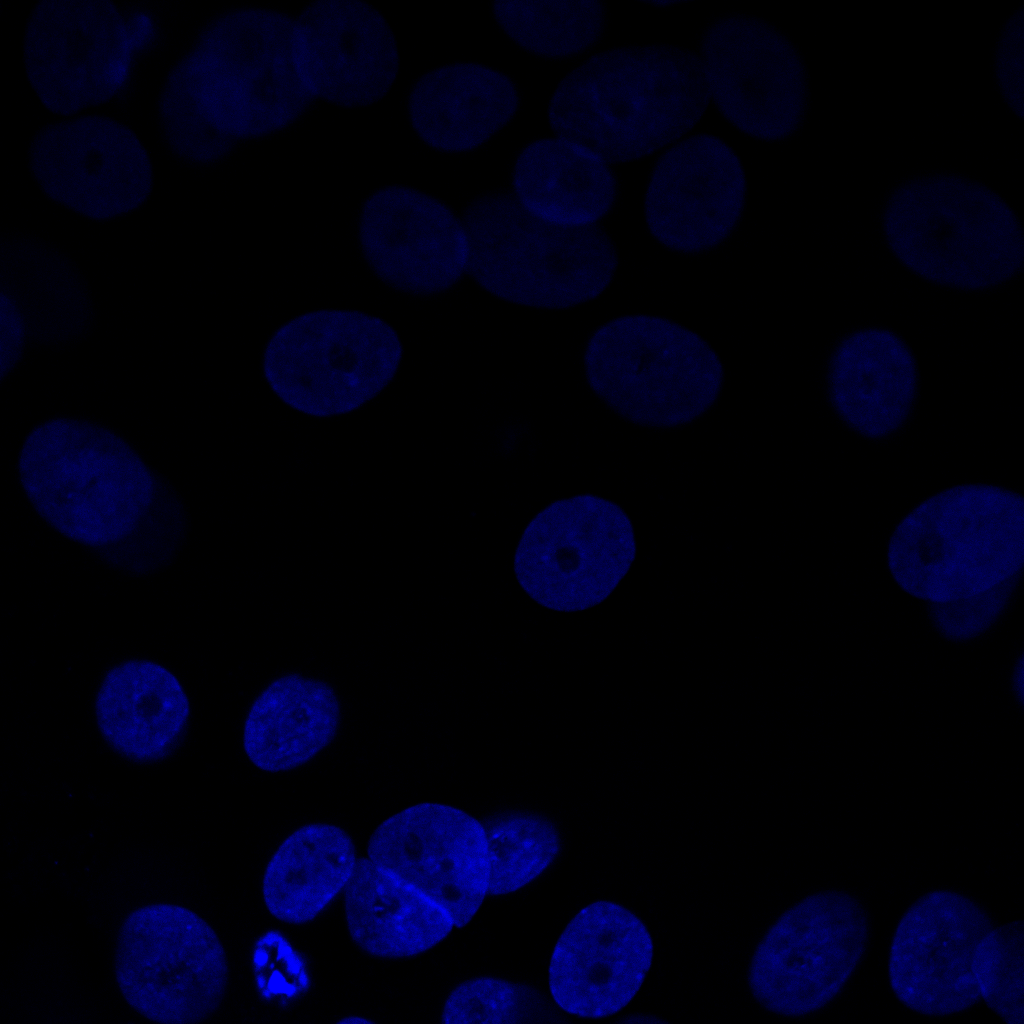

Supplement: Supplementary file 5 — Source data Fig. 2 [file 44321_2024_104_MOESM5_ESM.zip › Figure 2/2D/OCLN Tg/Tg OCLN DAPI.tif]

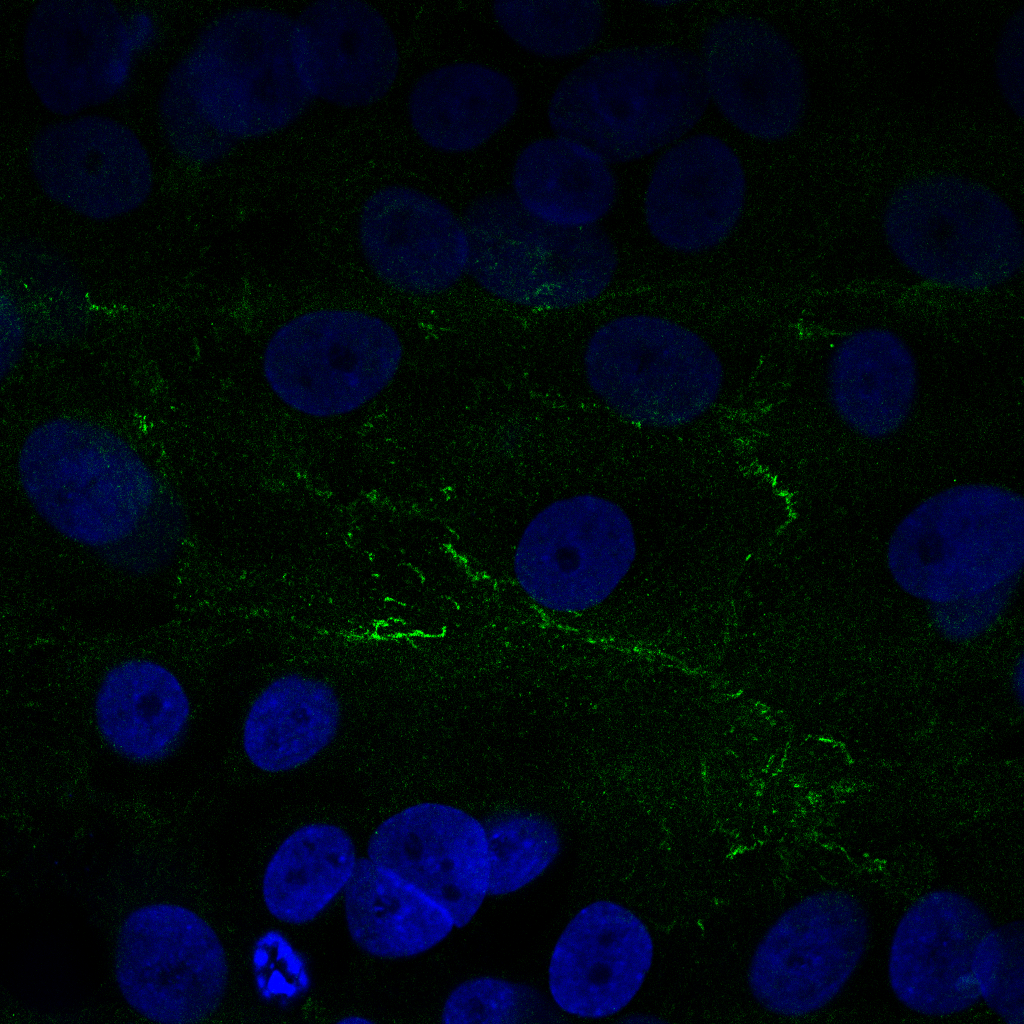

Supplement: Supplementary file 5 — Source data Fig. 2 [file 44321_2024_104_MOESM5_ESM.zip › Figure 2/2D/OCLN Tg/Tg OCLN Merge.tif]

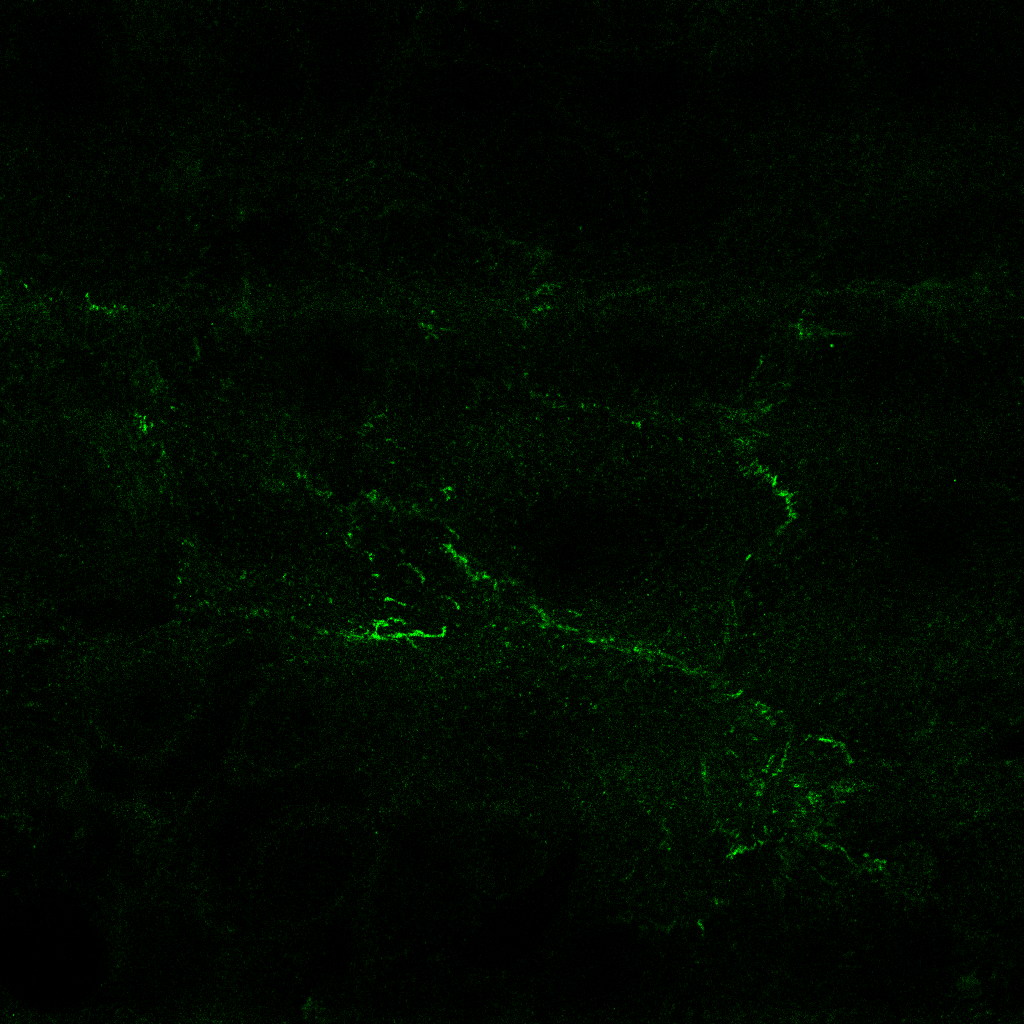

Supplement: Supplementary file 5 — Source data Fig. 2 [file 44321_2024_104_MOESM5_ESM.zip › Figure 2/2D/OCLN Tg/Tg OCLN.tif]

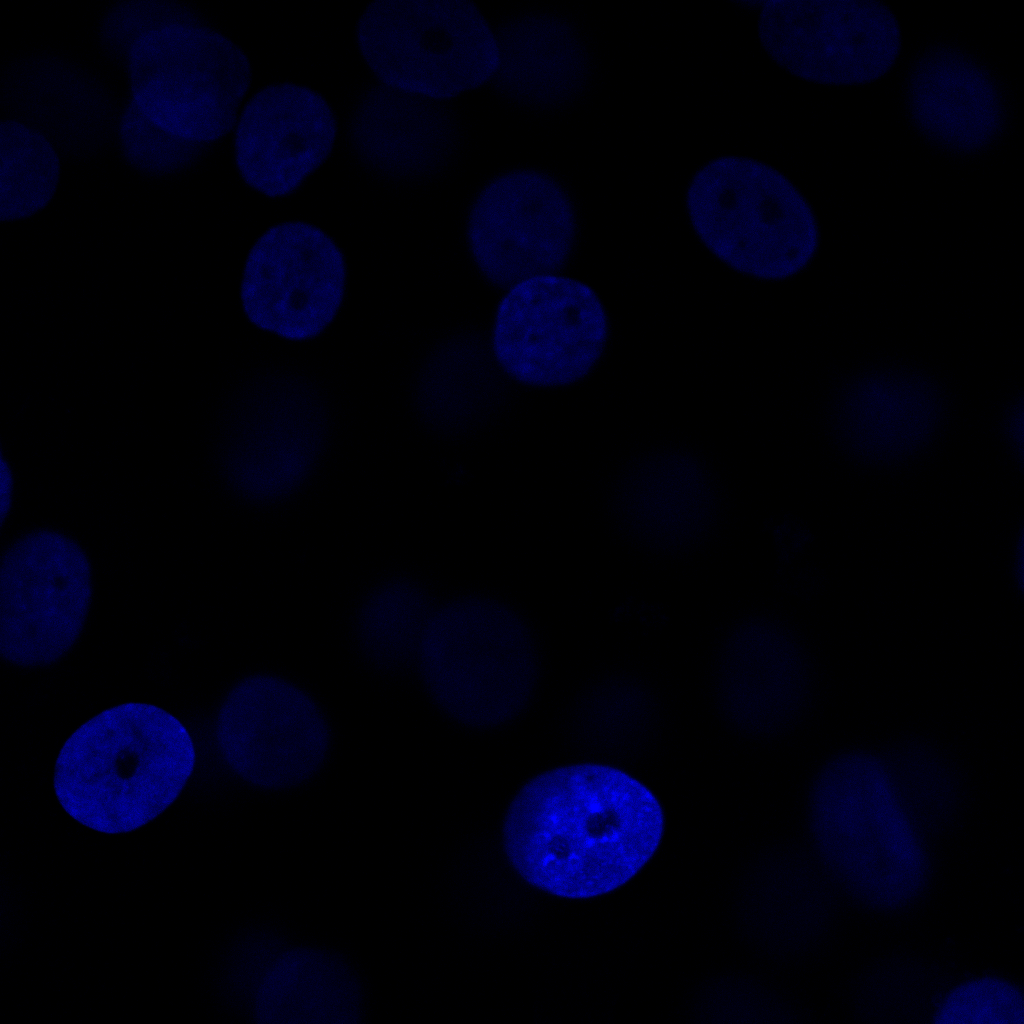

Supplement: Supplementary file 5 — Source data Fig. 2 [file 44321_2024_104_MOESM5_ESM.zip › Figure 2/2D/OCLN Veh + Dl/Veh + Dl OCLN DAPI.tif]

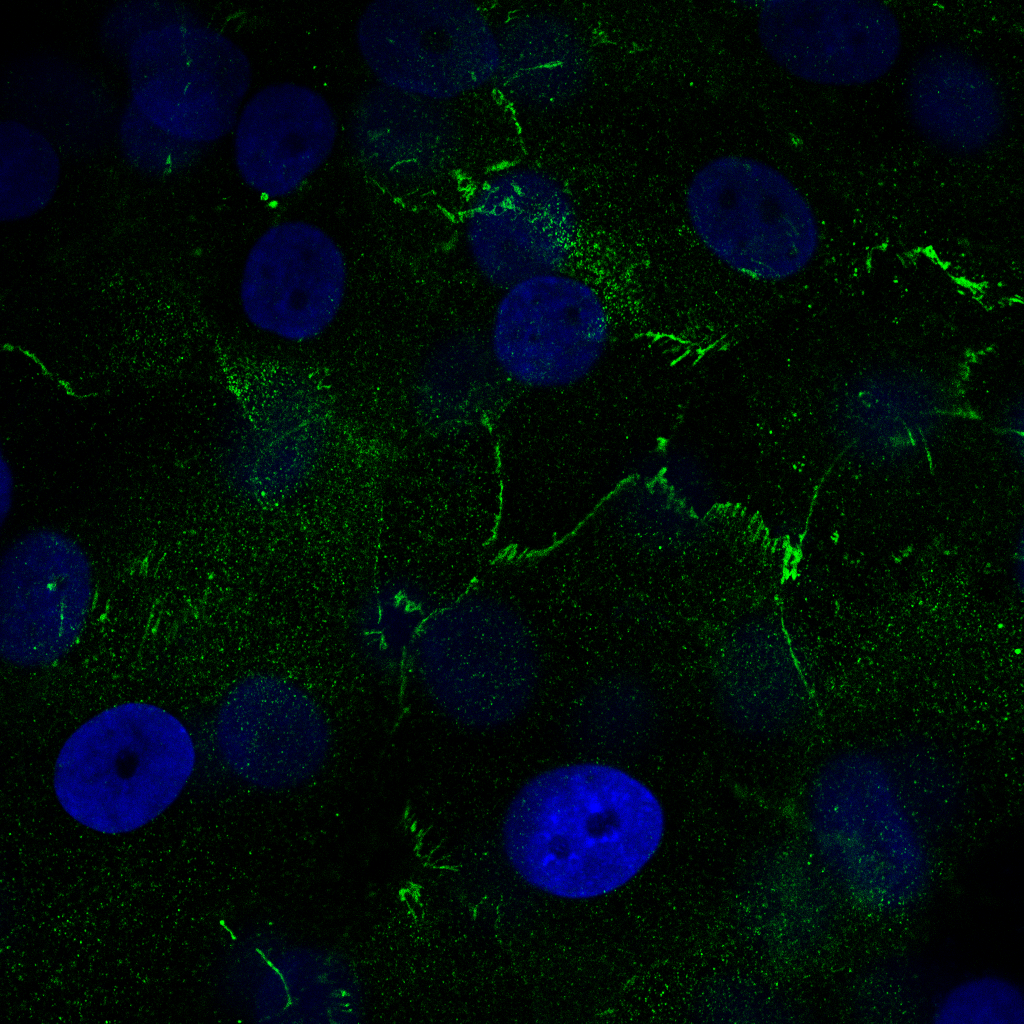

Supplement: Supplementary file 5 — Source data Fig. 2 [file 44321_2024_104_MOESM5_ESM.zip › Figure 2/2D/OCLN Veh + Dl/Veh + Dl OCLN Merge.tif]

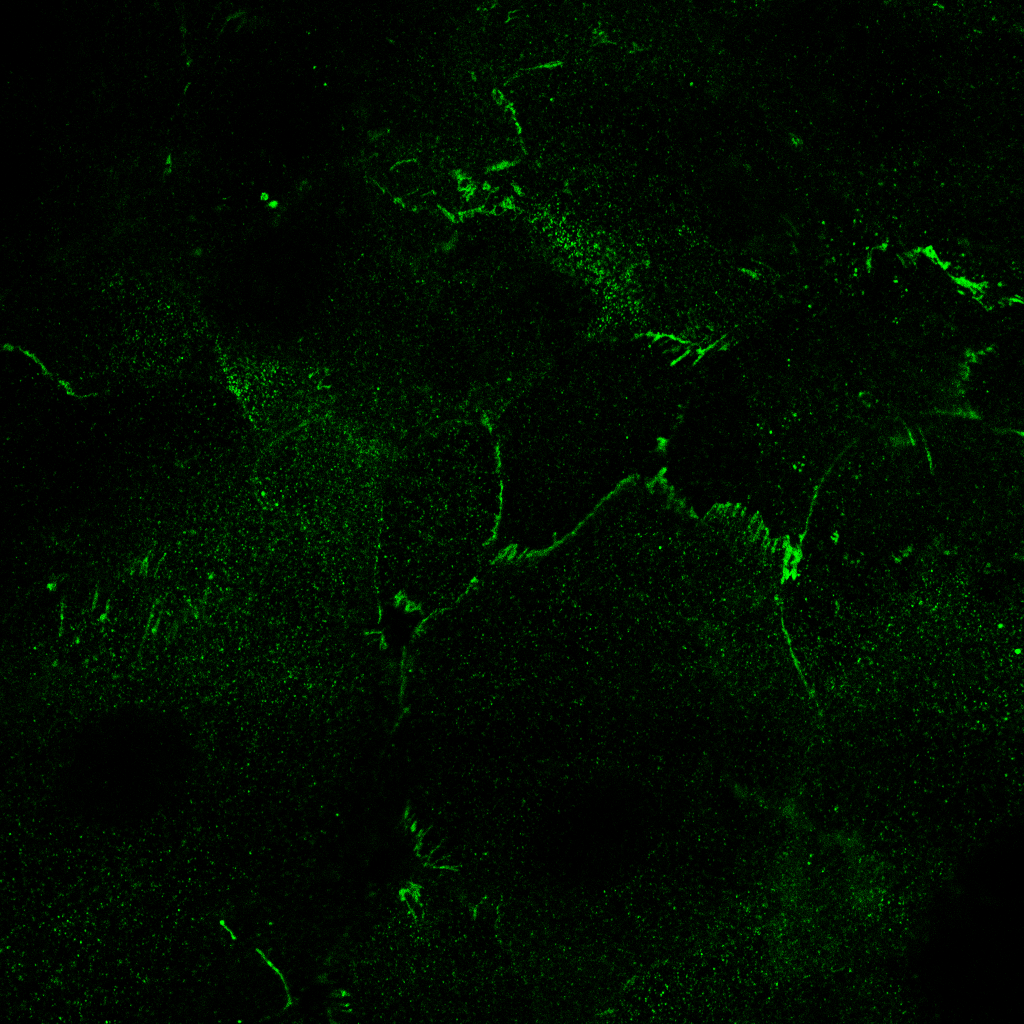

Supplement: Supplementary file 5 — Source data Fig. 2 [file 44321_2024_104_MOESM5_ESM.zip › Figure 2/2D/OCLN Veh + Dl/Veh + Dl OCLN.tif]

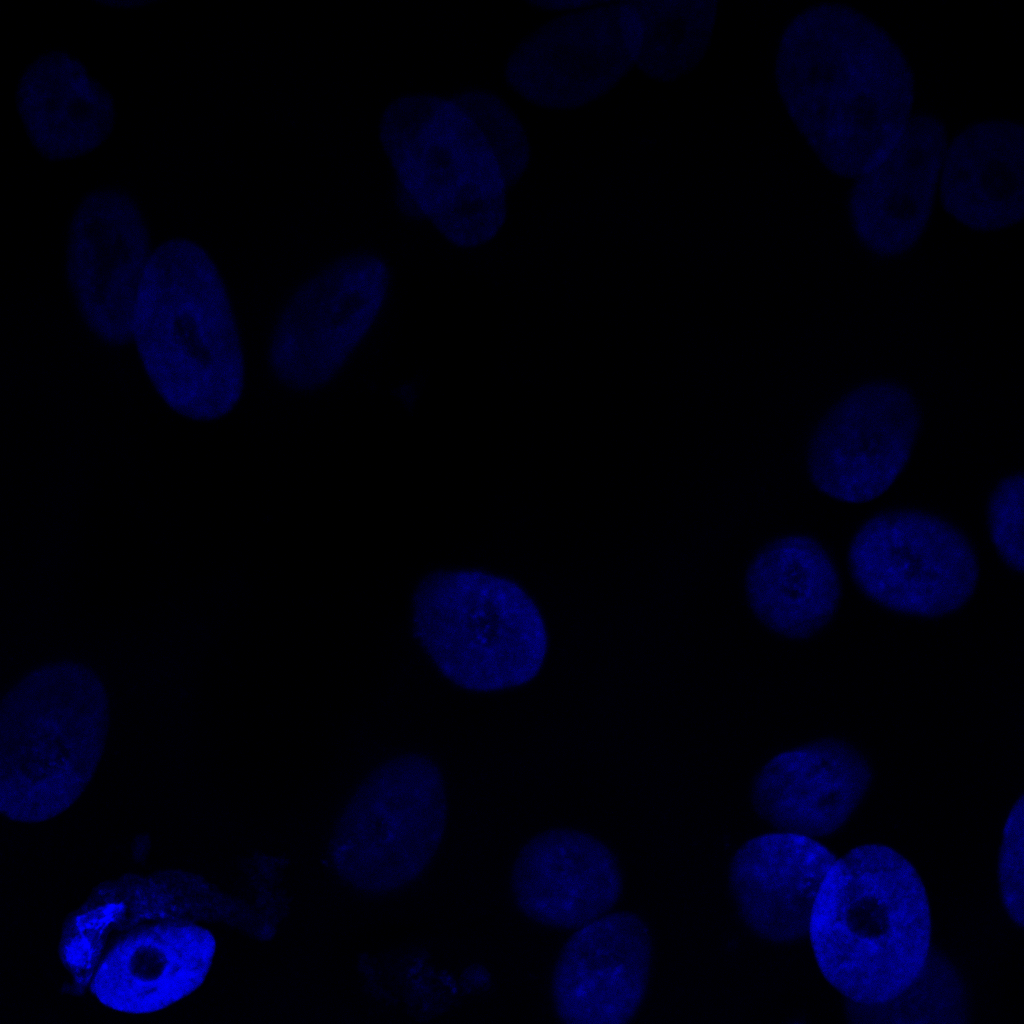

Supplement: Supplementary file 5 — Source data Fig. 2 [file 44321_2024_104_MOESM5_ESM.zip › Figure 2/2D/OCLN Veh/Veh OCLN DAPI.tif]

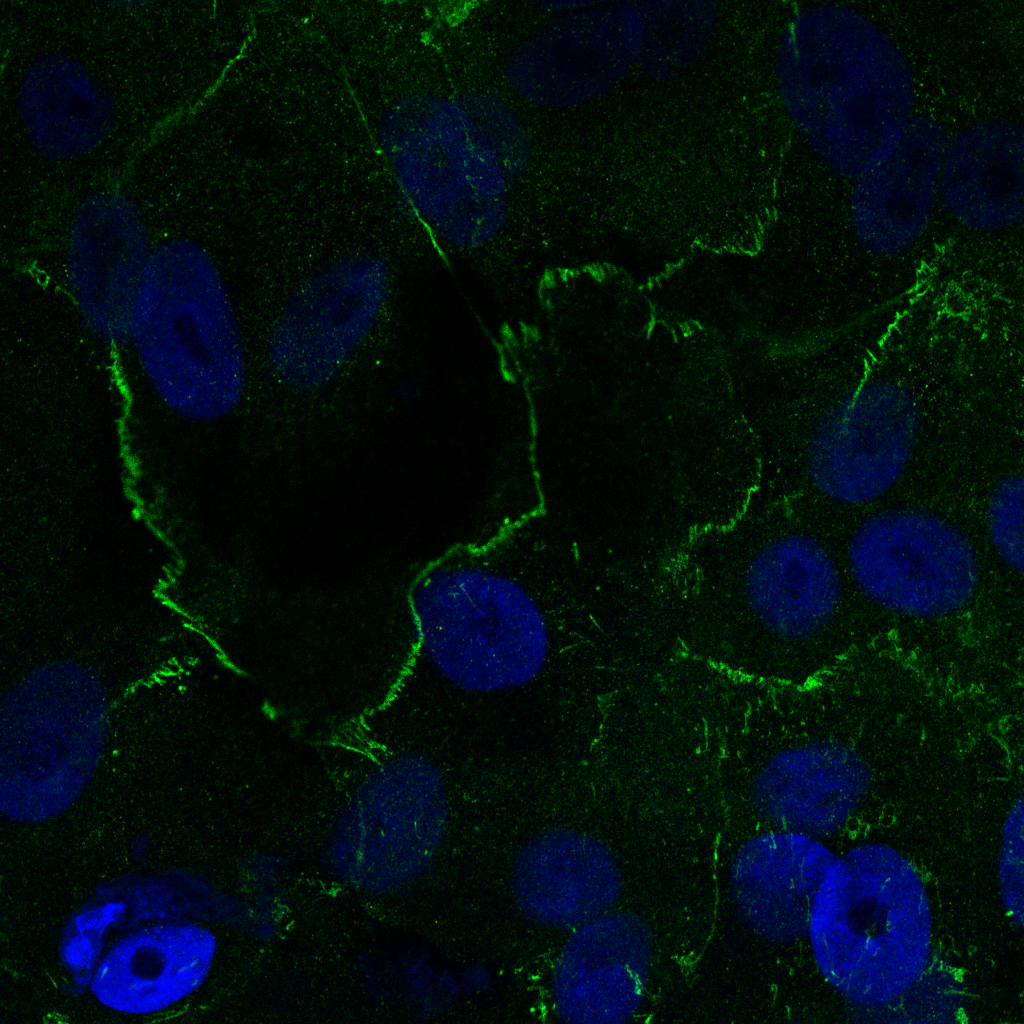

Supplement: Supplementary file 5 — Source data Fig. 2 [file 44321_2024_104_MOESM5_ESM.zip › Figure 2/2D/OCLN Veh/Veh OCLN Merge.tif]

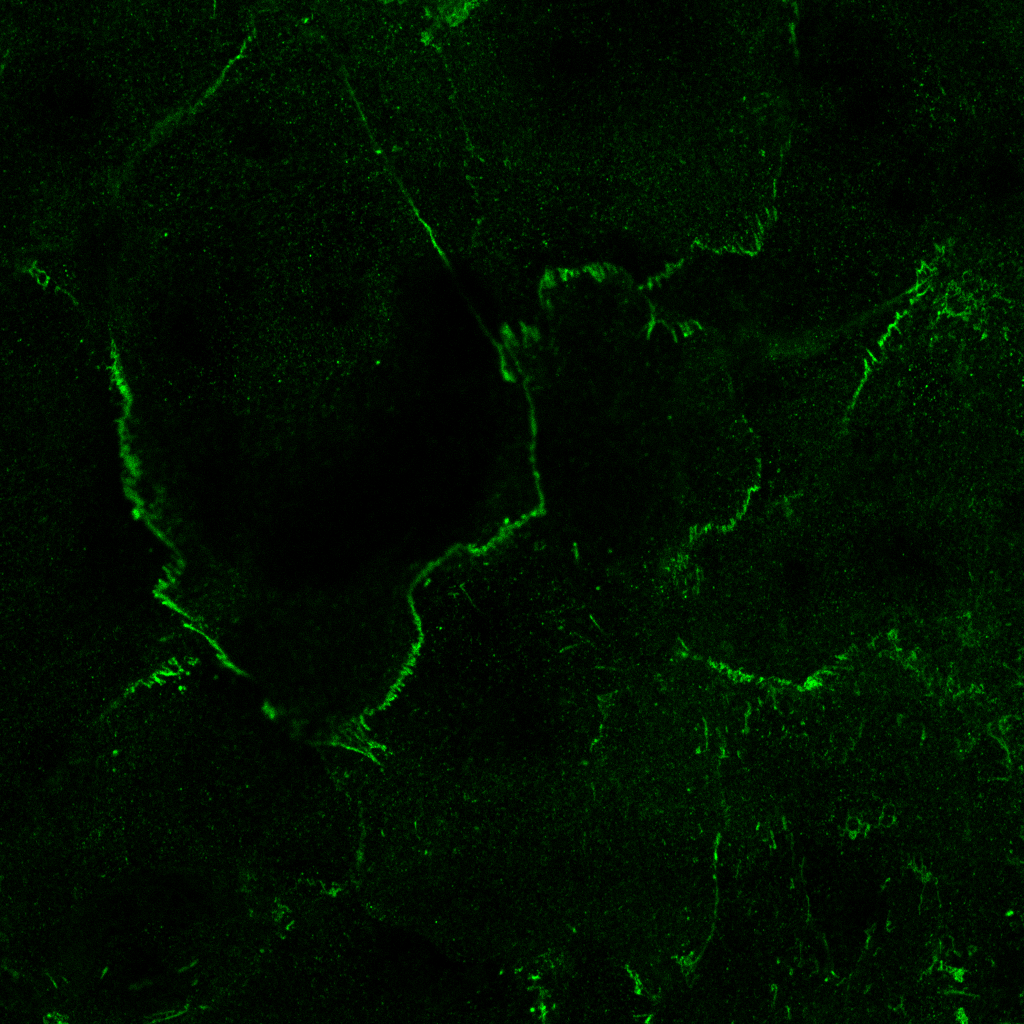

Supplement: Supplementary file 5 — Source data Fig. 2 [file 44321_2024_104_MOESM5_ESM.zip › Figure 2/2D/OCLN Veh/Veh OCLN.tif]

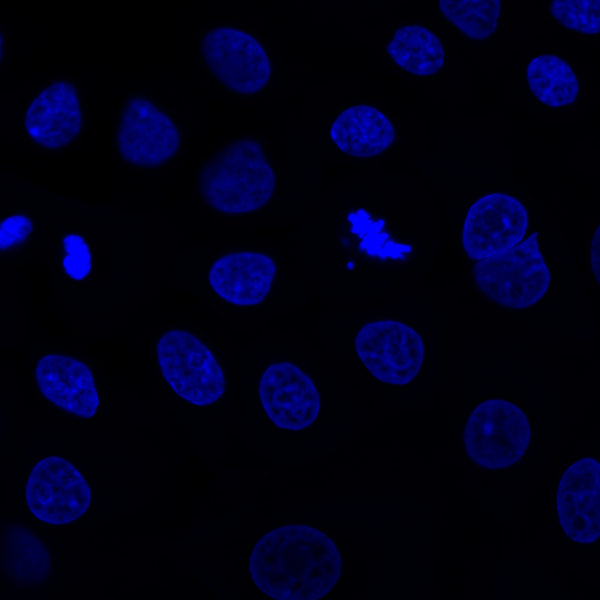

Supplement: Supplementary file 5 — Source data Fig. 2 [file 44321_2024_104_MOESM5_ESM.zip › Figure 2/2F/B-catenin siATP2A2 + Dl/siATP2A2 + Dl B-catenin DAPI.tif]

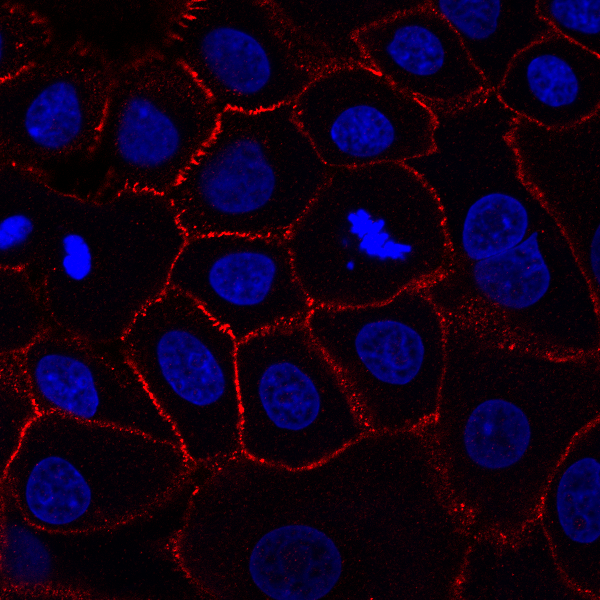

Supplement: Supplementary file 5 — Source data Fig. 2 [file 44321_2024_104_MOESM5_ESM.zip › Figure 2/2F/B-catenin siATP2A2 + Dl/siATP2A2 + Dl B-catenin Merge.tif]

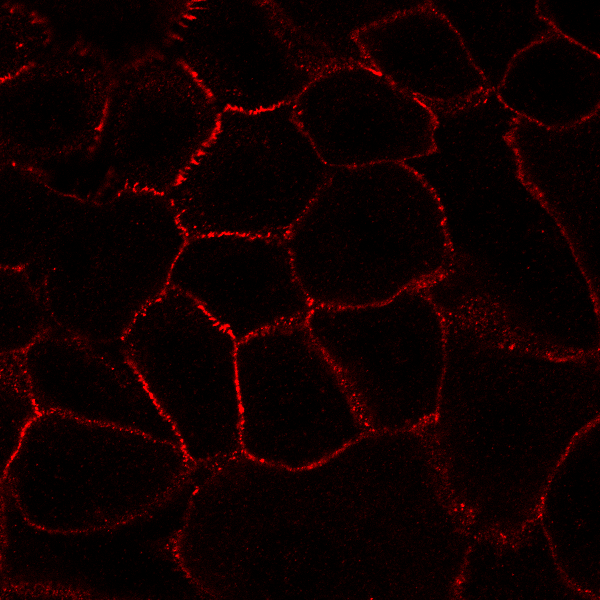

Supplement: Supplementary file 5 — Source data Fig. 2 [file 44321_2024_104_MOESM5_ESM.zip › Figure 2/2F/B-catenin siATP2A2 + Dl/siATP2A2 + Dl B-catenin.tif]

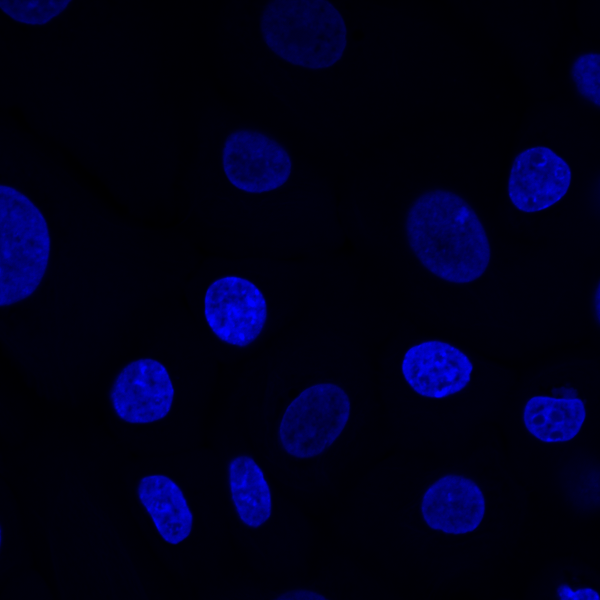

Supplement: Supplementary file 5 — Source data Fig. 2 [file 44321_2024_104_MOESM5_ESM.zip › Figure 2/2F/B-catenin siATP2A2/siATP2A2 B-cat DAPI.tif]

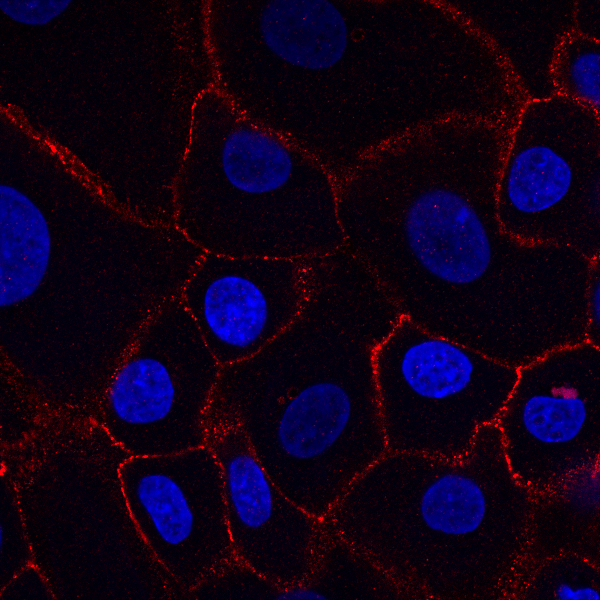

Supplement: Supplementary file 5 — Source data Fig. 2 [file 44321_2024_104_MOESM5_ESM.zip › Figure 2/2F/B-catenin siATP2A2/siATP2A2 B-cat Merge.tif]

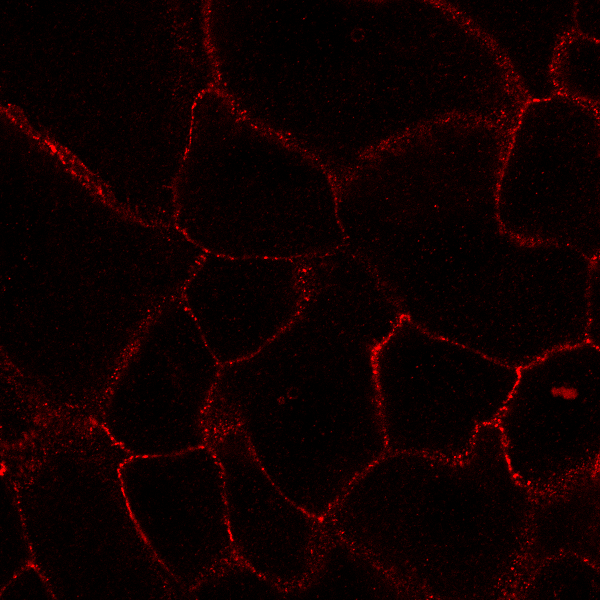

Supplement: Supplementary file 5 — Source data Fig. 2 [file 44321_2024_104_MOESM5_ESM.zip › Figure 2/2F/B-catenin siATP2A2/siATP2A2 B-cat.tif]

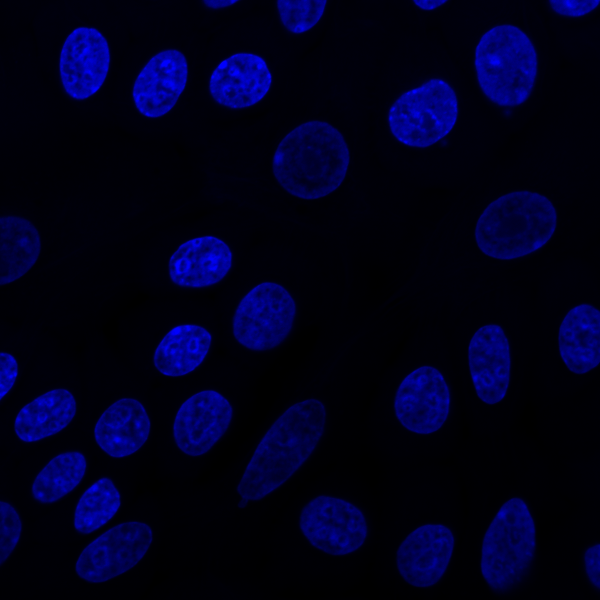

Supplement: Supplementary file 5 — Source data Fig. 2 [file 44321_2024_104_MOESM5_ESM.zip › Figure 2/2F/B-catenin siNEG + Dl/siNEG + Dl B-cat DAPI.tif]

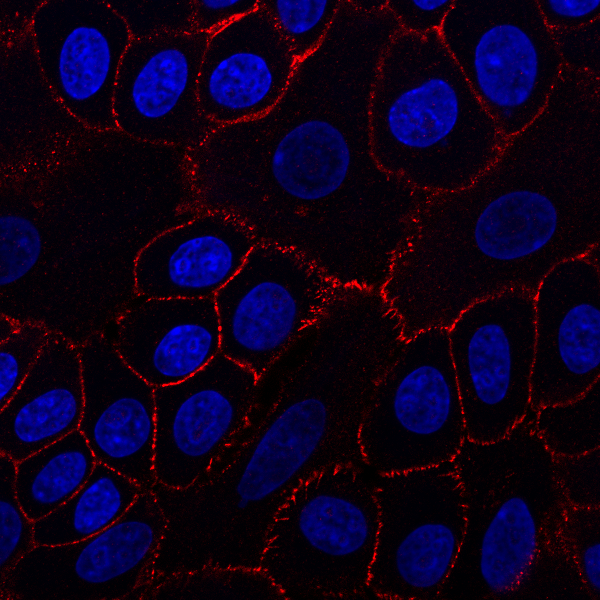

Supplement: Supplementary file 5 — Source data Fig. 2 [file 44321_2024_104_MOESM5_ESM.zip › Figure 2/2F/B-catenin siNEG + Dl/siNEG + Dl B-cat Merge.tif]

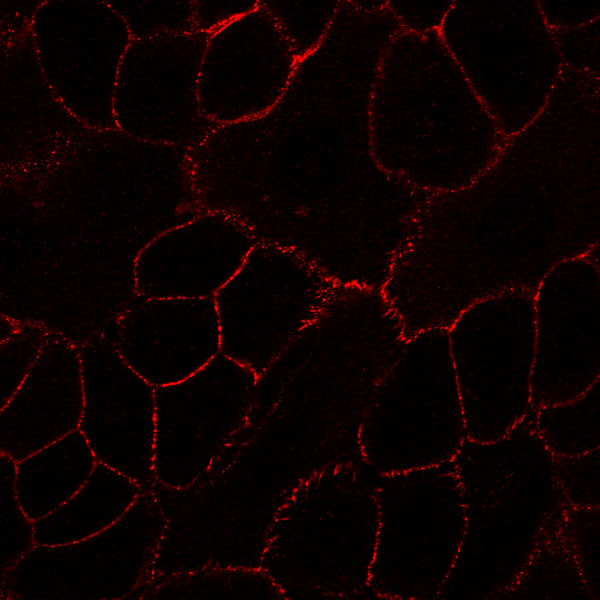

Supplement: Supplementary file 5 — Source data Fig. 2 [file 44321_2024_104_MOESM5_ESM.zip › Figure 2/2F/B-catenin siNEG + Dl/siNEG + Dl B-cat.tif]

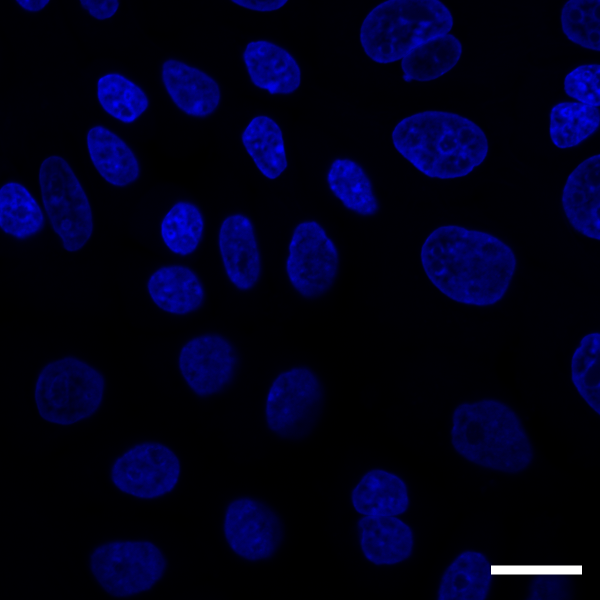

Supplement: Supplementary file 5 — Source data Fig. 2 [file 44321_2024_104_MOESM5_ESM.zip › Figure 2/2F/B-catenin siNEG/siNEG B-cat DAPI.tif]

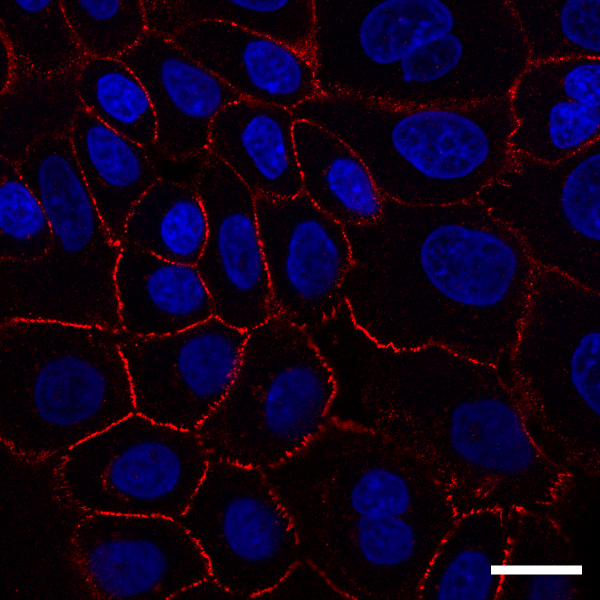

Supplement: Supplementary file 5 — Source data Fig. 2 [file 44321_2024_104_MOESM5_ESM.zip › Figure 2/2F/B-catenin siNEG/siNEG B-cat Merge.tif]

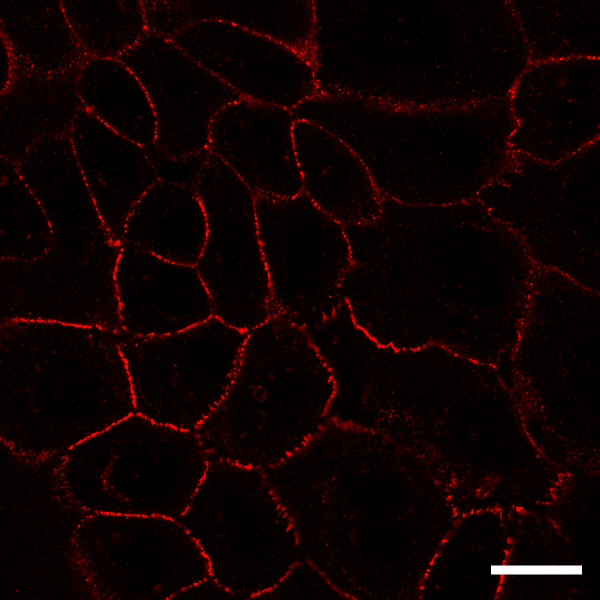

Supplement: Supplementary file 5 — Source data Fig. 2 [file 44321_2024_104_MOESM5_ESM.zip › Figure 2/2F/B-catenin siNEG/siNEG B-cat.tif]

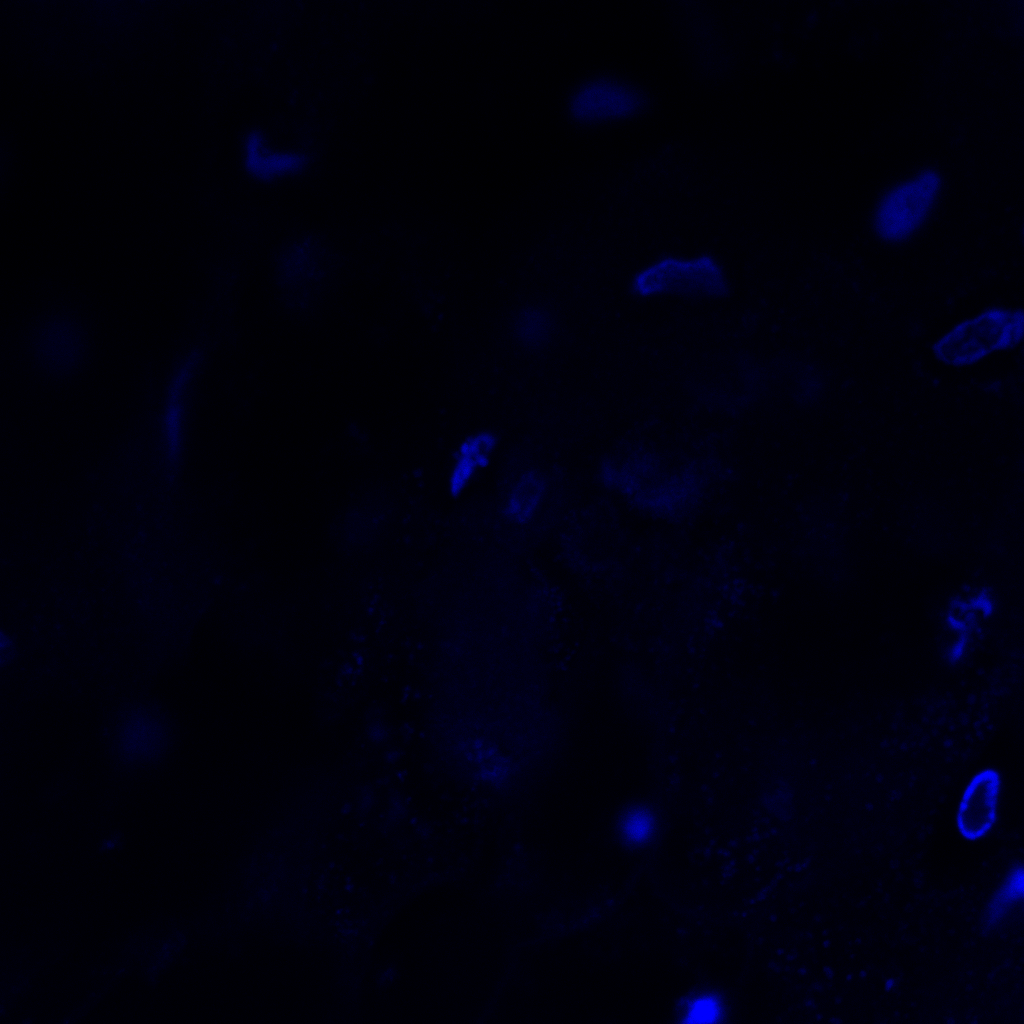

Supplement: Supplementary file 5 — Source data Fig. 2 [file 44321_2024_104_MOESM5_ESM.zip › Figure 2/2F/DSG1 siATP2A2 + Dl/siATP2A2 + Dl DSG1 DAPI.tif]

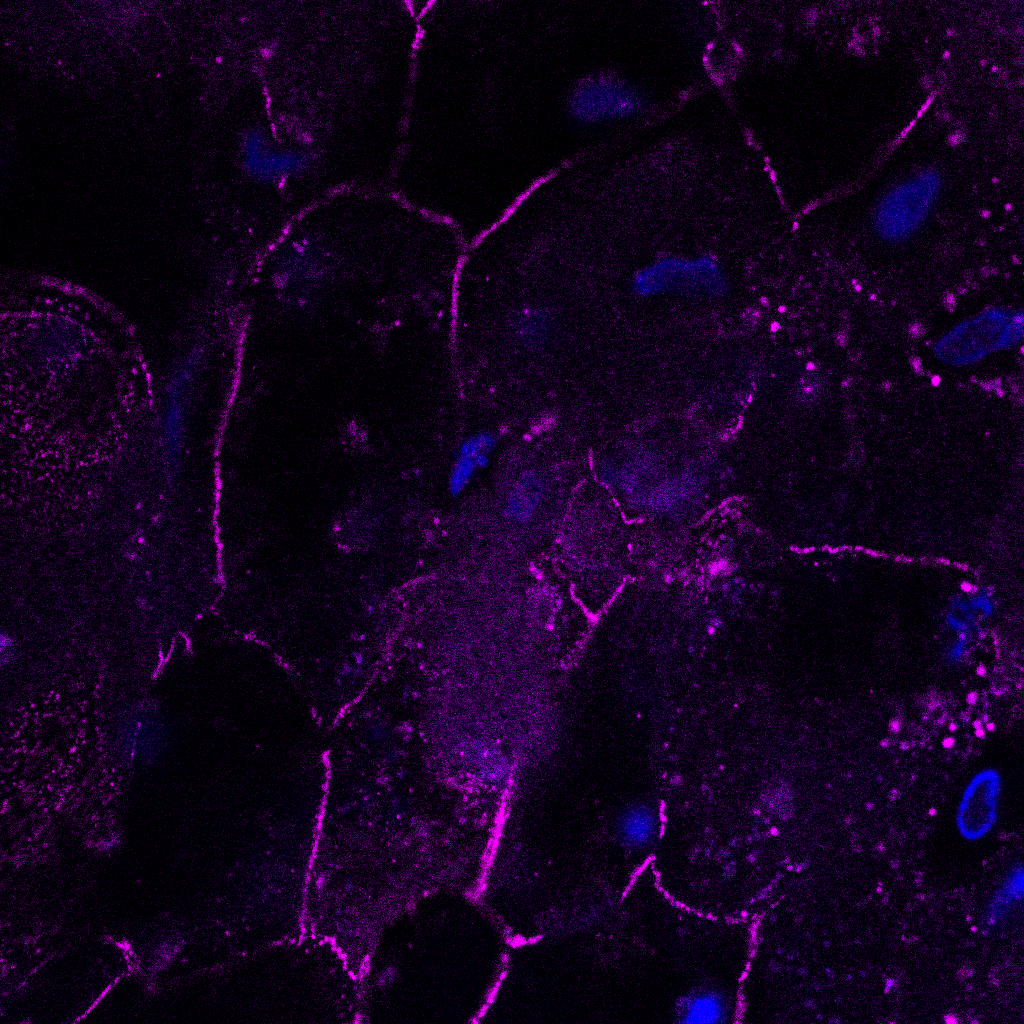

Supplement: Supplementary file 5 — Source data Fig. 2 [file 44321_2024_104_MOESM5_ESM.zip › Figure 2/2F/DSG1 siATP2A2 + Dl/siATP2A2 + Dl DSG1 Merge.tif]

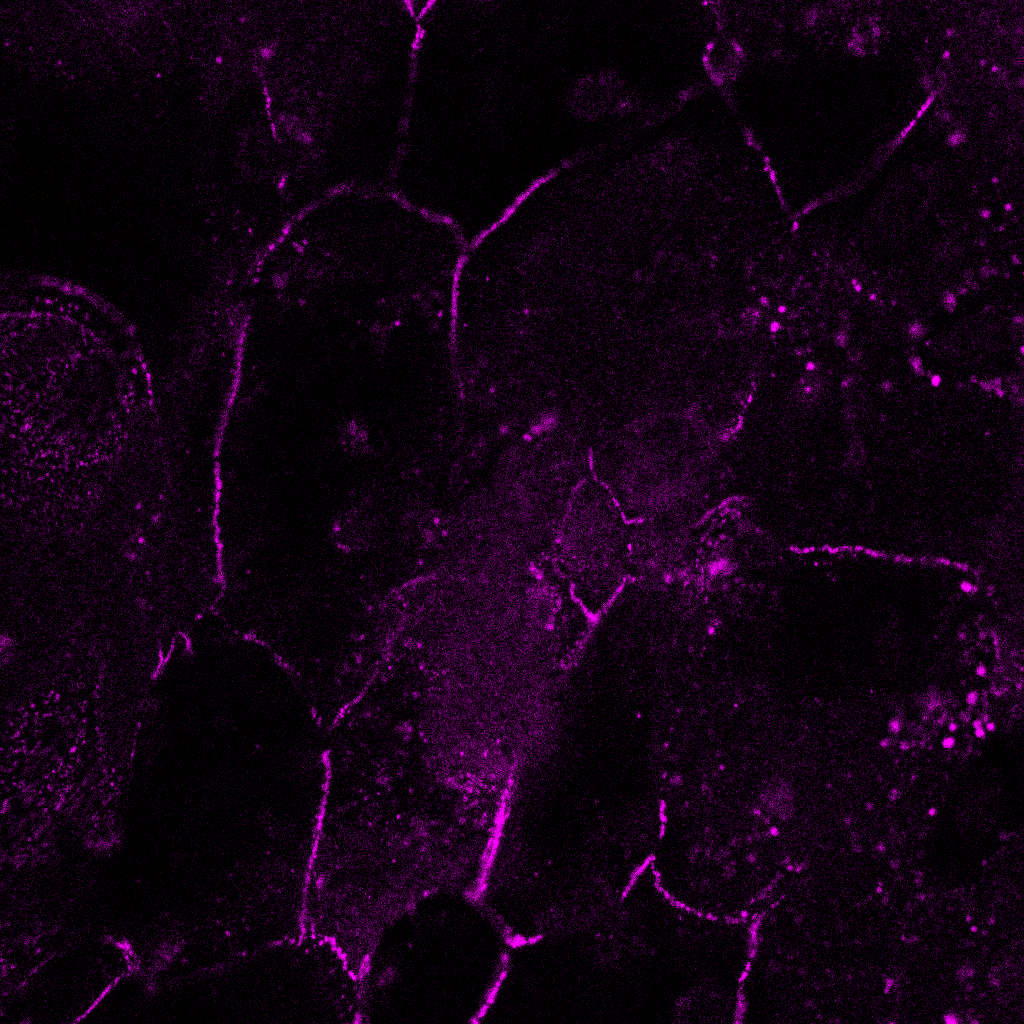

Supplement: Supplementary file 5 — Source data Fig. 2 [file 44321_2024_104_MOESM5_ESM.zip › Figure 2/2F/DSG1 siATP2A2 + Dl/siATP2A2 + Dl DSG1.tif]

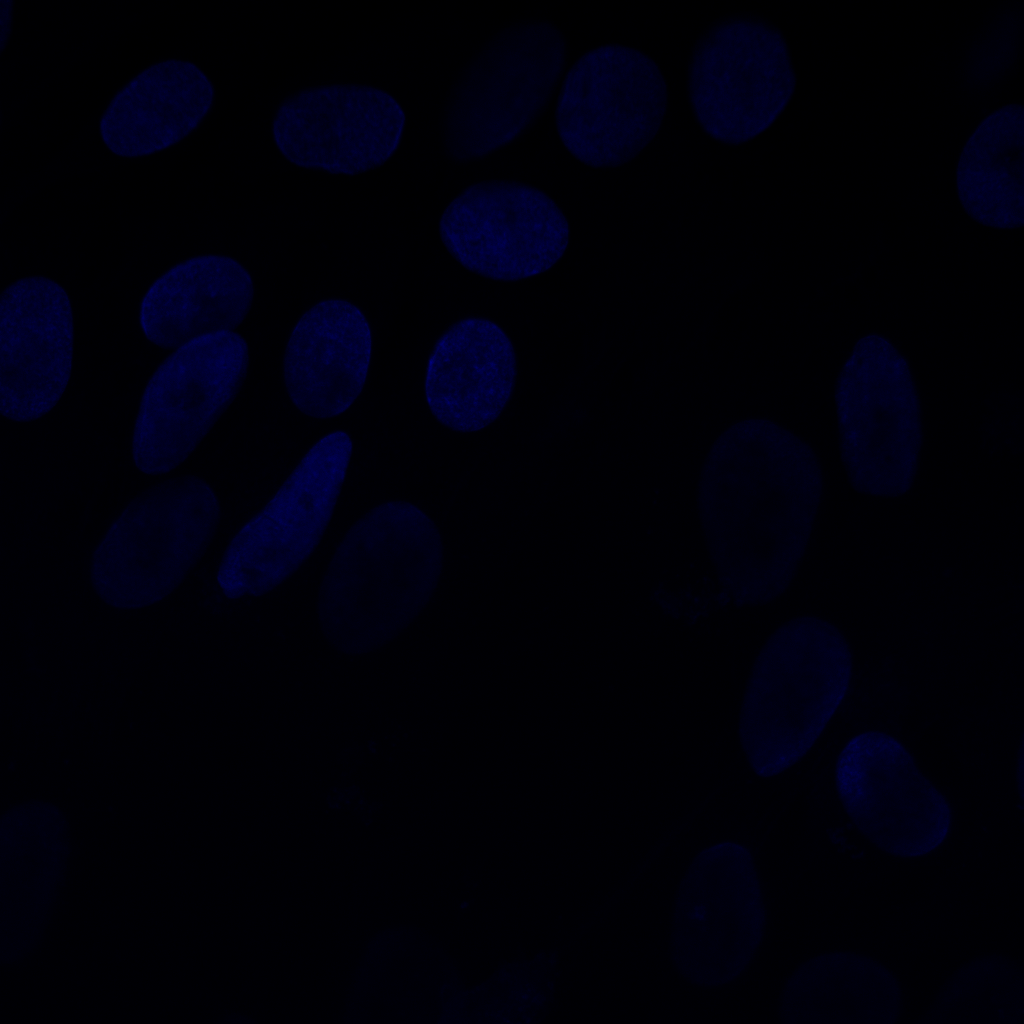

Supplement: Supplementary file 5 — Source data Fig. 2 [file 44321_2024_104_MOESM5_ESM.zip › Figure 2/2F/DSG1 siATP2A2/siATP2A2 DSG1 DAPI.tif]

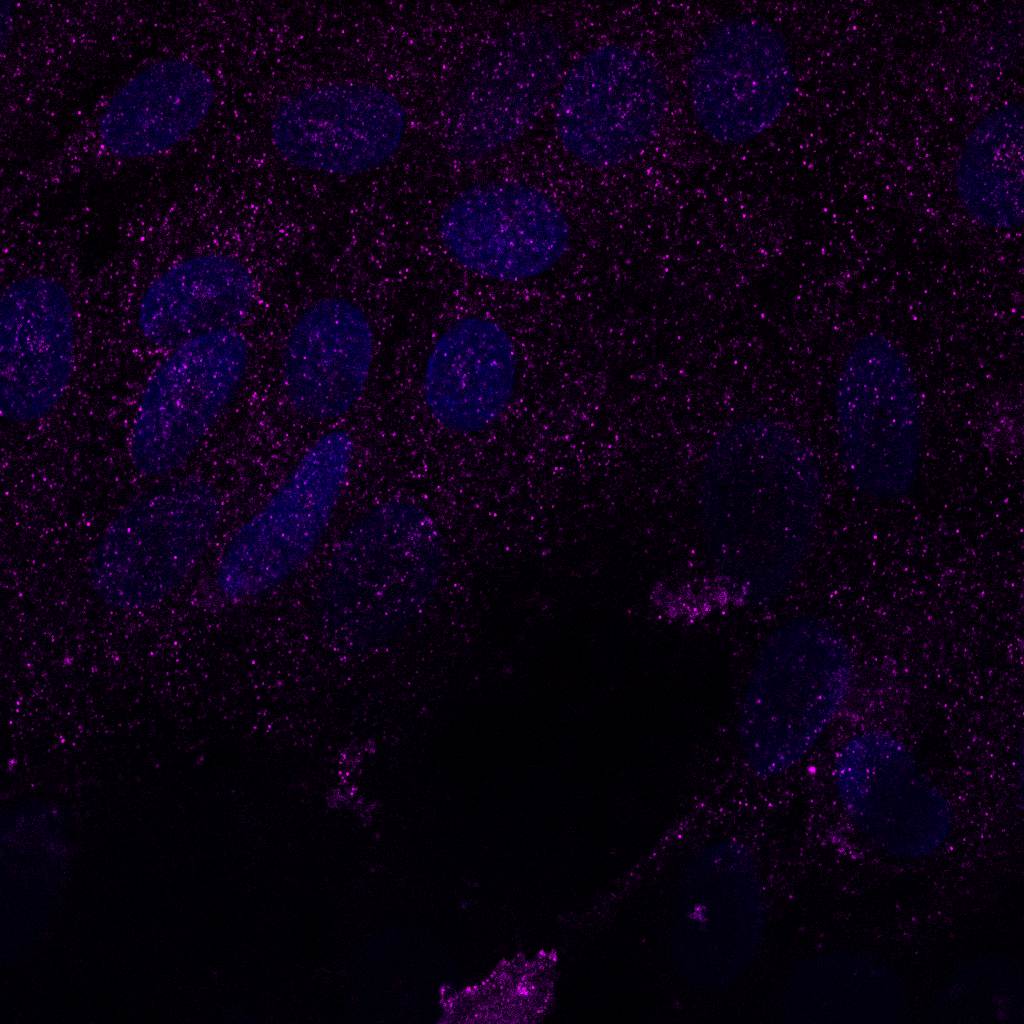

Supplement: Supplementary file 5 — Source data Fig. 2 [file 44321_2024_104_MOESM5_ESM.zip › Figure 2/2F/DSG1 siATP2A2/siATP2A2 DSG1 Merge.tif]

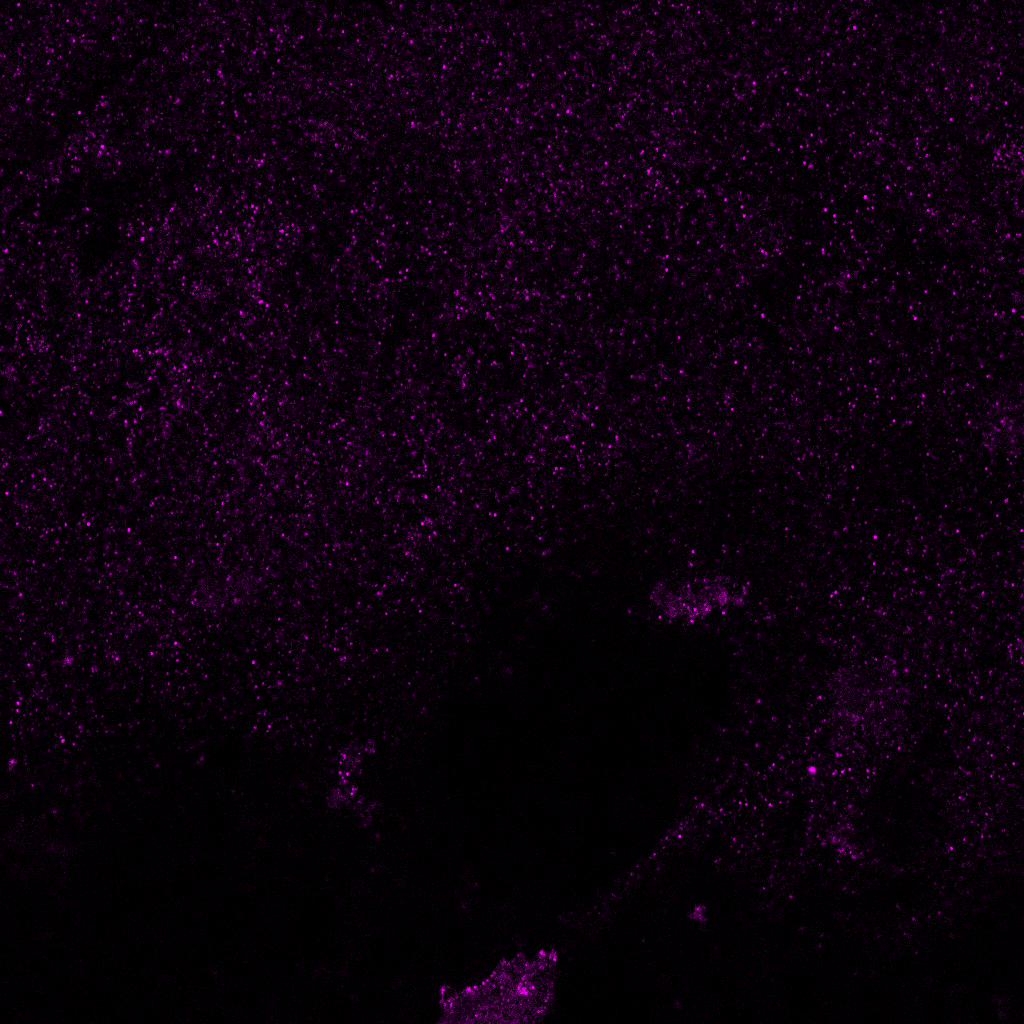

Supplement: Supplementary file 5 — Source data Fig. 2 [file 44321_2024_104_MOESM5_ESM.zip › Figure 2/2F/DSG1 siATP2A2/siATP2A2 DSG1.tif]

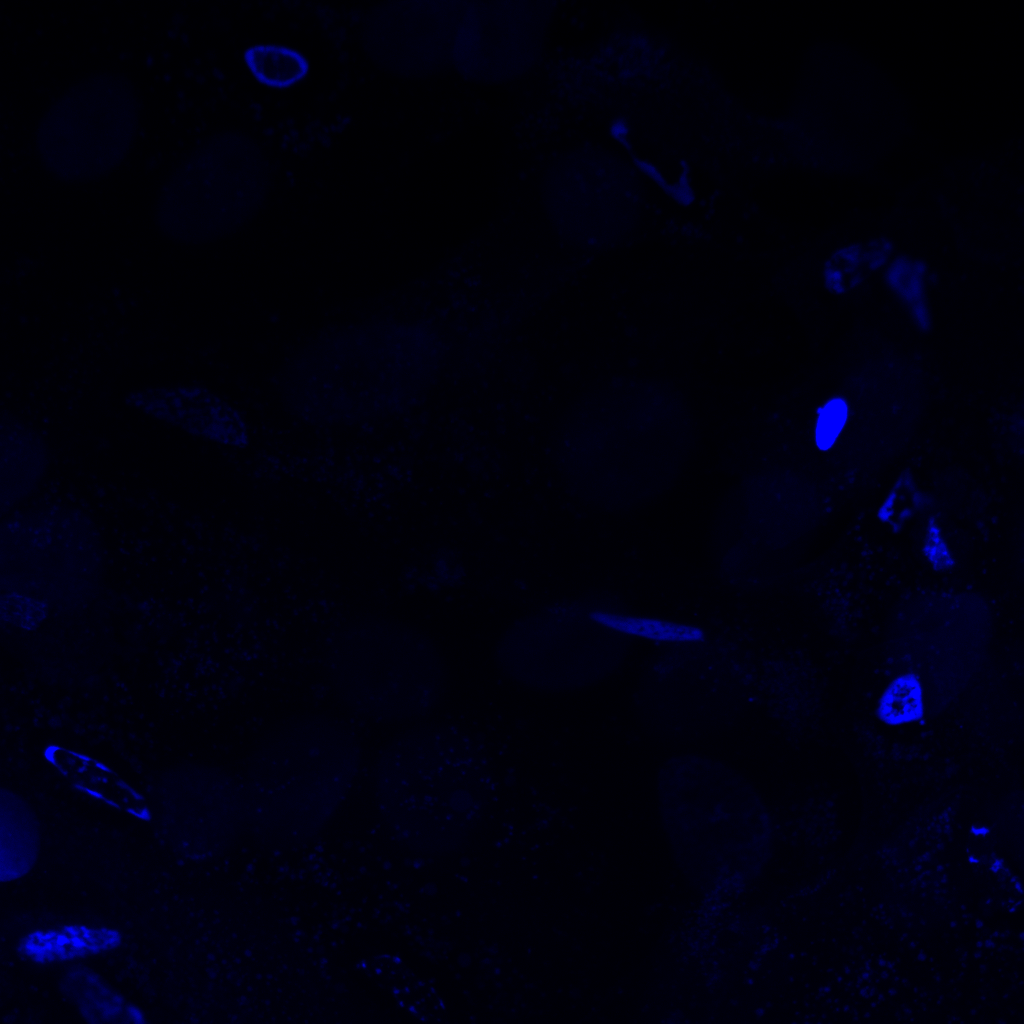

Supplement: Supplementary file 5 — Source data Fig. 2 [file 44321_2024_104_MOESM5_ESM.zip › Figure 2/2F/DSG1 siNEG + Dl/siNEG + Dl DSG1 DAPI.tif]

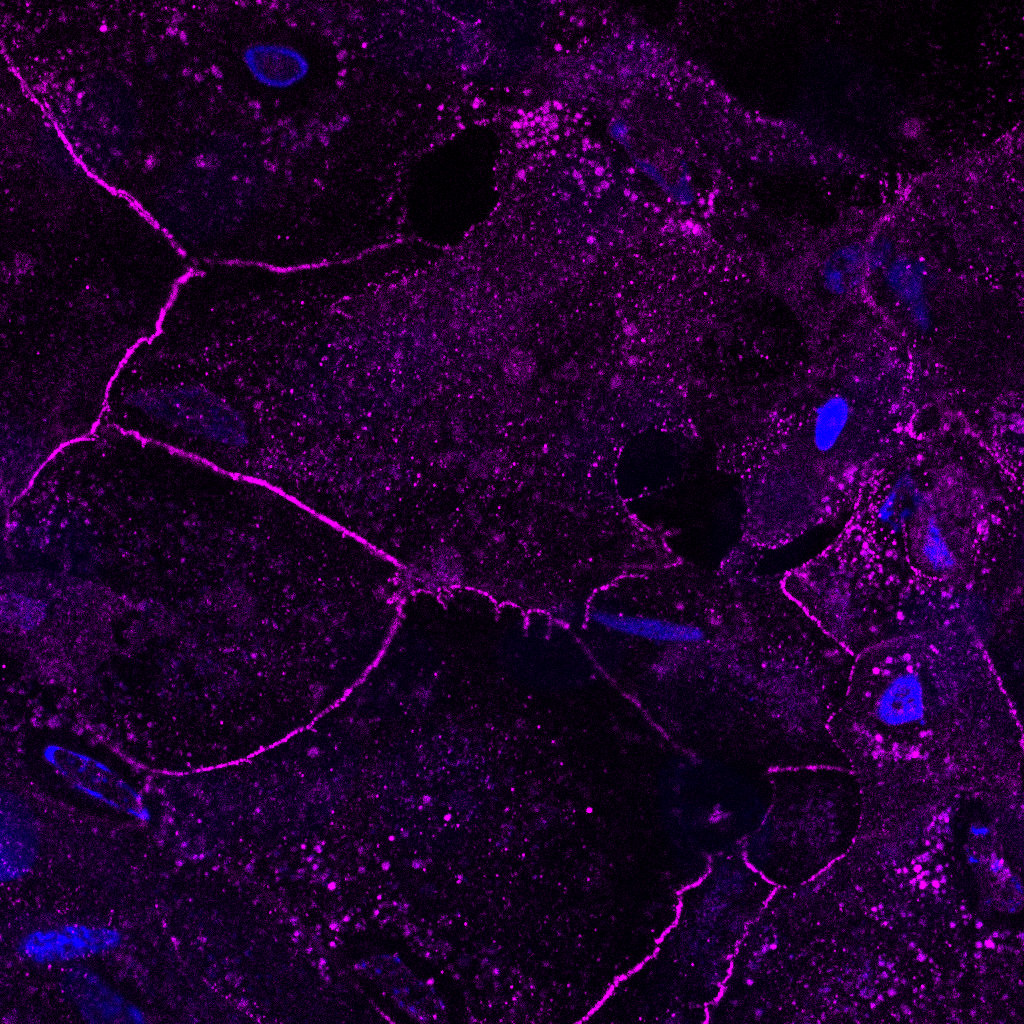

Supplement: Supplementary file 5 — Source data Fig. 2 [file 44321_2024_104_MOESM5_ESM.zip › Figure 2/2F/DSG1 siNEG + Dl/siNEG + Dl DSG1 Merge.tif]

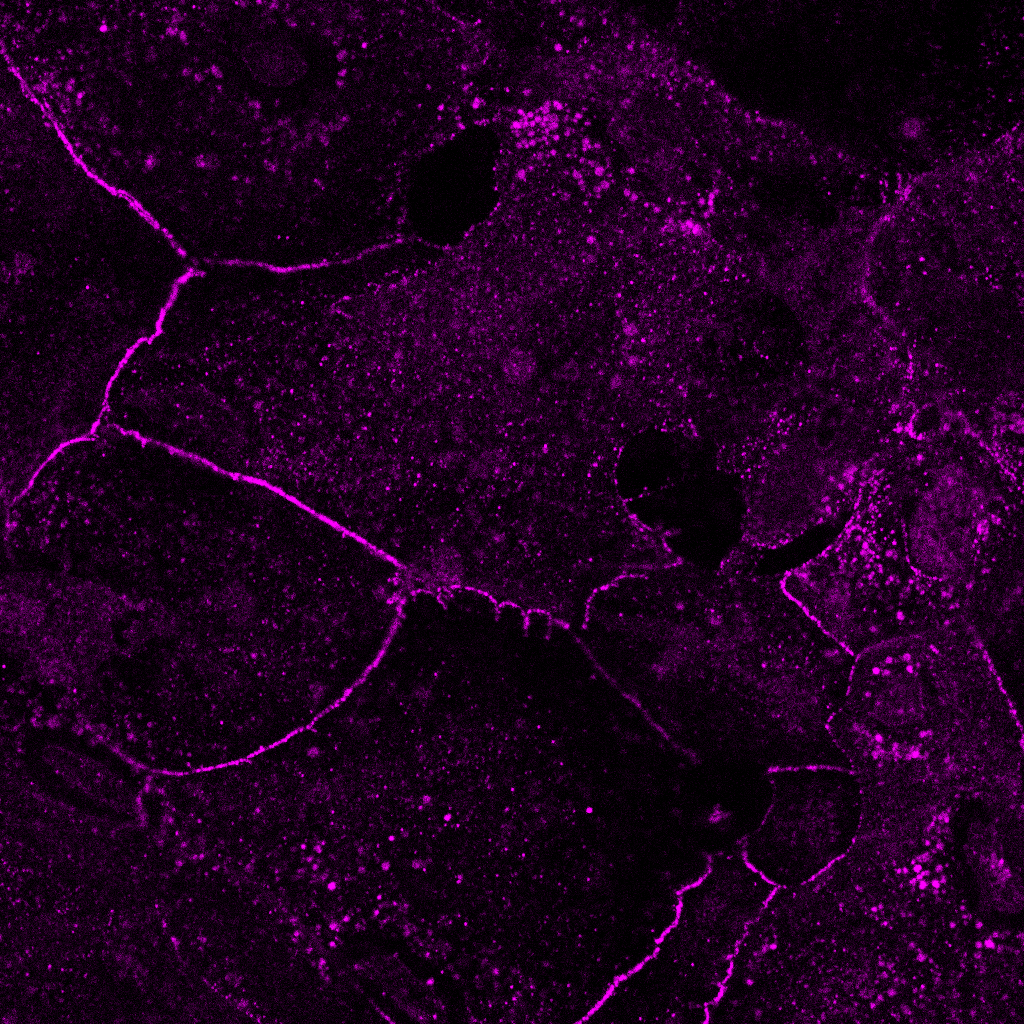

Supplement: Supplementary file 5 — Source data Fig. 2 [file 44321_2024_104_MOESM5_ESM.zip › Figure 2/2F/DSG1 siNEG + Dl/siNEG + Dl DSG1.tif]

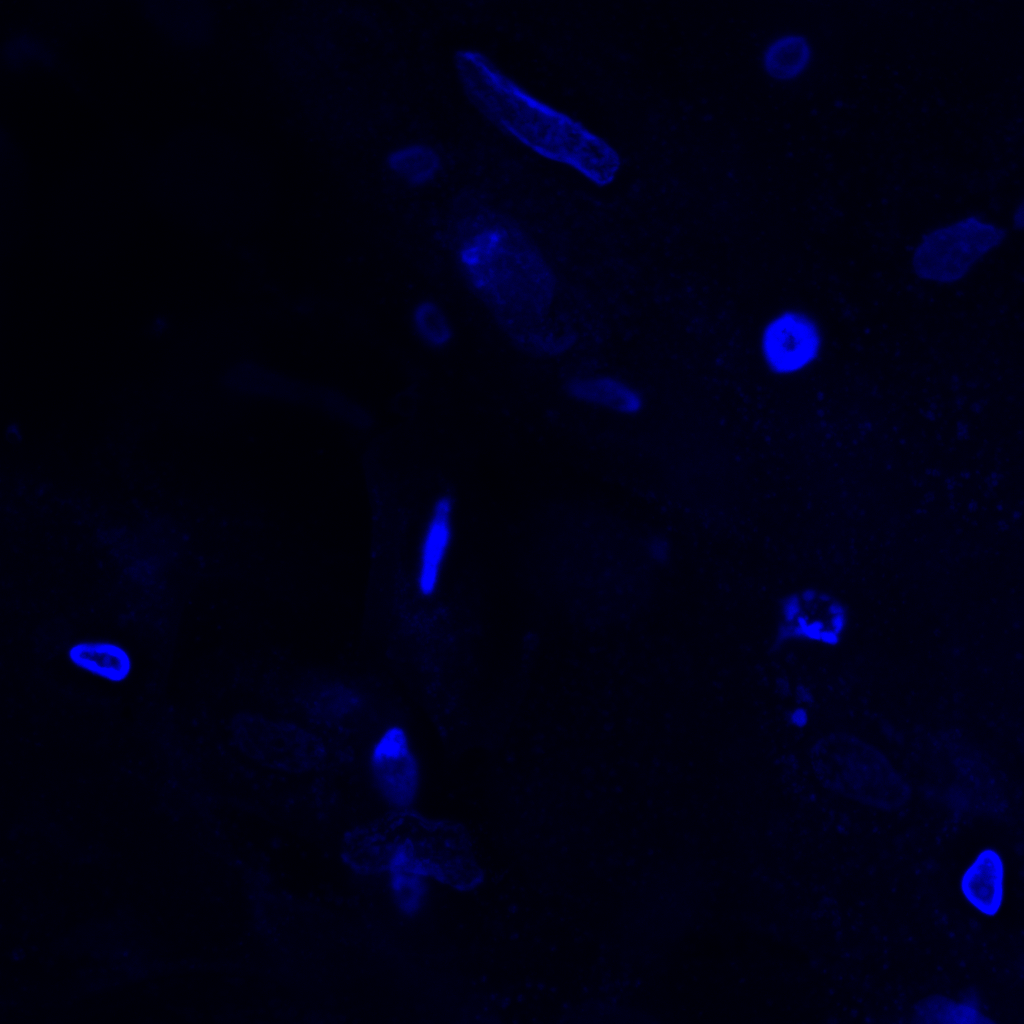

Supplement: Supplementary file 5 — Source data Fig. 2 [file 44321_2024_104_MOESM5_ESM.zip › Figure 2/2F/DSG1 siNEG/siNEG DSG1 DAPI.tif]

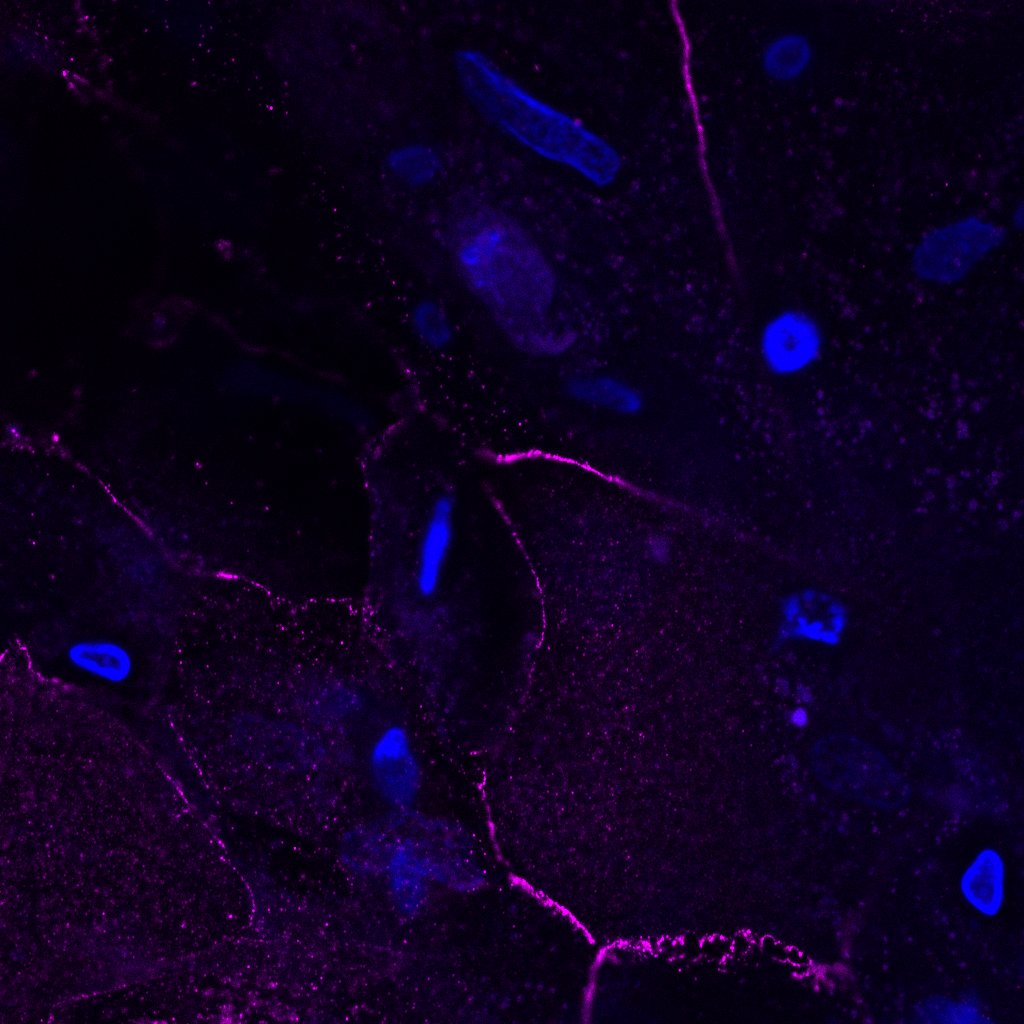

Supplement: Supplementary file 5 — Source data Fig. 2 [file 44321_2024_104_MOESM5_ESM.zip › Figure 2/2F/DSG1 siNEG/siNEG DSG1 Merge.tif]

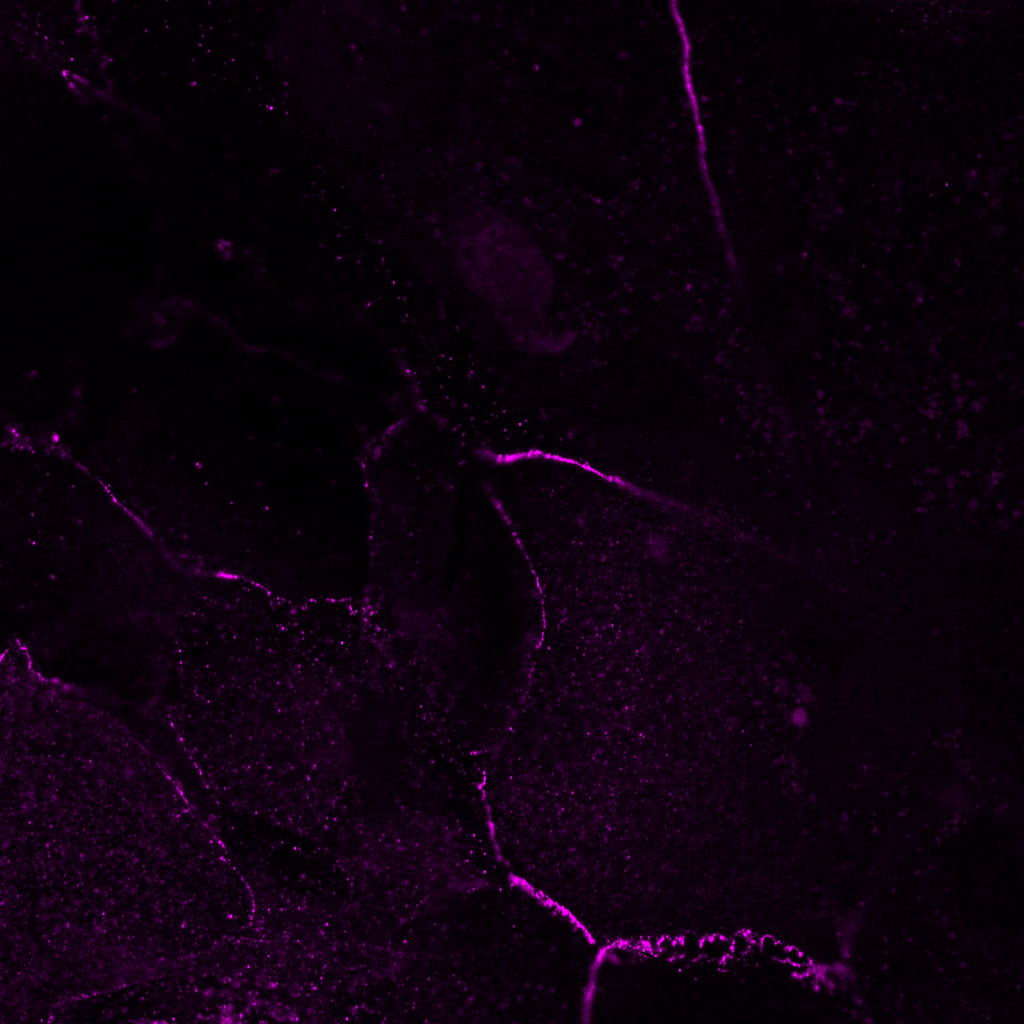

Supplement: Supplementary file 5 — Source data Fig. 2 [file 44321_2024_104_MOESM5_ESM.zip › Figure 2/2F/DSG1 siNEG/siNEG DSG1.tif]

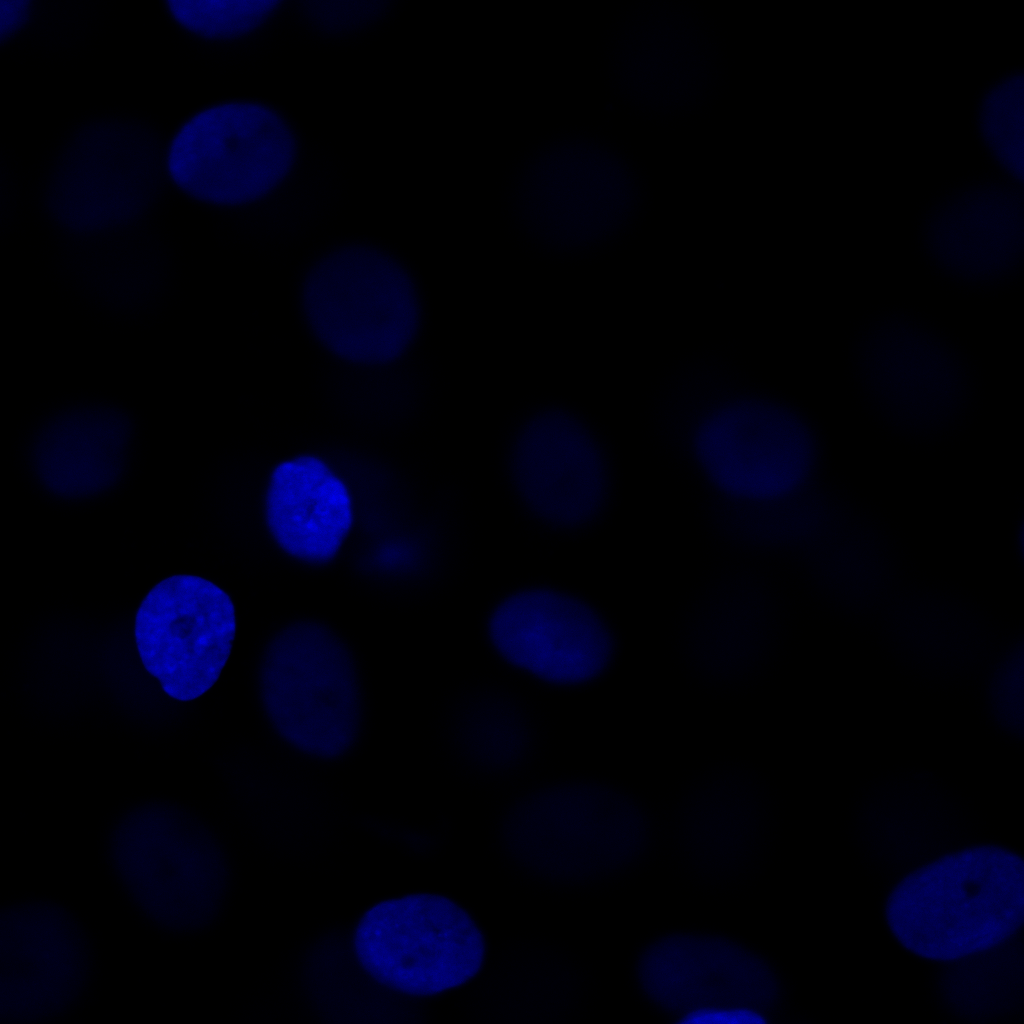

Supplement: Supplementary file 5 — Source data Fig. 2 [file 44321_2024_104_MOESM5_ESM.zip › Figure 2/2F/OCLN siATP2A2 + Dl/siATP2A2 + Dl OCLN DAPI.tif]

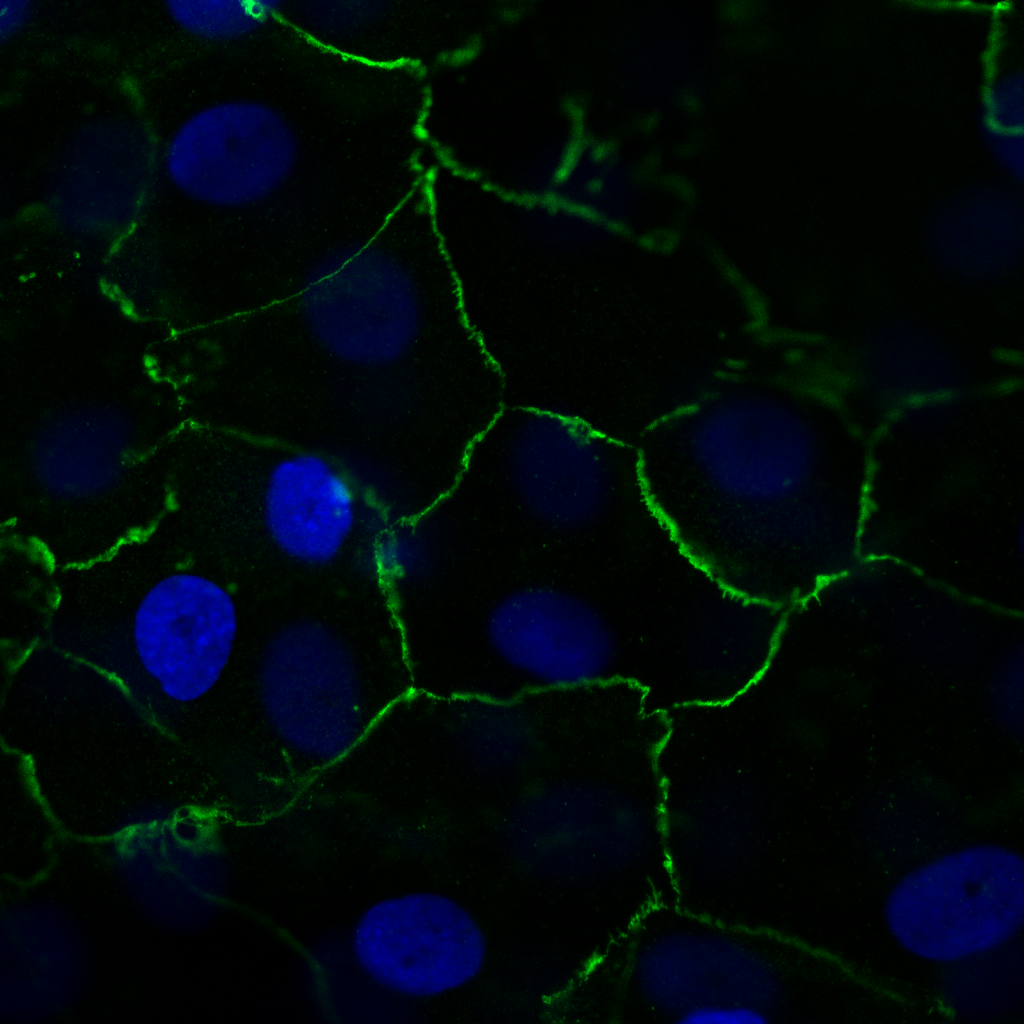

Supplement: Supplementary file 5 — Source data Fig. 2 [file 44321_2024_104_MOESM5_ESM.zip › Figure 2/2F/OCLN siATP2A2 + Dl/siATP2A2 + Dl OCLN Merge.tif]

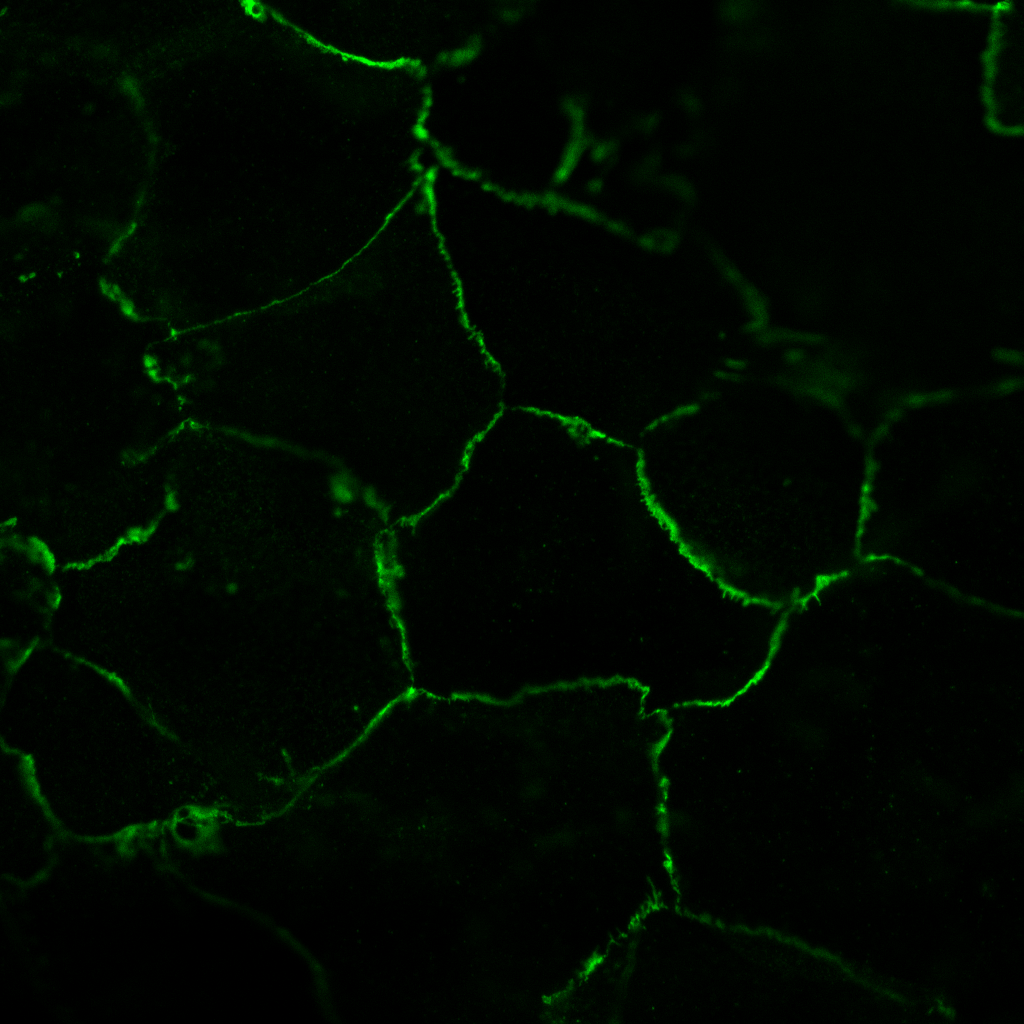

Supplement: Supplementary file 5 — Source data Fig. 2 [file 44321_2024_104_MOESM5_ESM.zip › Figure 2/2F/OCLN siATP2A2 + Dl/siATP2A2 + Dl OCLN.tif]

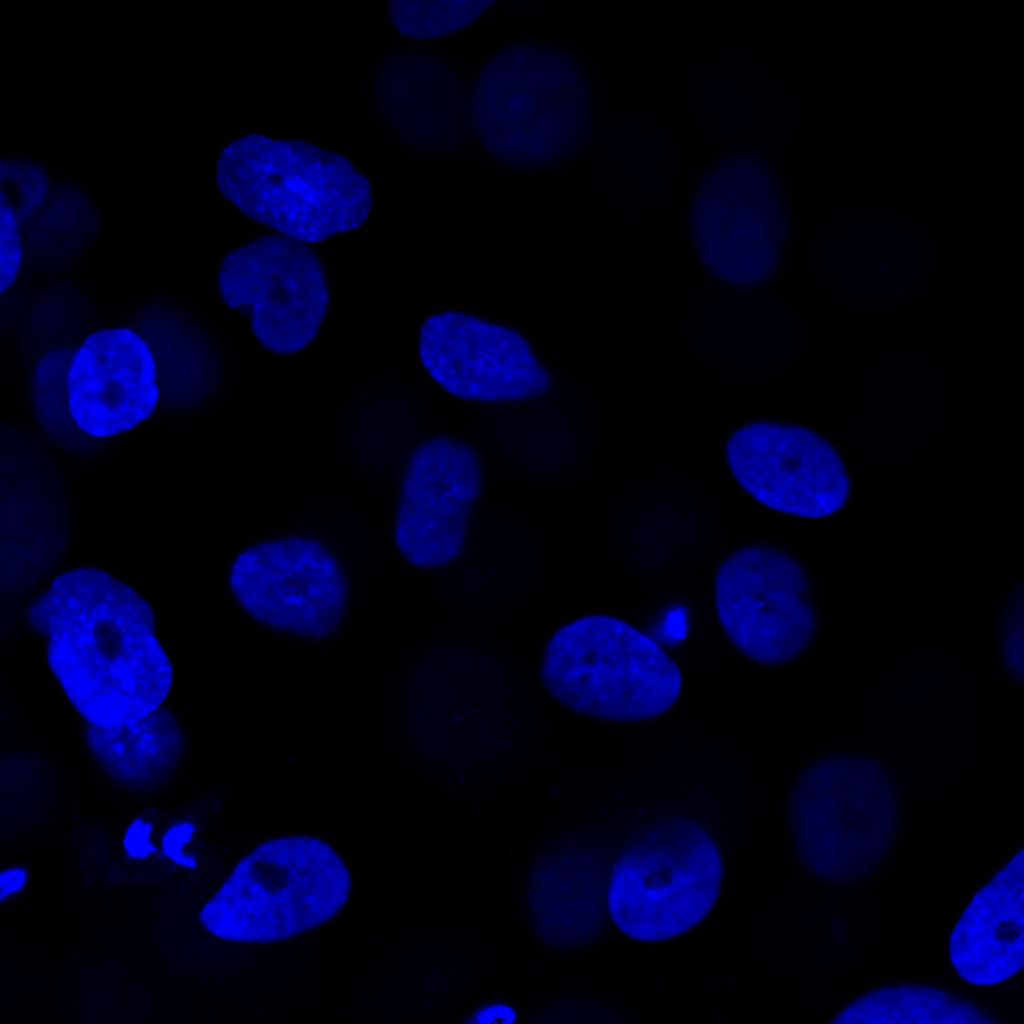

Supplement: Supplementary file 5 — Source data Fig. 2 [file 44321_2024_104_MOESM5_ESM.zip › Figure 2/2F/OCLN siATP2A2/siATP2A2 OCLN DAPI.tif]

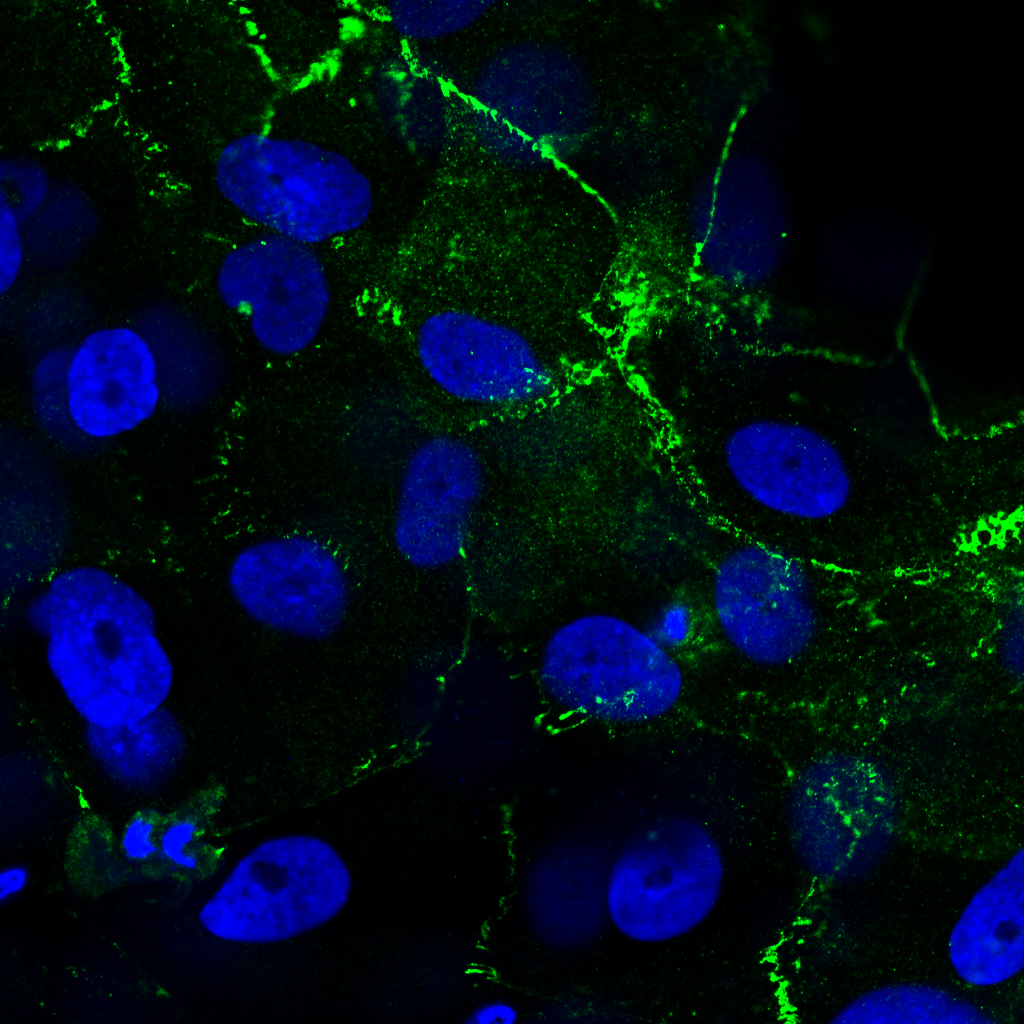

Supplement: Supplementary file 5 — Source data Fig. 2 [file 44321_2024_104_MOESM5_ESM.zip › Figure 2/2F/OCLN siATP2A2/siATP2A2 OCLN Merge.tif]

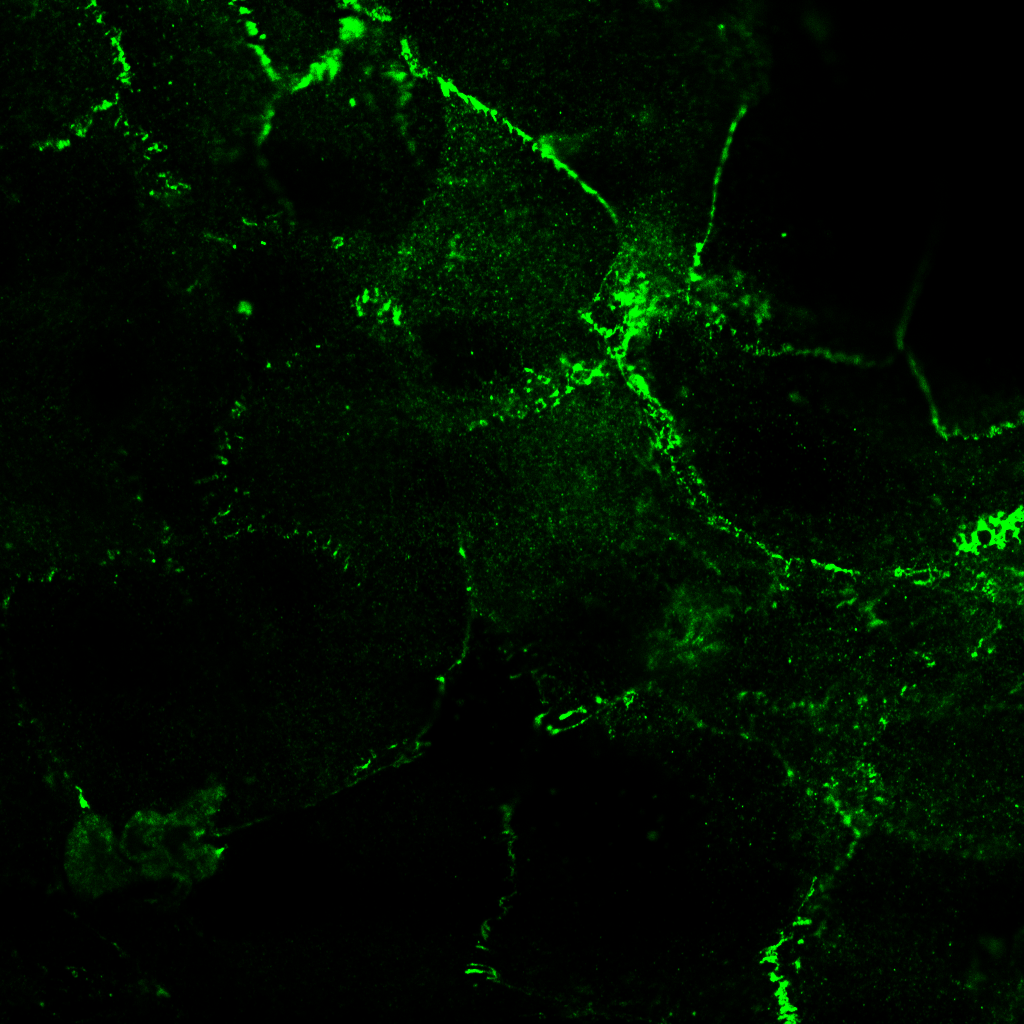

Supplement: Supplementary file 5 — Source data Fig. 2 [file 44321_2024_104_MOESM5_ESM.zip › Figure 2/2F/OCLN siATP2A2/siATP2A2 OCLN.tif]

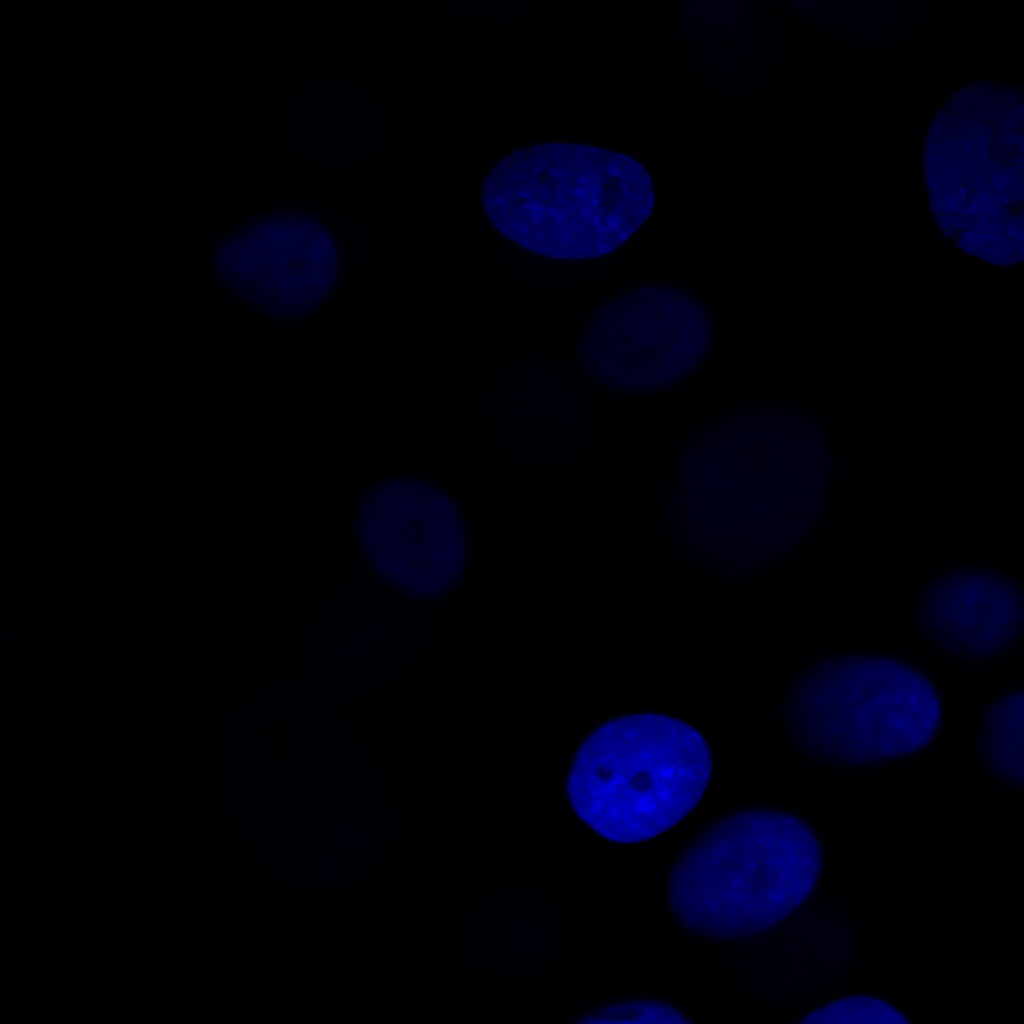

Supplement: Supplementary file 5 — Source data Fig. 2 [file 44321_2024_104_MOESM5_ESM.zip › Figure 2/2F/OCLN siNEG + Dl/siNEG + Dl OCLN DAPI.tif]

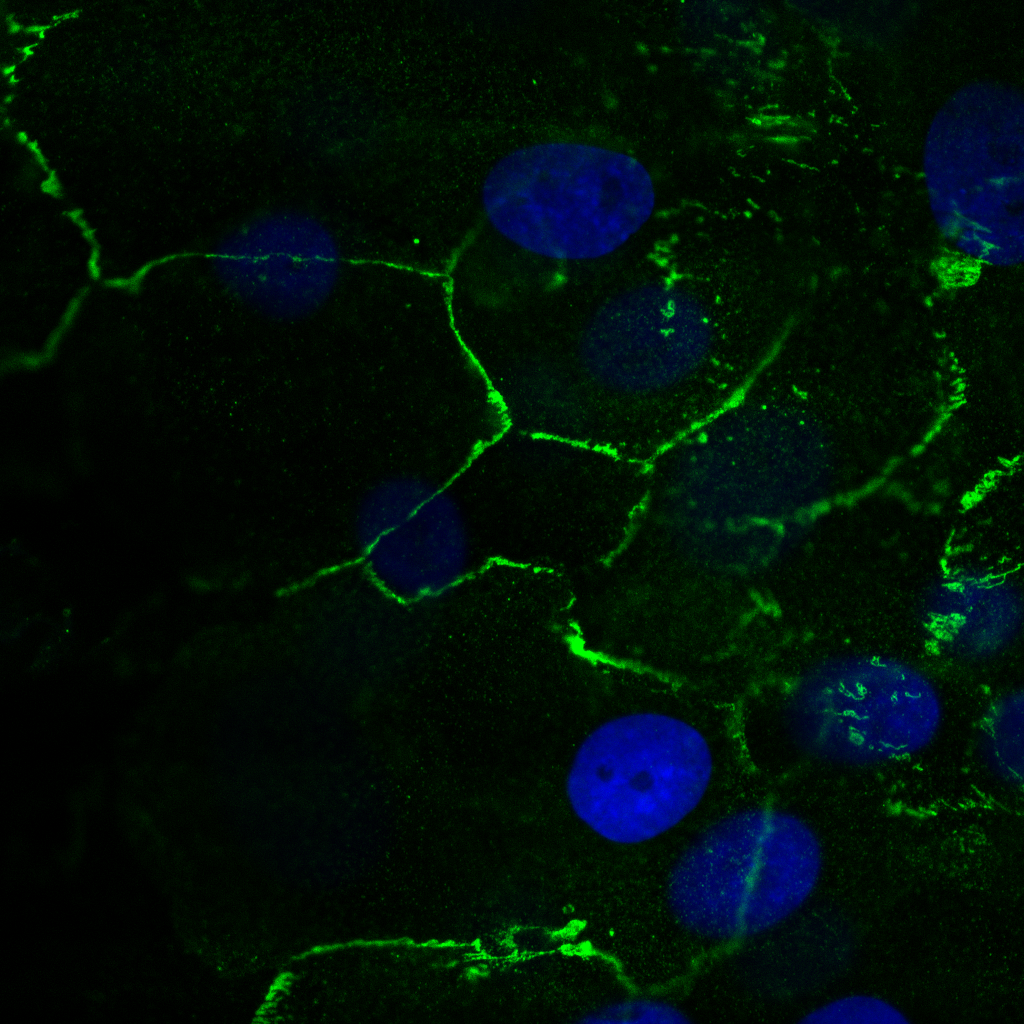

Supplement: Supplementary file 5 — Source data Fig. 2 [file 44321_2024_104_MOESM5_ESM.zip › Figure 2/2F/OCLN siNEG + Dl/siNEG + Dl OCLN Merge.tif]

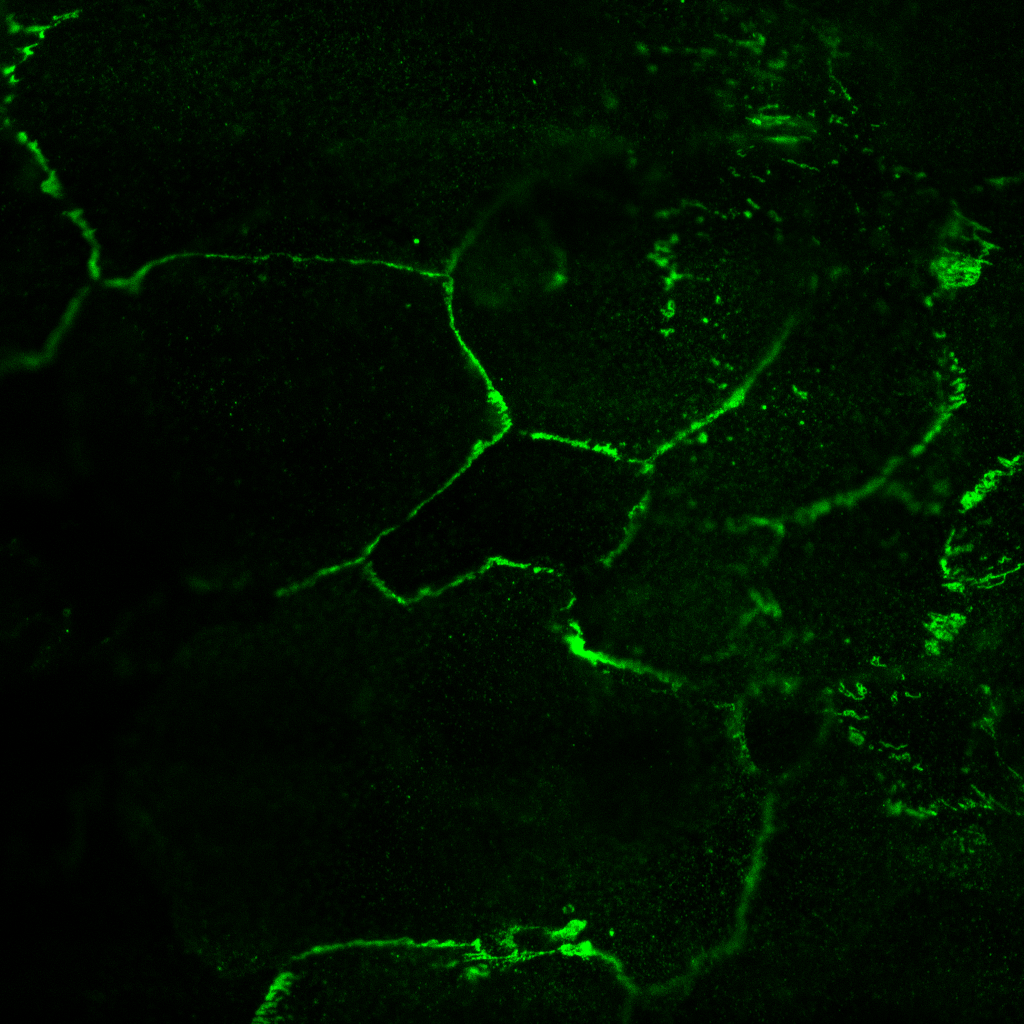

Supplement: Supplementary file 5 — Source data Fig. 2 [file 44321_2024_104_MOESM5_ESM.zip › Figure 2/2F/OCLN siNEG + Dl/siNEG + Dl OCLN.tif]

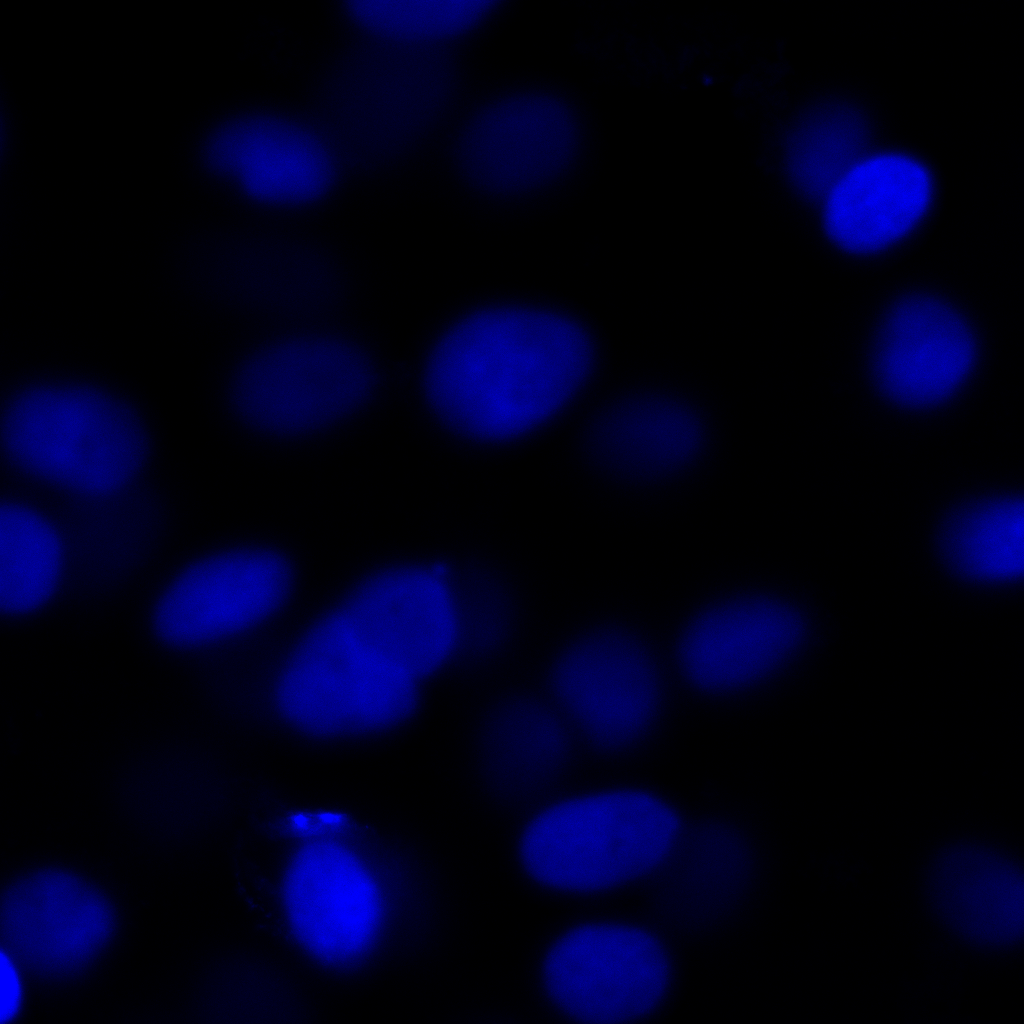

Supplement: Supplementary file 5 — Source data Fig. 2 [file 44321_2024_104_MOESM5_ESM.zip › Figure 2/2F/OCLN siNEG/siNEG OCLN DAPI.tif]

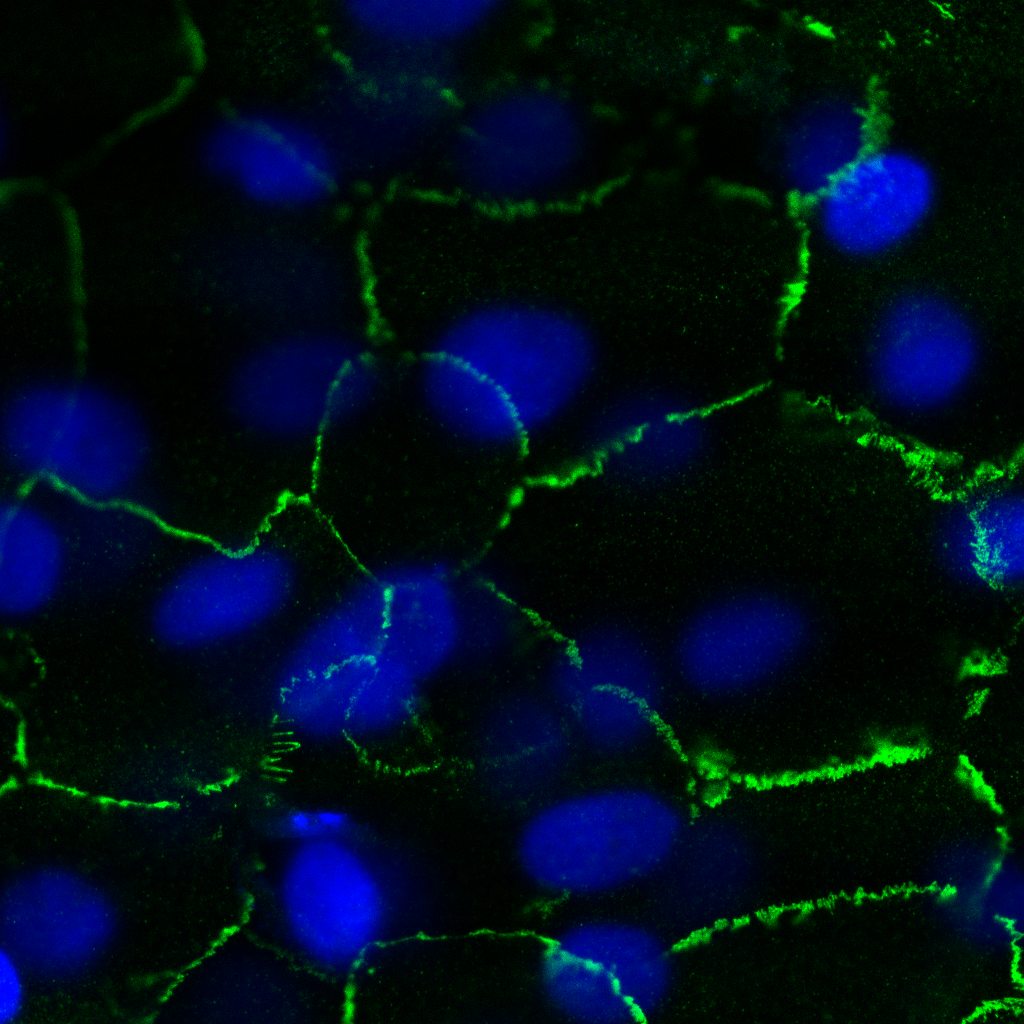

Supplement: Supplementary file 5 — Source data Fig. 2 [file 44321_2024_104_MOESM5_ESM.zip › Figure 2/2F/OCLN siNEG/siNEG OCLN Merge.tif]

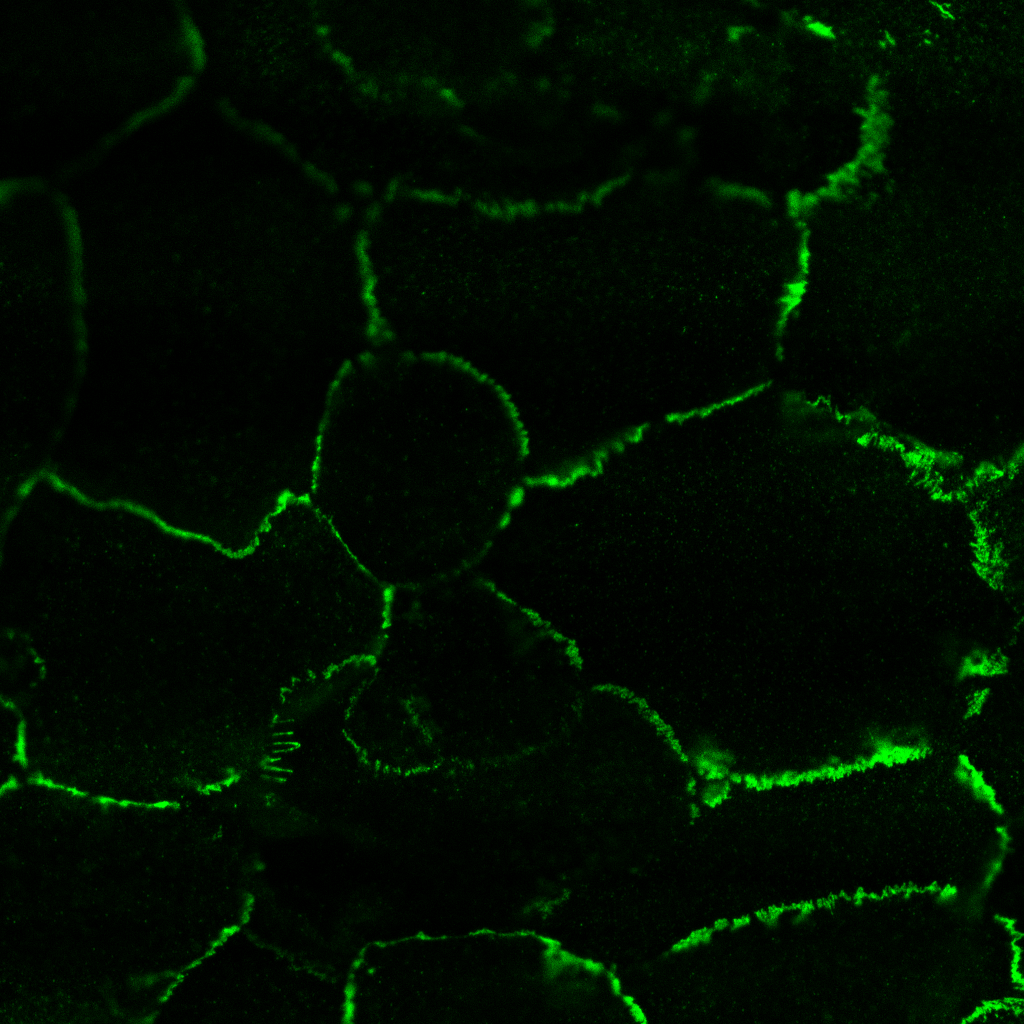

Supplement: Supplementary file 5 — Source data Fig. 2 [file 44321_2024_104_MOESM5_ESM.zip › Figure 2/2F/OCLN siNEG/siNEG OCLN.tif]

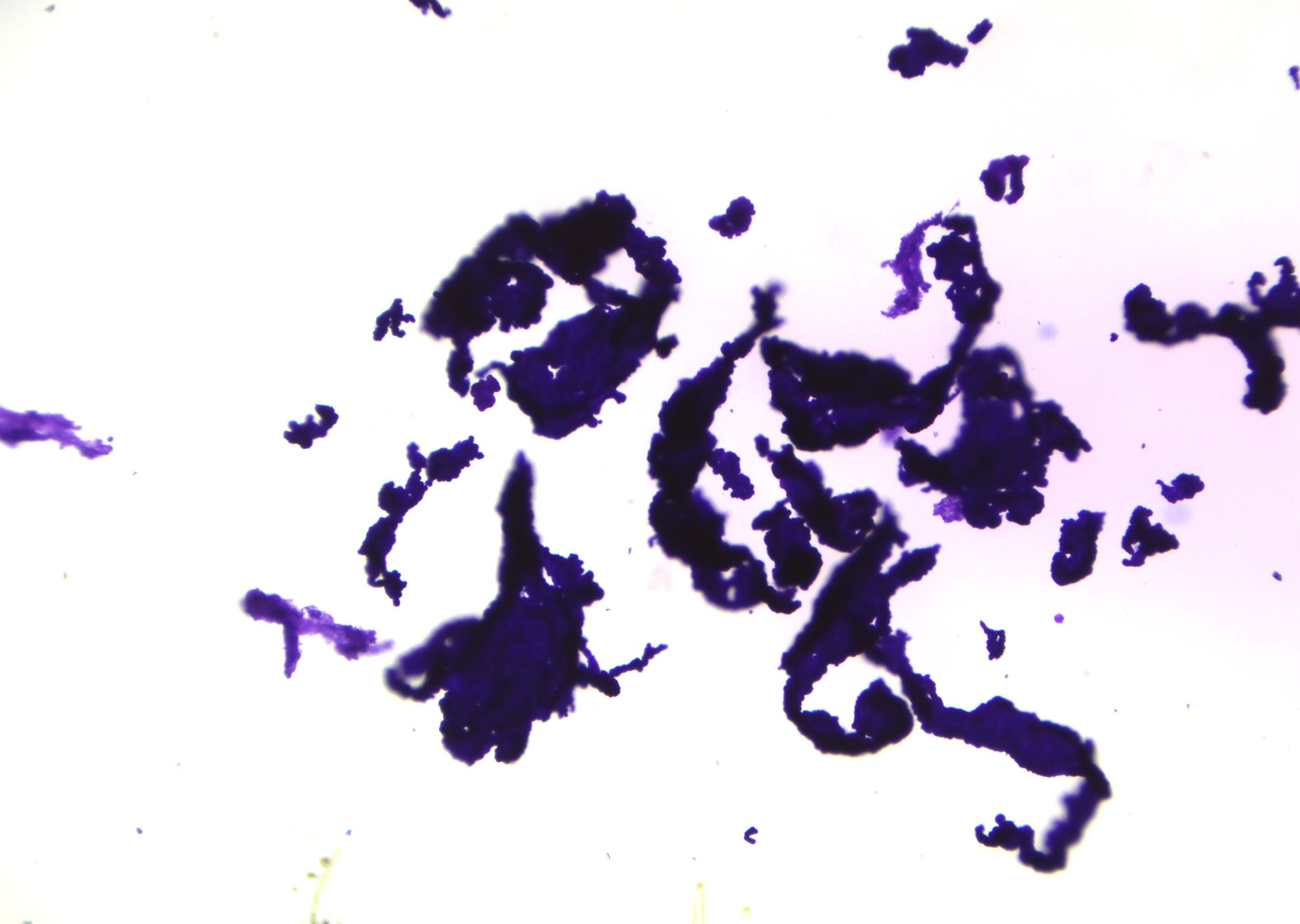

Supplement: Supplementary file 5 — Source data Fig. 2 [file 44321_2024_104_MOESM5_ESM.zip › Figure 2/2H/Tg 100nM + Dl.jpg]

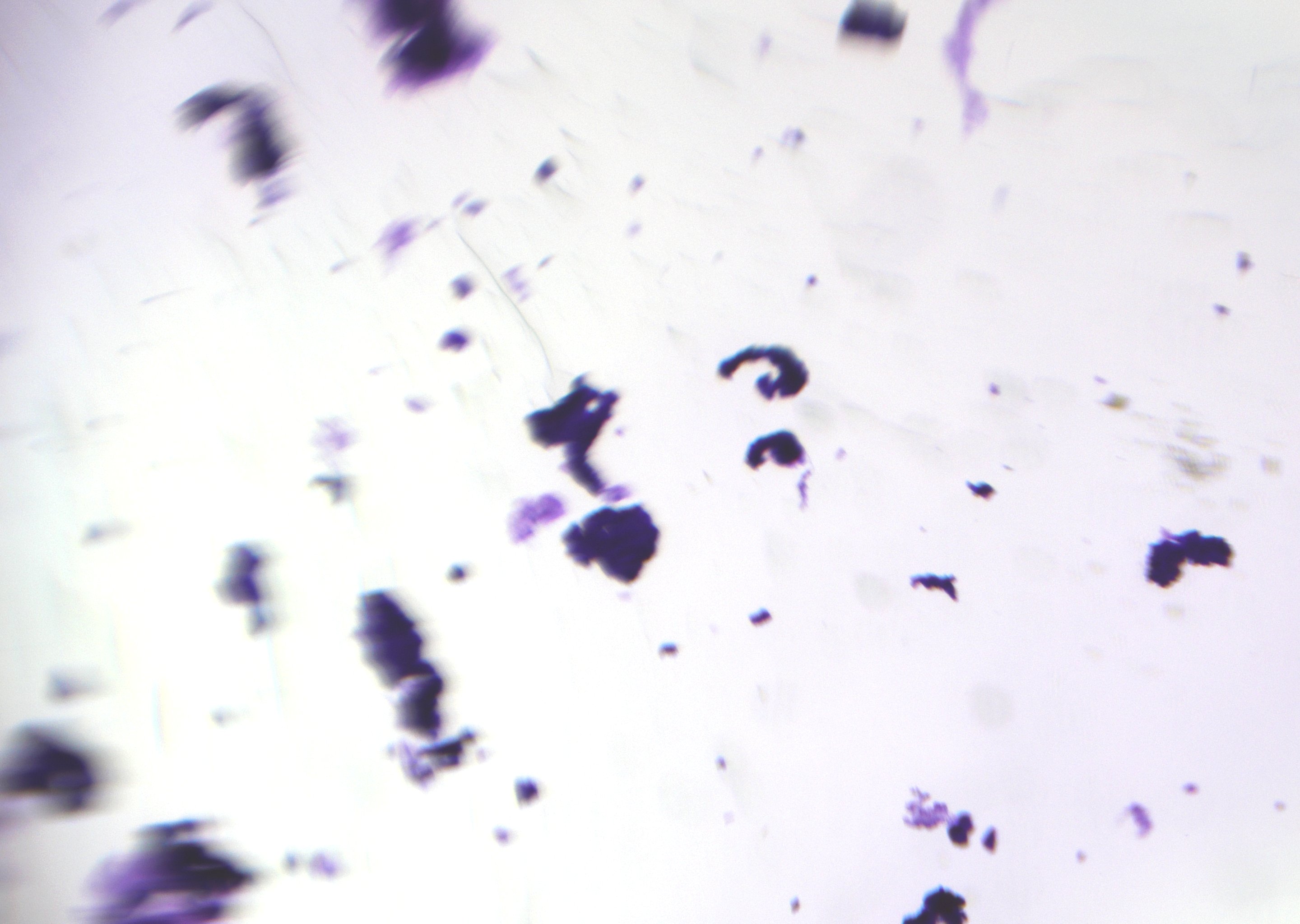

Supplement: Supplementary file 5 — Source data Fig. 2 [file 44321_2024_104_MOESM5_ESM.zip › Figure 2/2H/Tg 100nM.jpg]

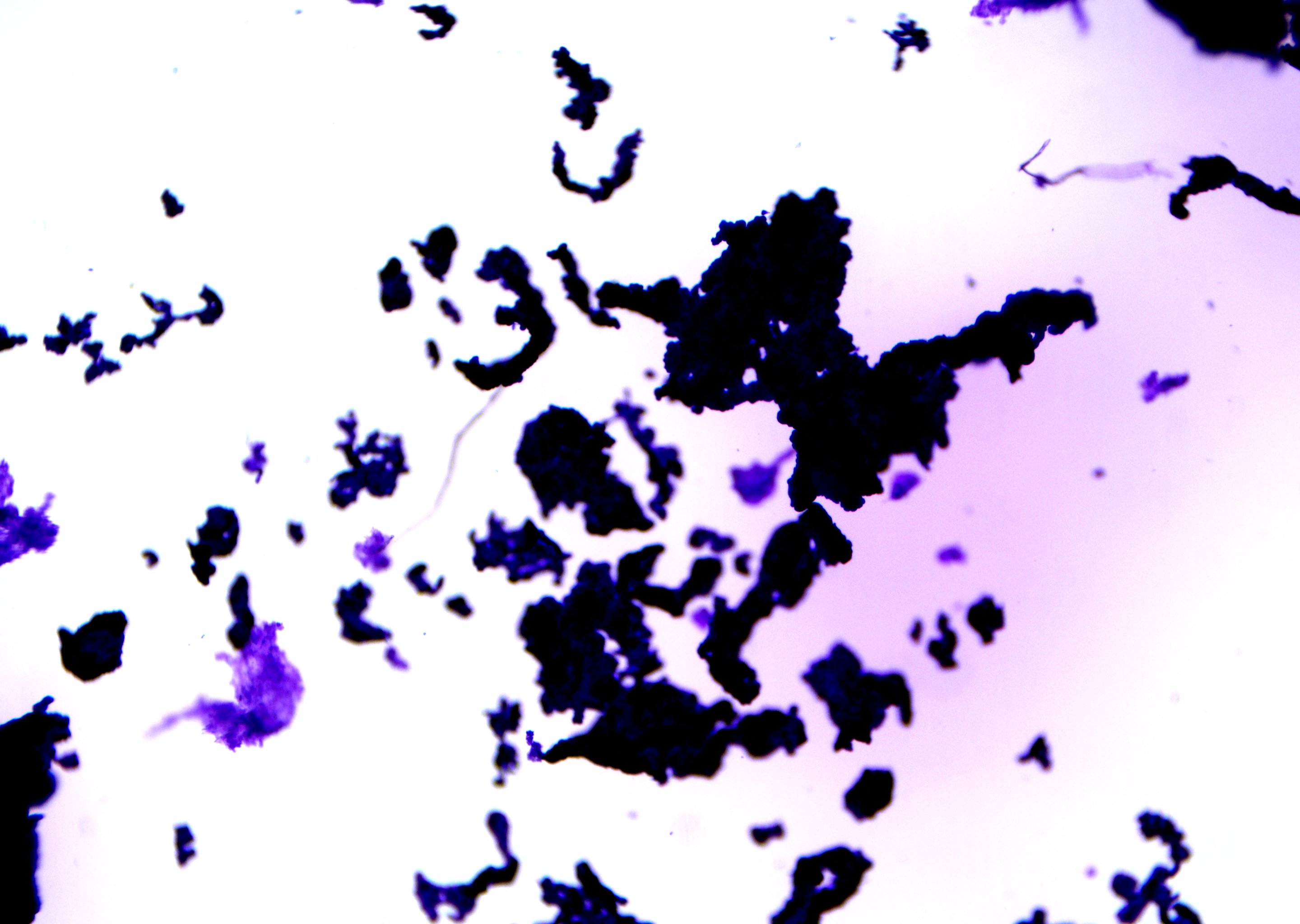

Supplement: Supplementary file 5 — Source data Fig. 2 [file 44321_2024_104_MOESM5_ESM.zip › Figure 2/2H/Tg 10nM + Dl.jpg]

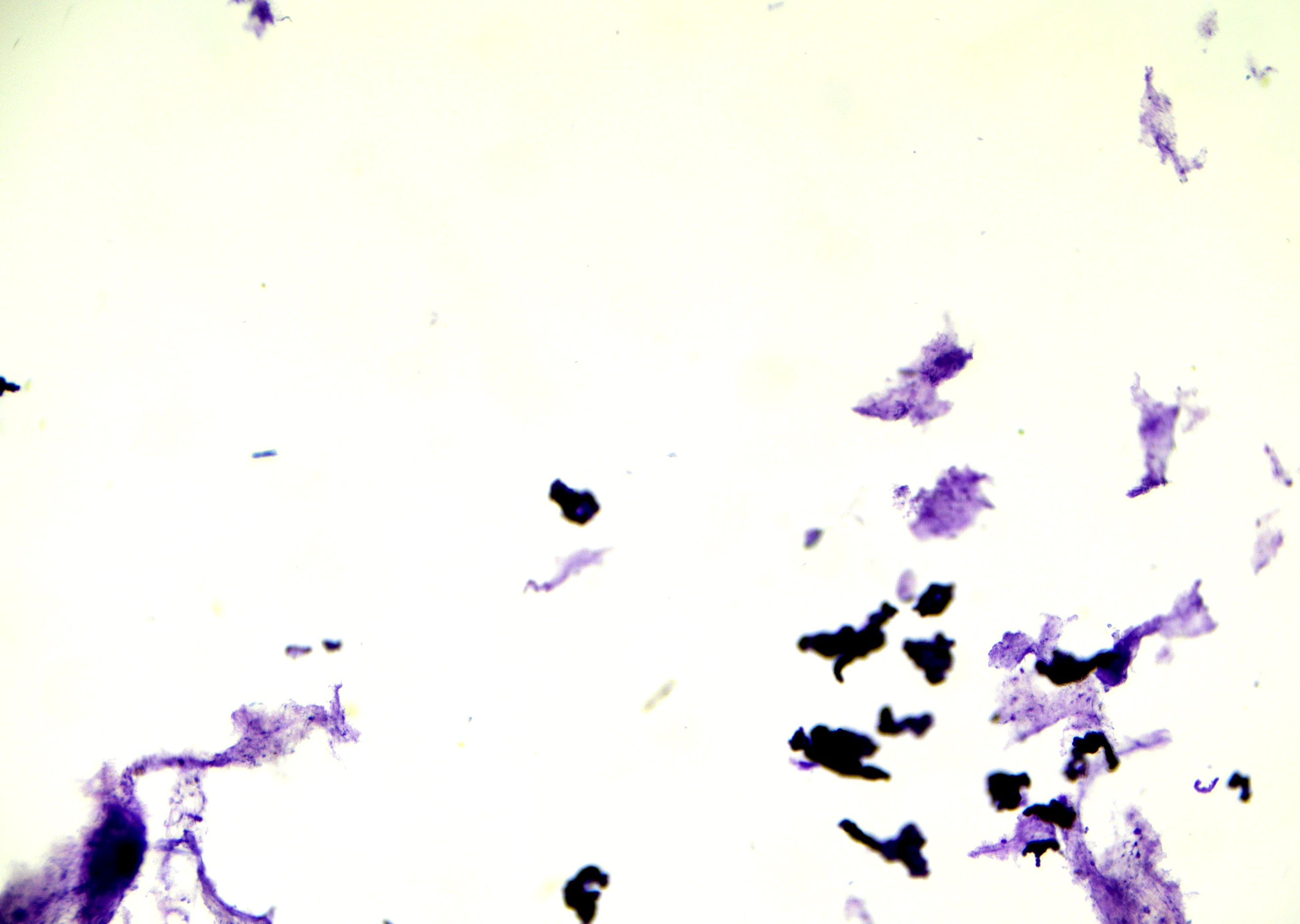

Supplement: Supplementary file 5 — Source data Fig. 2 [file 44321_2024_104_MOESM5_ESM.zip › Figure 2/2H/Tg 10nM.jpg]

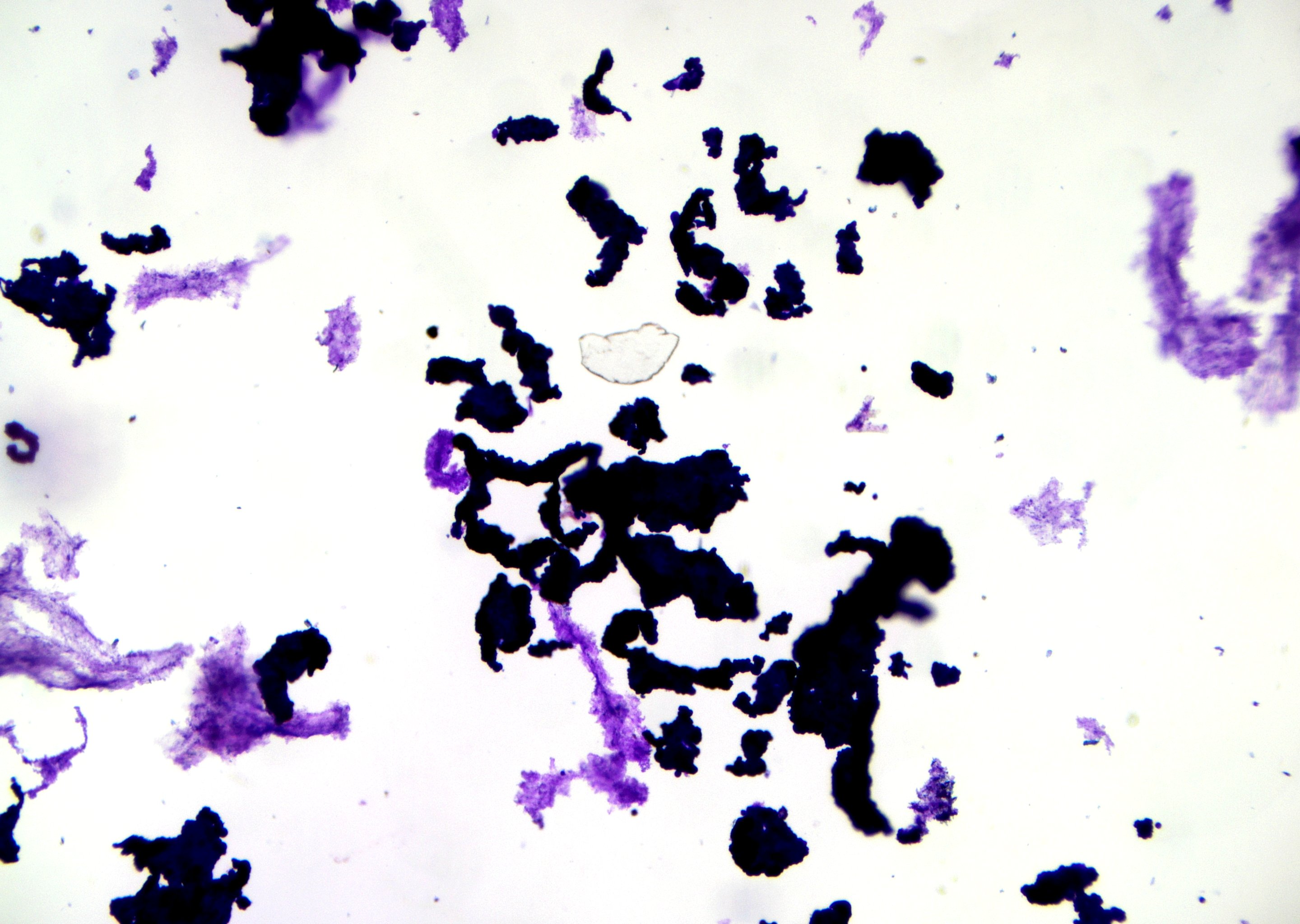

Supplement: Supplementary file 5 — Source data Fig. 2 [file 44321_2024_104_MOESM5_ESM.zip › Figure 2/2H/Tg 20nM + Dl.jpg]

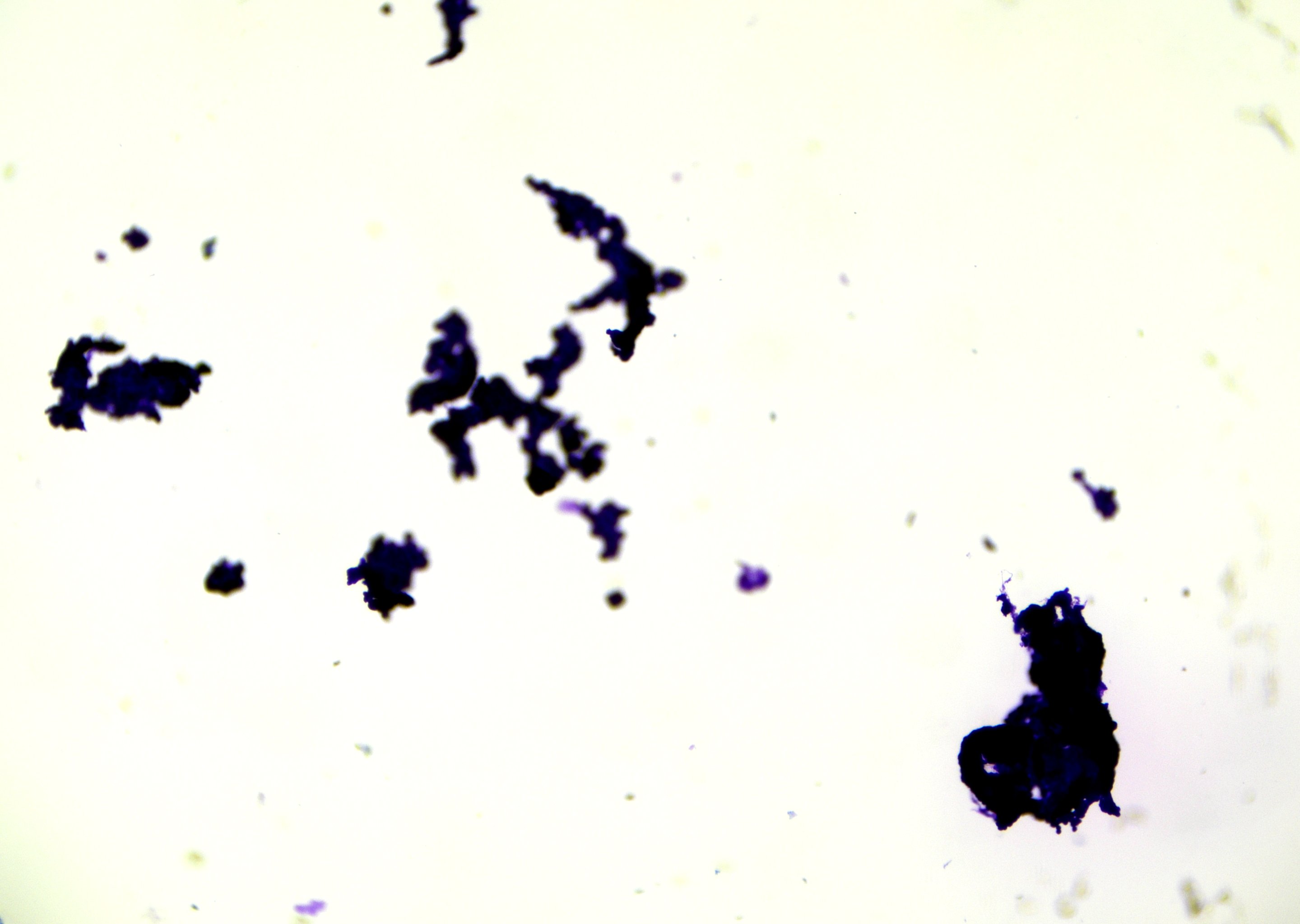

Supplement: Supplementary file 5 — Source data Fig. 2 [file 44321_2024_104_MOESM5_ESM.zip › Figure 2/2H/Tg 20nM.jpg]

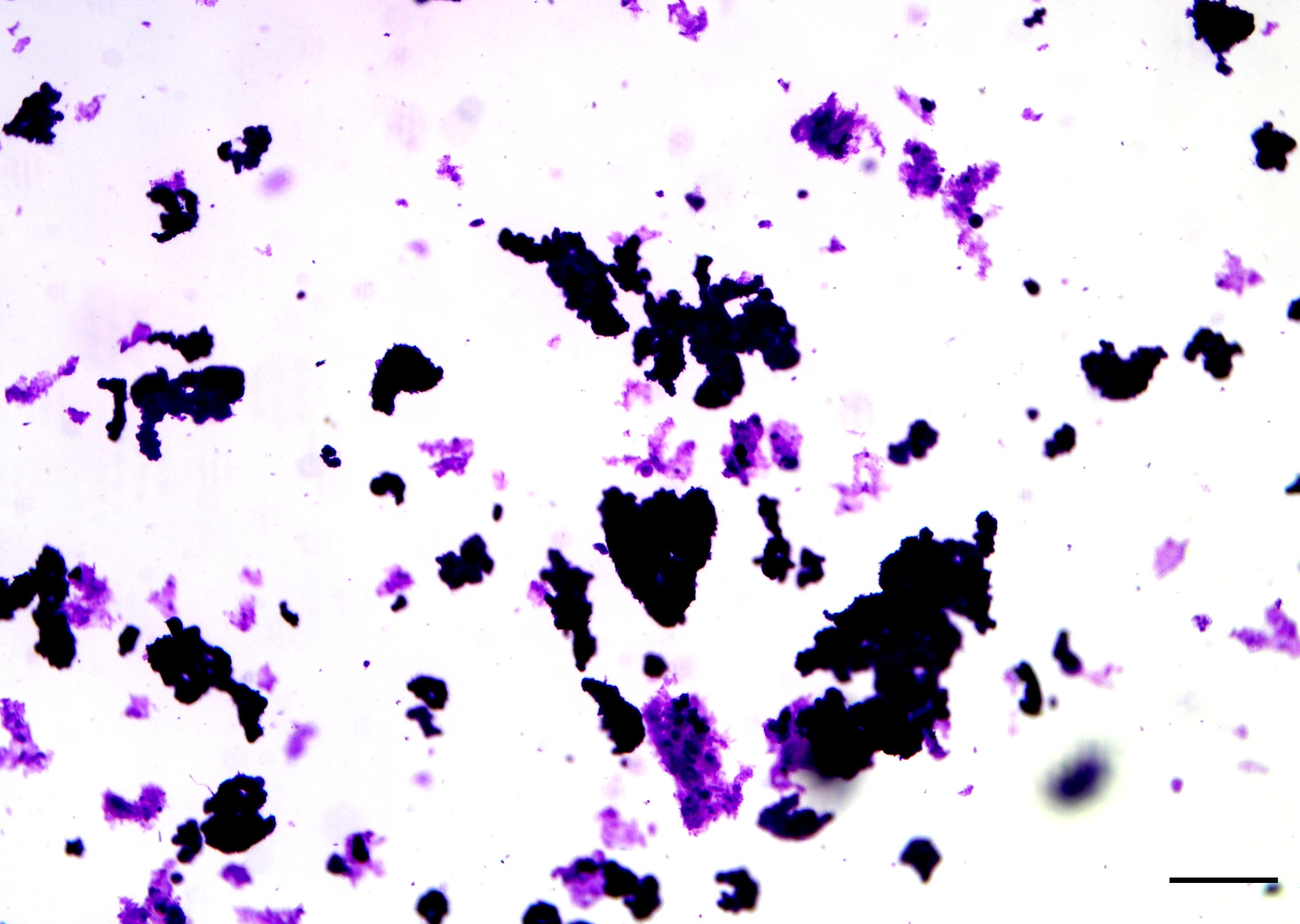

Supplement: Supplementary file 5 — Source data Fig. 2 [file 44321_2024_104_MOESM5_ESM.zip › Figure 2/2H/Veh Dl-.jpg]

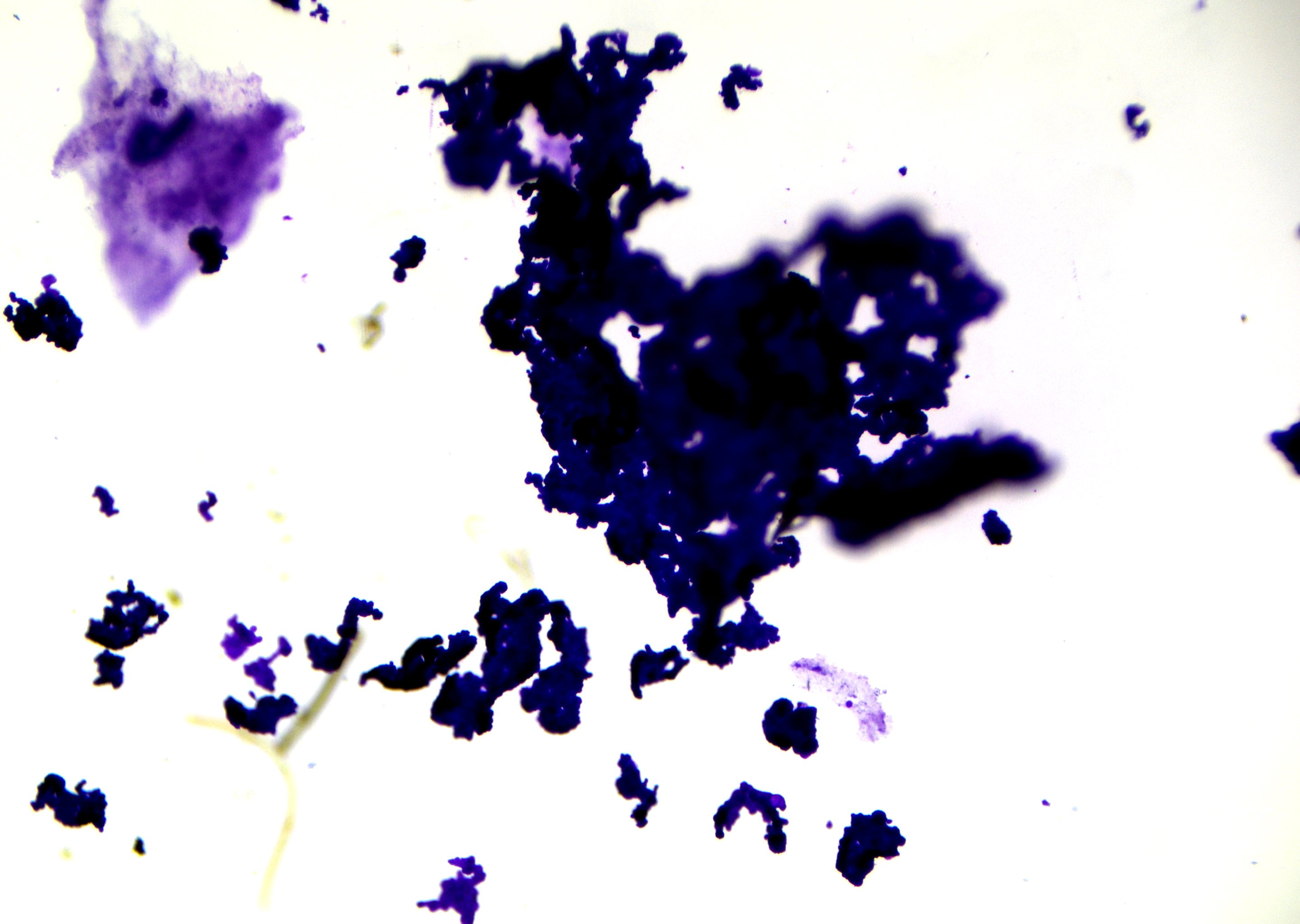

Supplement: Supplementary file 5 — Source data Fig. 2 [file 44321_2024_104_MOESM5_ESM.zip › Figure 2/2H/Veh Dl+.jpg]
