# Supplementary figures and images for: Dantrolene corrects cellular disease features of Darier disease and may be a novel treatment (part 2 of 2)
Source: EMBO Mol Med. 2024 Jul 26;16(9):1986–2001. doi: 10.1038/s44321-024-00104-3 (PMC11392931; doi:10.1038/s44321-024-00104-3)

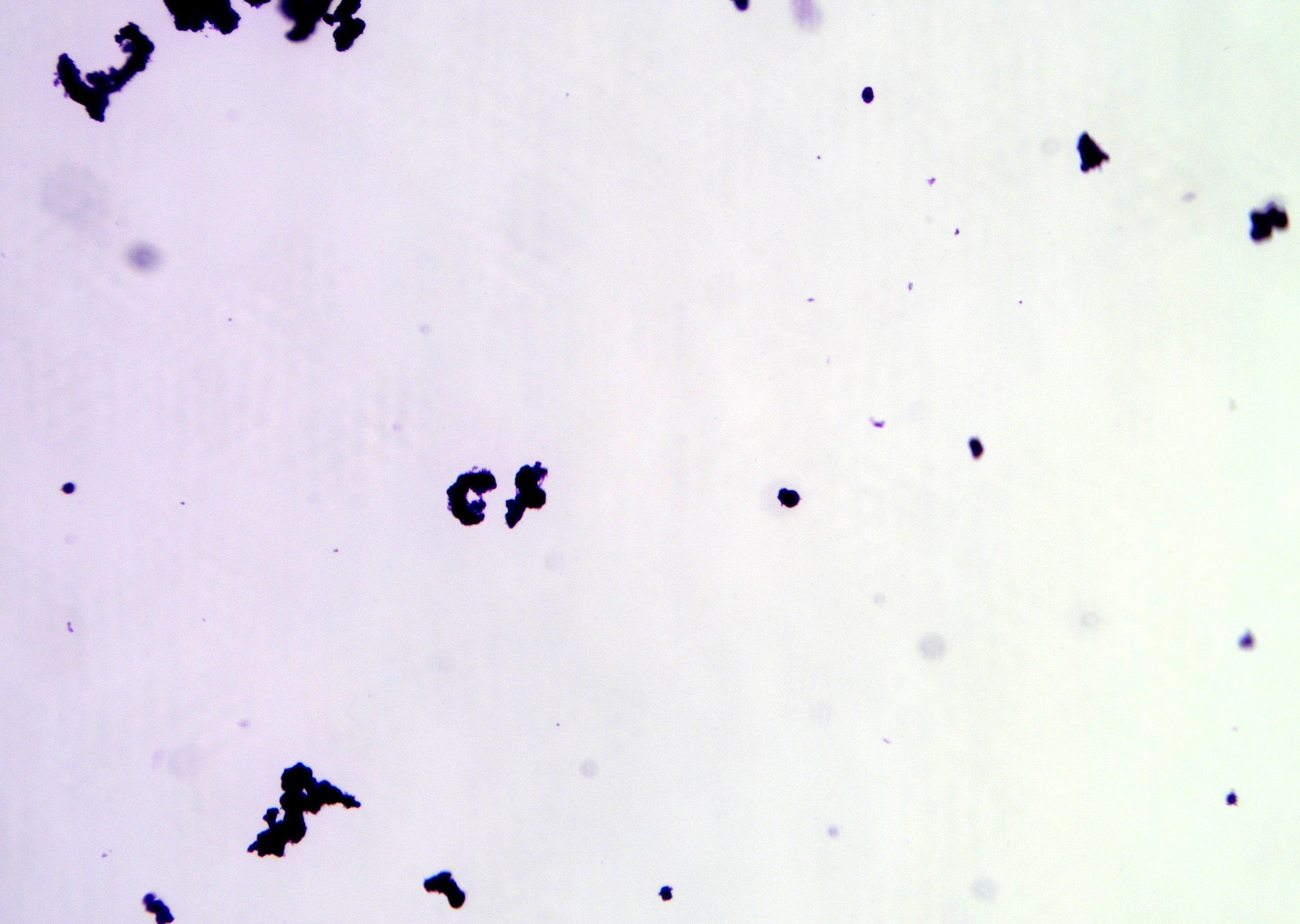

Supplement: Supplementary file 5 — Source data Fig. 2 [file 44321_2024_104_MOESM5_ESM.zip › Figure 2/2J/siATP2A2 Dl-.jpg]

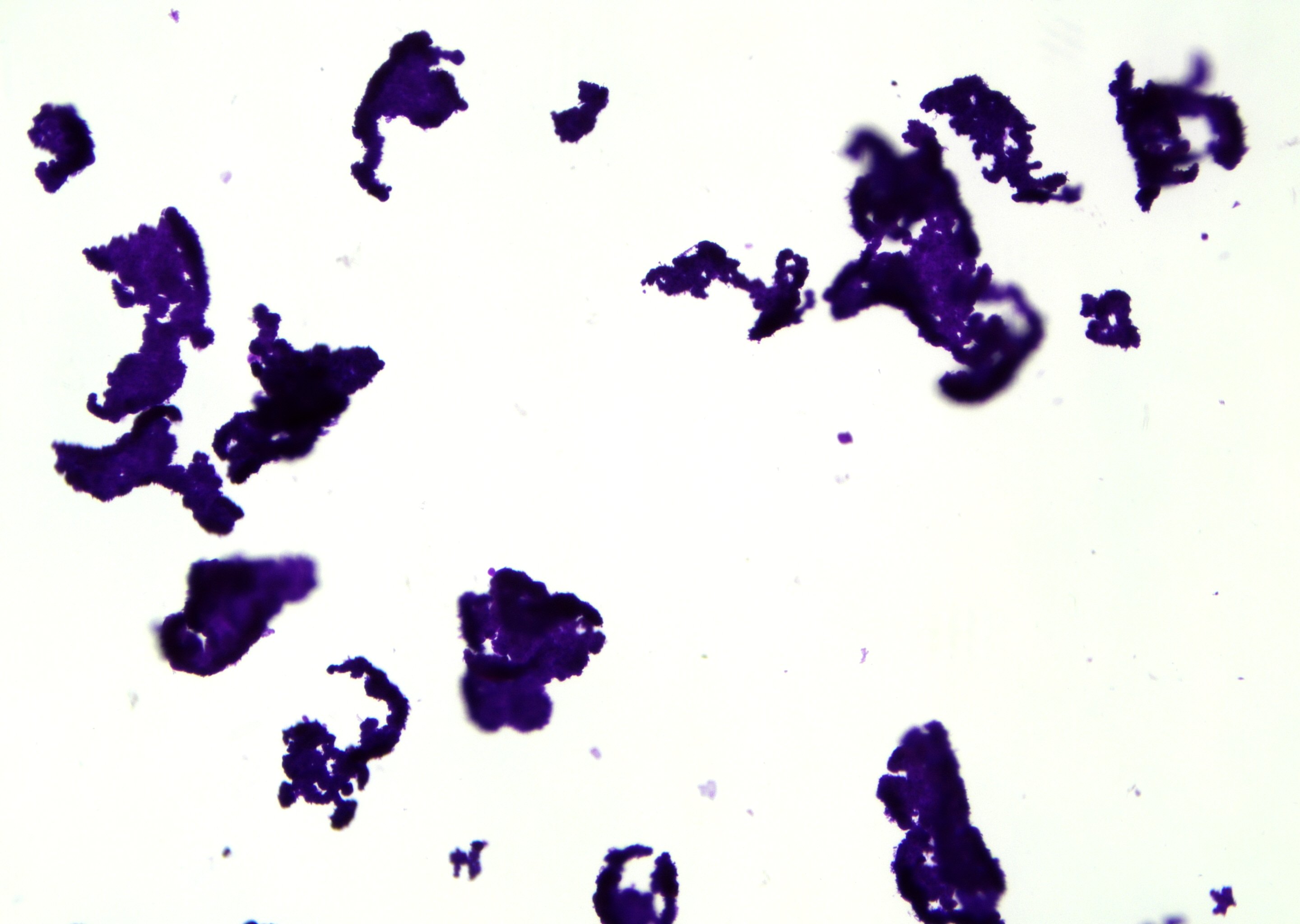

Supplement: Supplementary file 5 — Source data Fig. 2 [file 44321_2024_104_MOESM5_ESM.zip › Figure 2/2J/siATP2A2 Dl+.jpg]

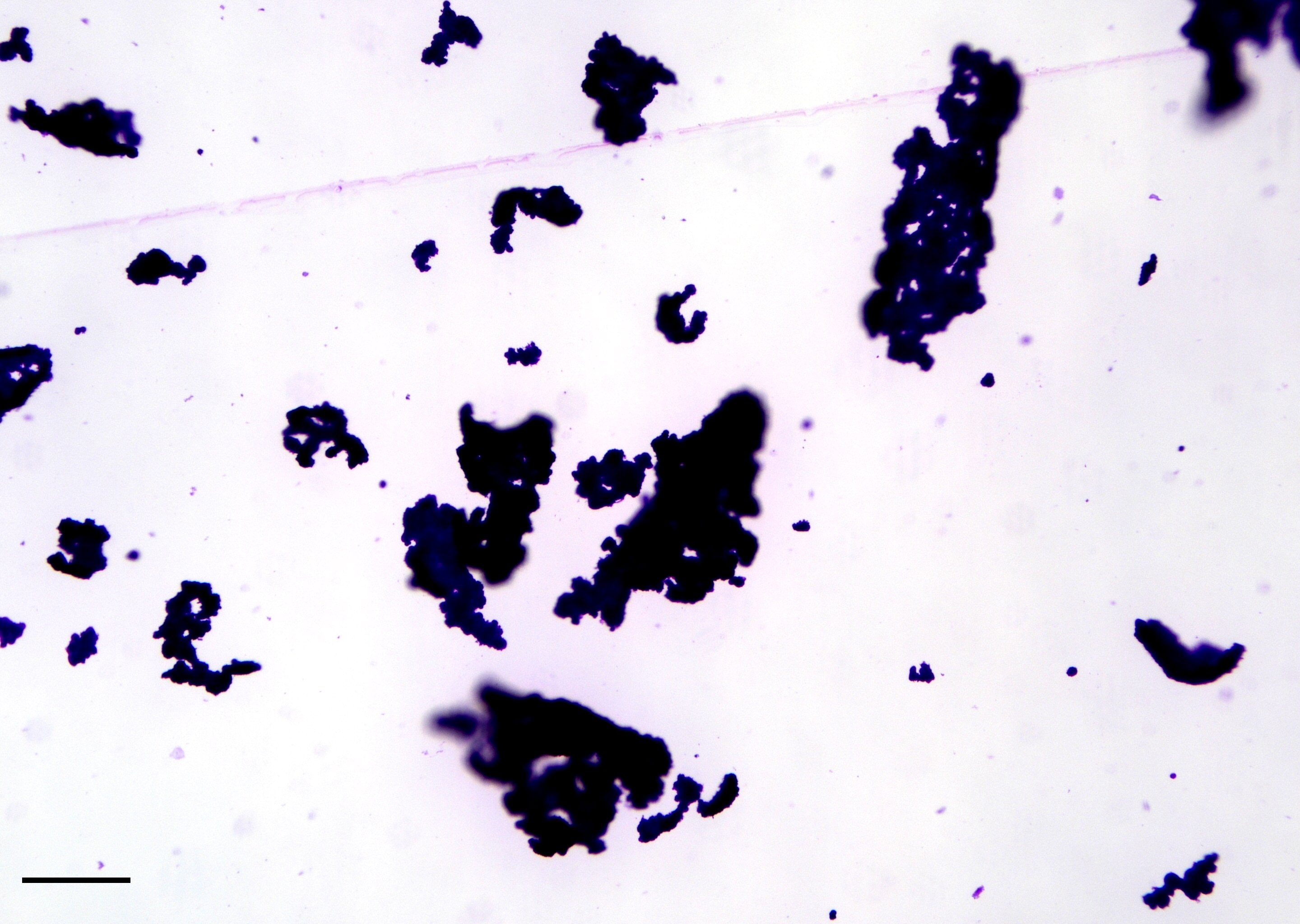

Supplement: Supplementary file 5 — Source data Fig. 2 [file 44321_2024_104_MOESM5_ESM.zip › Figure 2/2J/siNEG Dl-.jpg]

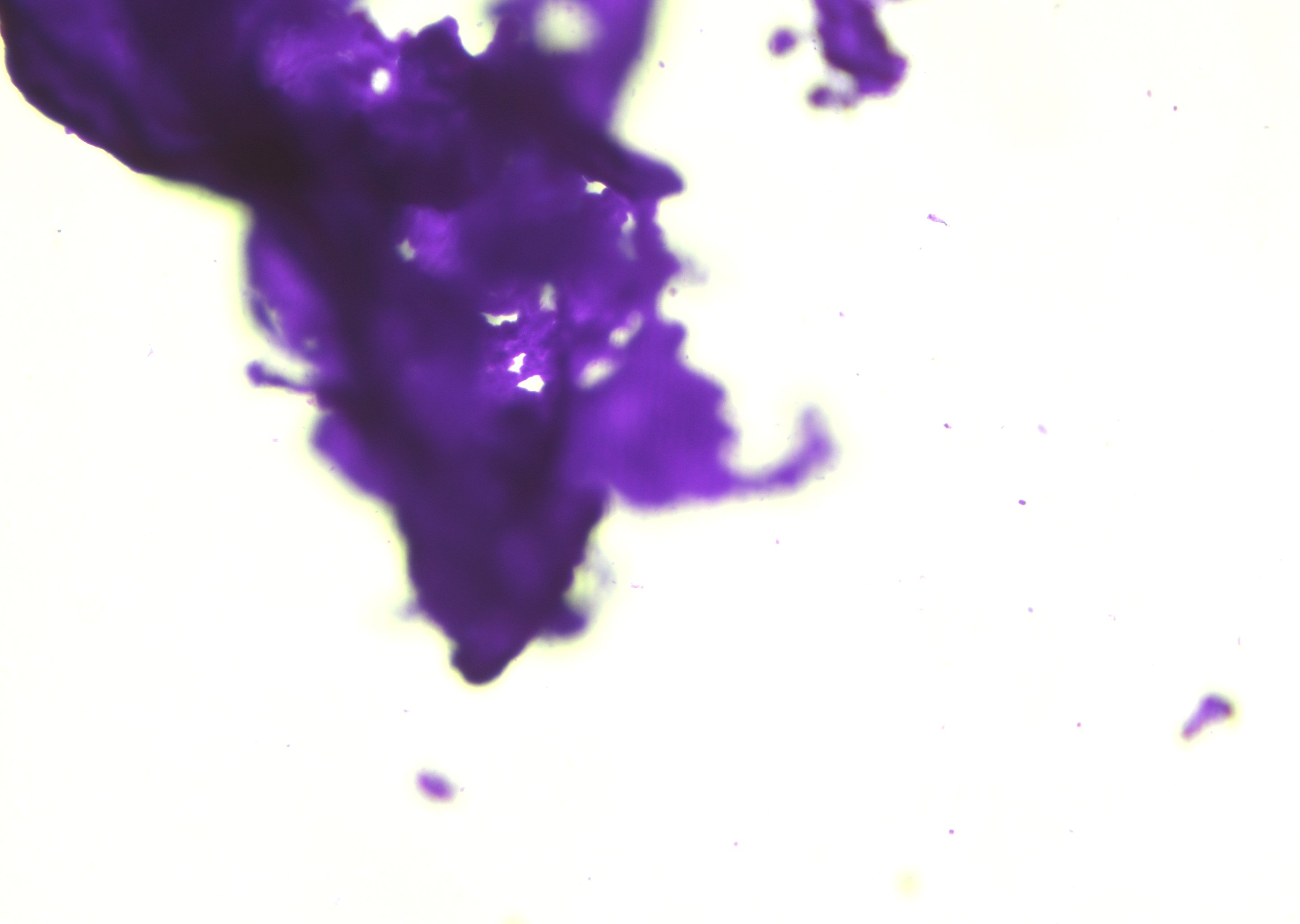

Supplement: Supplementary file 5 — Source data Fig. 2 [file 44321_2024_104_MOESM5_ESM.zip › Figure 2/2J/siNEG Dl+.jpg]
