# Supplementary material for: Dantrolene corrects cellular disease features of Darier disease and may be a novel treatment
Source: EMBO Mol Med. 2024 Jul 26;16(9):1986–2001. doi: 10.1038/s44321-024-00104-3 (PMC11392931; doi:10.1038/s44321-024-00104-3)
Supplement: Supplementary file 8 — Expanded View Figures [file 44321_2024_104_MOESM8_ESM.pdf]

Expanded View Figures

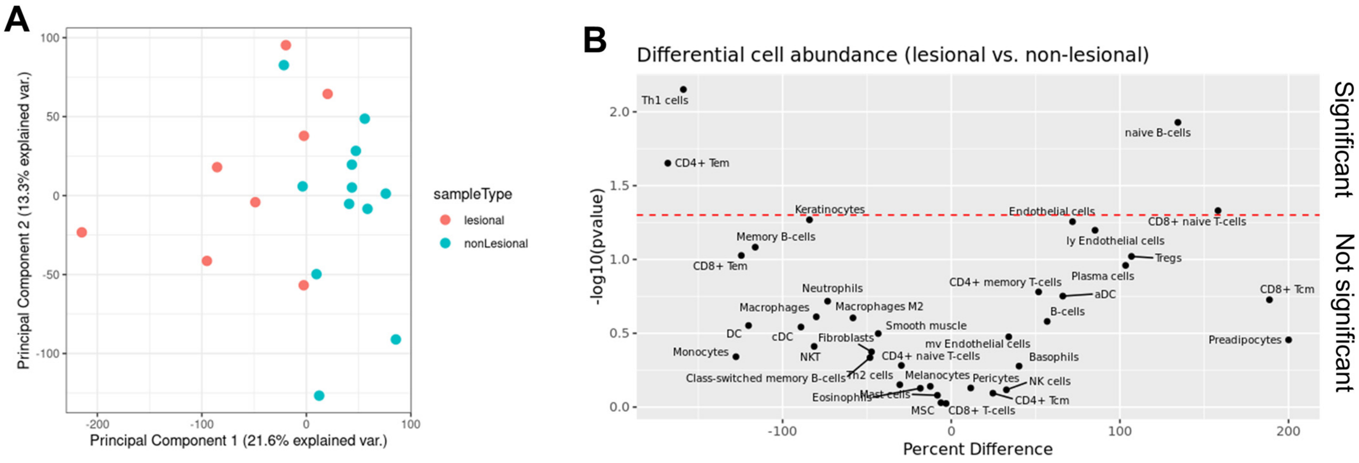

C

Pathway	padj	log2err	NES	Size
GO:0140053 Mitochondrial gene expression	3.62E-14	1.086441	-2.79493	161
GO:0032543 Mitochondrial translation	8.46E-14	1.06721	-2.88019	130
GO:0001525 Angiogenesis	3.53E-13	1.037696	2.175602	457
GO:0090287 Regulation of cellular response to growth factor stimulus	5.90E-10	0.921426	2.22513	261
GO:0034470 ncRNA processing	5.98E-10	0.921426	-2.06419	414
GO:0001501 Skeletal system development	1.45E-09	0.898671	2.017345	426
GO:0060537 Muscle tissue development	1.50E-09	0.898671	2.118144	315
GO:0045229 External encapsulating structure organization	1.50E-09	0.898671	2.176517	261
GO:0071559 Response to transforming growth factor beta	1.50E-09	0.898671	2.252733	234
GO:0043062 Extracellular structure organization	1.50E-09	0.898671	2.195713	259

Figure EV1. RNAseq analysis of lesional and non-lesional DD skin biopsies.

(**A**) Principal component analysis (PCA) plots based on mRNA expression profiles. Each dot represents an individual patient. Red = lesional skin; blue = non-lesional skin. (**B**) Table of the top ten most significantly differently expressed Gene Ontology (GO) pathways in lesional vs non-lesional skin. NES = normalized enrichment score; size = number of genes in each respective pathway. (**C**) Volcano plot showing differential cell abundance of cell types between lesional and non-lesional biopsies derived from deconvolution analysis. Statistical significance determined through paired *t* tests.

 © The Author(s)

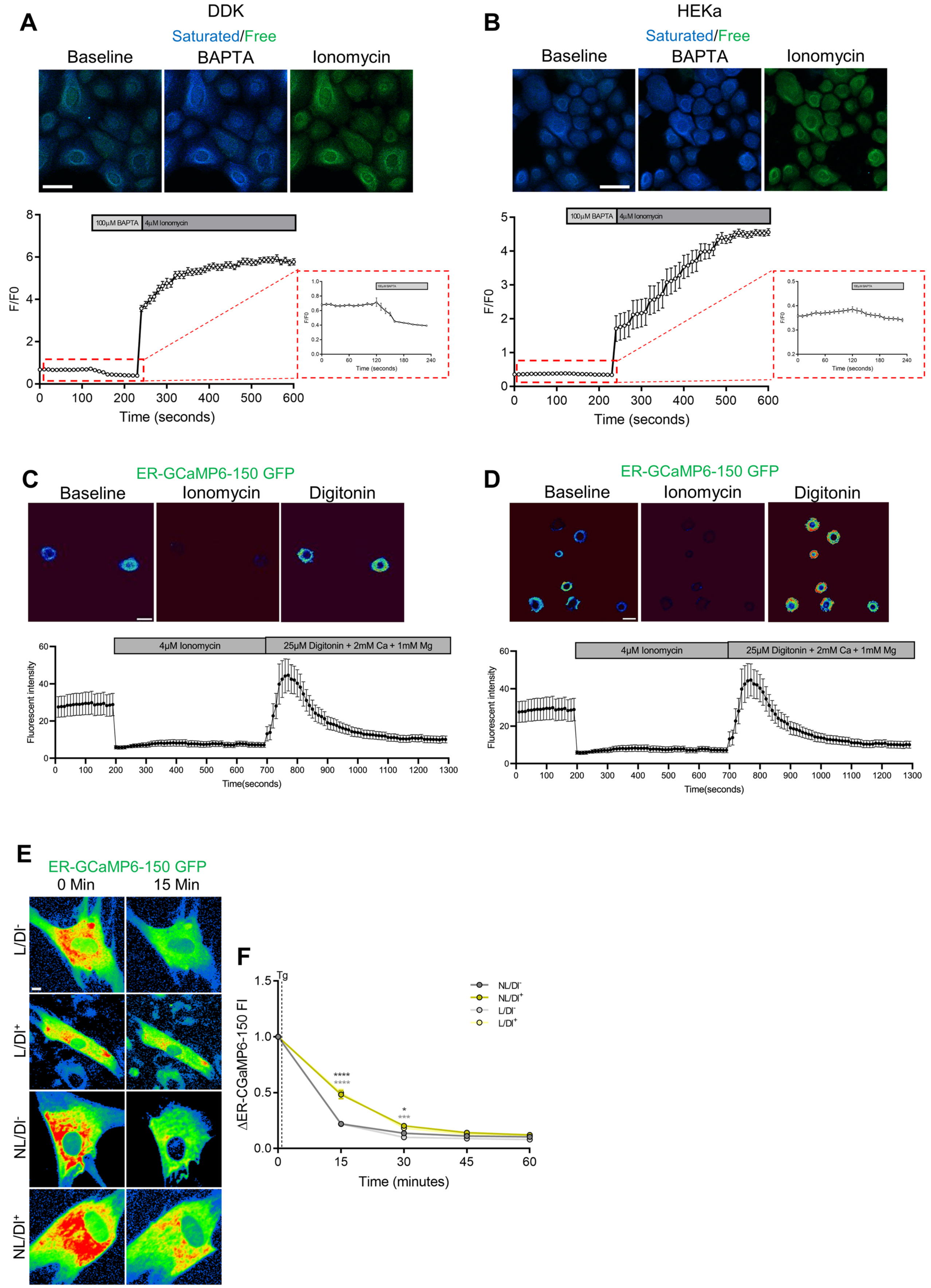
A
DDK
Saturated/Free
Baseline
BAPTA
Ionomycin
100µM BAPTA
4µM Ionomycin
F/F0
Time (seconds)
B
HEKa
Saturated/Free
Baseline
BAPTA
Ionomycin
100µM BAPTA
4µM Ionomycin
F/F0
Time (seconds)
C
ER-GCaMP6-150 GFP
Baseline
Ionomycin
Digitonin
4µM Ionomycin
25µM Digitonin + 2mM Ca + 1mM Mg
Fluorescent intensity
Time(seconds)
D
ER-GCaMP6-150 GFP
Baseline
Ionomycin
Digitonin
4µM Ionomycin
25µM Digitonin + 2mM Ca + 1mM Mg
Fluorescent intensity
Time(seconds)
E
ER-GCaMP6-150 GFP
0 Min
15 Min
L/DI-
L/DI+
NL/DI-
NL/DI+
F
Tg
ΔER-CGaMP6-150 FI
Time (minutes)
NL/DI-
NL/DI+
L/DI-
L/DI+

Figure EV2. Ca^{2+} live cell imaging calibration.

(**A**, **B**) Graph and representative images of Fura Red cytosolic Ca^{2+} calibration in (**A**) DDK and (**B**) Tg HEKa. Scale bars = 25 μm. (**C**, **D**) Graph and representative images of ER-Ca^{2+} calibration in (**C**) DDK and (**D**) Tg HEKa. Scale bar = 25 μm. (**E**) Representative pseudocoloured images of ER-Ca^{2+} imaging in DDF. Colors are adjusted in the same manner. Scale bar = 10 μm. (**F**) Graph (mean ± SEM) of ER-Ca^{2+} fluorescence in DDF. Two-way ANOVA; ****$P < 0.0001$; ***$P \leq 0.001$; *$P \leq 0.05$. Source data are available online for this figure.

 © The Author(s)

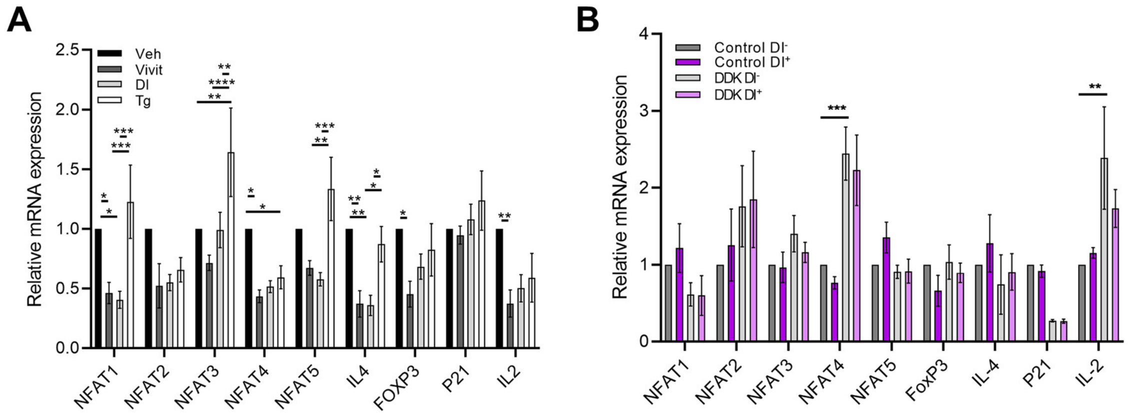

Figure EV3. Gene expression analysis of NFAT markers.

(**A**, **B**) RT-qPCR quantification (mean ± SEM) of relative mRNA gene expression of different *NFAT* markers in (**A**) HEKa cells and (**B**) DDK cells. Vivit was used as a negative control to inhibit *NFAT* marker expression. *P* values were calculated by two-way ANOVA; $^{****}P \leq 0.0001$; $^{***}P \leq 0.001$; $^{**}P \leq 0.01$; $^{*}P \leq 0.05$; $N = 3$ biological replicates containing 3 technical replicates for all conditions. Source data are available online for this figure.

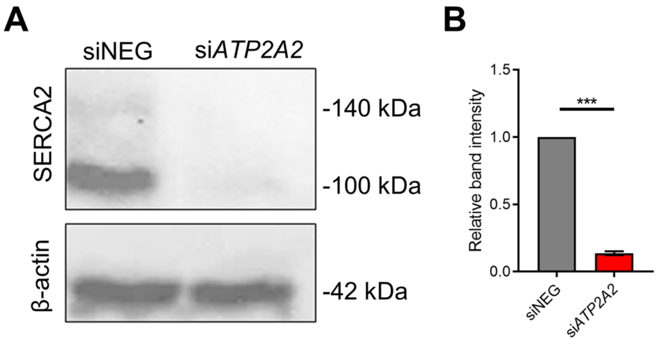

Figure EV4. Optimization of *ATP2A2* knockdown.

(**A**) Representative immunoblots depicting SERCA2 downregulation in HEKa cells transfected with either siNEG or si*ATP2A2*. (**B**) Relative band intensity quantification (mean ± SEM) of SERCA2 protein expression after normalization to β-actin. *P* values two-way ANOVA; $^{***}P \leq 0.001$. $N = 3$ biological replicates.

© The Author(s)
